# Bayesian Analysis Reporting Guidelines

**DOI:** 10.1038/s41562-021-01177-7

**Published:** 2021-08-16

**Authors:** John K. Kruschke

**Affiliations:** Department of Psychological and Brain Sciences, Indiana University, Bloomington, Bloomington, IN USA

**Keywords:** Medical research, Psychology

## Abstract

Previous surveys of the literature have shown that reports of statistical analyses often lack important information, causing lack of transparency and failure of reproducibility. Editors and authors agree that guidelines for reporting should be encouraged. This Review presents a set of Bayesian analysis reporting guidelines (BARG). The BARG encompass the features of previous guidelines, while including many additional details for contemporary Bayesian analyses, with explanations. An extensive example of applying the BARG is presented. The BARG should be useful to researchers, authors, reviewers, editors, educators and students. Utilization, endorsement and promotion of the BARG may improve the quality, transparency and reproducibility of Bayesian analyses.

## Abstract (web summary)

The Review presents a comprehensive set of Bayesian analysis reporting guidelines (BARG), incorporating features of previous guidelines and extending these with many additional details for contemporary Bayesian analyses. It is accompanied by an extensive example of applying the BARG.

## Main

Statistical analyses can be conceptually elaborate and procedurally complex, and therefore it is easy to skip steps in the execution of the analysis and to leave out important information in reporting the analysis. These problems can result in erroneous or incomplete analyses and in reports that are opaque and not reproducible. Bayesian analyses might be especially prone to these problems because of their relative novelty among applied researchers. The concern is pressing because Bayesian analyses are promoted as having important advantages over traditional frequentist approaches^1^ and are being used in increasing numbers of publications in the behavioural sciences^2^.

In a review^3^ of the reporting of Bayesian analyses for medical devices, using the ROBUST (reporting of Bayes used in clinical studies) checklist^4^ for scoring, only 24% of 17 articles fully reported the prior, only 18% reported a sensitivity analysis, only 35% explained the model, and only 59% reported credible intervals. In a review^5^ of reporting of mixed-treatment comparisons analysed with Bayesian methods, only 52.9% of 34 articles reported the prior distribution, only 11.8% reported a sensitivity analysis, only 35.3% reported Markov chain Monte Carlo (MCMC) convergence measures, and only 20.6% made their computer code available. In a review^6^ of Bayesian meta-analyses of *N*-of-1 studies, using the ROBUST checklist^4^ for scoring, 5 out of 11 reviewed articles scored 7 out of 7 on the ROBUST list, and the remaining 6 articles scored 6 out of 7. In most cases, all that was missing (according to the ROBUST criteria) was a sensitivity analysis. However, only 3 of the 11 articles mentioned convergence diagnostics, no articles mentioned effective sample size (ESS), and only 2 articles made the computer code available. In an extensive review of applied Bayesian analyses^2^, 55.6% out of 99 articles did not report the hyperparameters specified for the prior, 56.6% did not report checking for chain convergence, and 87.9% did not conduct a sensitivity analysis on the impact of priors^7^. A review^8^ of 70 articles in epidemiologic research using Bayesian analysis found that 2 did not specify a model, 9 did not specify the computational method, 14 did not specify what software was used, 27 did not report credible intervals, 33 did not specify what prior was used, and 66 did not report a sensitivity analysis, leading the authors to conclude that “We think the use of checklists should be encouraged and may ultimately improve the reporting on Bayesian methods and the reproducibility of research results”^8^.

Journal editors and authors agree that reporting guidelines should be encouraged^9^. In a survey of editors and authors^10^ regarding the use of the guidelines for transparent reporting of evaluations with nonrandomized designs (TREND)^11^, most editors believed that all authors and reviewers should use reporting guidelines. Editors agreed that reporting guidelines need to be promoted by journals and by professional societies. Authors felt that they would be encouraged if peers used the guidelines. In the findings, the authors recommended^10^ that there should be future research to demonstrate the efficacy of guidelines, which would also encourage their adoption.

Several previous guidelines for Bayesian analyses have been published^4,7,12–22^. Many consisted of cursory points with relatively little explanation and left out details that are important for transparency and reproducibility of contemporary analyses, such as aspects of MCMC diagnostics, different ways of reporting decisions about null values, and what to post online for genuine reproducibility. A more detailed review of the previous guidelines is presented in the Supplementary Information.

In developing BARG, I incorporated insights from these previous recommendations and included many additional details and explanations in a sequential structure. I also drew heavily from personal experience as a researcher who uses Bayesian analyses to understand data, as a reviewer who evaluates Bayesian analyses for transparency and reproducibility, and as an instructor of Bayesian courses and workshops with audiences across the social, biological and physical sciences, and across business and industry.

Many researchers believe that they already fulfil all the important steps of guidelines, even if they do not know any guidelines or explicitly follow them^23^. Unfortunately, this belief is frequently not well founded, as revealed by surveys of the literature described above. Therefore researchers, authors, reviewers and editors could find value in the BARG, and educators and students could also benefit by teaching and learning why each item in the BARG is important^24^.

## What is not on the BARG

The BARG recommend essential items that should be reported from a Bayesian analysis, but do not aspire to review best practices for conducting an analysis^19^. Despite not being a catalogue of best practices, the BARG help the analyst think carefully and thoroughly about all the steps of the analysis, and therefore to pursue best practices.

The BARG assume that the researcher is being forthright in reporting all relevant data and all relevant analyses, without biased culling of inconvenient findings. There are a wide variety of selection processes that can bias the results that are reported. Selectively reporting only statistically significant effects produces the ‘file drawer problem’^25^, by which the published literature overrepresents spurious effects and underrepresents weak or null effects. A corollary is ‘hypothesizing after the results are known’^26^, which artificially converts exploratory research to confirmatory. Any kind of data sifting, model tweaking or limited selection of tests done with the goal of driving the frequentist *P* value to significance is often called ‘*p* hacking’^27^. A variety of ways to select data or results in a drive for significance are collectively called ‘questionable research practices’^28^ and the ‘garden of forking paths’^29^. Many of the same kinds of selection biases can affect Bayesian analyses. There are Bayesian approaches to alleviating some of these problems, such as providing methods for accepting null values and not merely rejecting them, but the BARG are not designed to prevent such questionable research practices.

The BARG aid complete execution and reporting of the analyses chosen by the researcher, but are not intended to guide the design of studies or the reporting of designs. Nevertheless, there are Bayesian approaches to various design issues, such as adaptive design of experiments and clinical trials^30^ and sample size planning^15,31,32^.

There are different Bayesian approaches to evaluating null values, and the BARG attempt to be inclusive. In particular, the BARG describe reporting practices for interval-estimation and hypothesis-testing approaches. A note on terminology: a ‘null value’ is the value of a parameter that indicates a null effect, such as an effect size of zero. By contrast, a ‘null hypothesis’ is an entire model that has the relevant parameter fixed at its null value. An analyst can ‘assess a null value’ by estimating the parameter value and considering the relation of its posterior distribution to its null value. An analyst can ‘test a null hypothesis’ by comparing a full model with a restricted model that fixes the parameter at the null value. Hypotheses with other forms of parameter restrictions^33^ are also accommodated. The literature discusses other approaches to evaluating null values^34–36^ that are not explicitly included in the BARG, and future developments in statistical practice may produce still others. Regardless of the specific method used, it should be able to decide in favour of a null and not only against a null.

The BARG do not provide specific templates or formats for reporting the details of analyses because there are far too many different types of models to cover, because Bayesian software encourages novel models for which there is no conventional format and because conventional formats can become obsolete. Instead, the BARG provide general guidelines that address essentials of every analysis.

## The BARG steps

The BARG identify essential items that should be reported from any Bayesian data analysis. There are six ordered steps preceded by a preamble, each with key items to report, as listed in Table 1. The steps are explained in the following sections.

**Table 1:** List of key reporting points for the BARG

**Preamble**
A. *Why Bayesian*. If the audience requires it, explain what benefits will be gleaned by a Bayesian analysis (as opposed to a frequentist analysis). B. *Goals of analysis*. Explain the goals of the analysis. This prepares the audience for the type of models to expect and how the results will be described.
**Step 1. Explain the model**
A. *Data variables*. Explain the dependent (predicted) variables and independent (predictor) variables. B. *Likelihood function and parameters*. For every model, explain the likelihood function and all the parameters, distinguishing clearly between parameters of primary theoretical interest and ancillary parameters. If the model is multilevel, be sure that the hierarchical structure is clearly explained, along with any covariance structure if multivariate parameter distributions are used. C. *Prior distribution*. For every model, explain and justify the prior distribution of the parameters in the model. D. *Formal specification*. Include a formal specification (mathematical or computer code) of the likelihood and prior, located either in the main text or in in publicly and persistently accessible online supplementary material. E. *Prior predictive check*. Especially when using informed priors but even with broad priors, it is valuable to report a prior predictive check to demonstrate that the prior really generates simulated data consistent with the assumed prior knowledge.
**Step 2. Report details of the computation**
A. *Software*. Report the software used, including any specific added packages or plugins. B. *MCMC chain convergence*. Report evidence that the chains have converged, using a convergence statistic such as PSRF, for every parameter or derived value. C. *MCMC chain resolution*. Report evidence that the chains have high resolution, using the ESS, for every parameter or derived value. D. *If not MCMC.* If using some computational procedure other than MCMC, be aware of and report inherently inaccurate approximations, especially for the limits of credible intervals.
**Step 3. Describe the posterior distribution**
A. *Posterior predictive check*. Provide a posterior predictive check to show that the model usefully mimics the data. B. *Summarize posterior of variables*. For continuous parameters, derived variables and predicted values, report the central tendency and limits of the credible interval. Explicitly state whether you are using density-based values (mode and HDI) or quantile-based values (median and ETI), and state the mass of the credible interval (for example, 95%). C. *BF and posterior model probabilities*. If conducting model comparison or hypothesis testing, report the BF and posterior probabilities of models for a range of prior model probabilities.
**Step 4. Report decisions (if any) and their criteria**
A. *Why decisions?* Explain why the decisions are theoretically meaningful and which decision procedure is being used. Regardless of which decision procedure is used, if it addresses null values, it should be able to accept the null value not only reject it. B. *Loss function*. If utilities and a loss function for a decision rule are defined, these should be explained and reported. C. *ROPE limits*. If using a continuous-parameter posterior distribution as the basis for decision, state and justify the limits of the ROPE and the required probability mass. D. *BF, decision threshold and model probabilities*. If using model comparison or hypothesis testing as the basis for a decision, state and justify the decision threshold for the posterior model probability, and the minimum prior model probability that would make the posterior model probability exceed the decision threshold. E. *Estimated values too*. If deciding about null values, always also report the estimate of the parameter value (central tendency and credible interval).
**Step 5. Report sensitivity analysis**
A. *For broad priors*. If the prior is intended to be vague or only mildly informed so that it has minimal influence on the posterior, show that other vague priors produce similar posterior results. B. *For informed priors*. If the prior is informed by previous research, show what posterior results from a vague prior or from a range of differently informed priors. C. *For default priors*. If using a default prior, show the effect of varying its settings. Be sure that the range of default priors constitutes theoretically meaningful priors, and consider whether they mimic plausible empirically informed priors. D. *BFs and model probabilites*. If the analysis involves model comparison or hypothesis testing, then for each prior report not only the BFs but also the posterior model probabilities for a range of prior model probabilities. E. *Decisions*. If making decisions, report whether decisions change under different priors. For BFs, report changes in the minimum prior model probability needed to achieve decisive posterior model probability.
**Step 6. Make it reproducible**
A. *Software and installation*. Explain all the software that is necessary and where to obtain it. If possible, use non-proprietary software. B. *Software version details*. The posted script should include detailed information about the software version numbers. C. *Script and data*. Post the complete analysis script (that is, computer code) and data in a stable public repository with persistent URLs, so that anyone can download it and exactly reproduce the analysis. Be sure that it is clear how to navigate the site and find relevant files, for example, with a wiki overview or readme file. If posting data, be sure that it respects privacy and copyright restrictions. If the original data cannot be posted publicly, it may be helpful to post dummy data of the same form so that users can verify the operation of the analysis script. D. *Readable for humans*. Make the posted script genuinely readable by human beings. Annotate the code with thorough explanatory comments and spatially arrange the code for human readability. E. *All auxiliary files*. Check that all the needed auxiliary files (utility scripts, image files, bibliography files, formatting files and so on) are also posted. F. *Runs as posted*. Check that the posted script and accompanying files run as is when downloaded to a different computer. The code should have no lines that load files from personal computer directories or non-persistent URLs. G. *MCMC chains for time-intensive runs*. For MCMC runs that take a long time to compute, it is helpful to post an MCMC chain so that people can inspect the MCMC chain without having to wait through an entire run duration. H. *Reproducible MCMC*. To make MCMC chains exactly reproducible, the pseudo-random number generators should be explicitly seeded.

These points are discussed in the main text, and an extended example is presented in Supplementary Information (also at https://osf.io/w7cph/). When reporting an analysis, the points on this list should be addressed in the main text or in appendices and supplementary material. The specific sequential ordering of points is suggested, and not a requirement. BF, Bayes factor; ESS, effective sample size; ETI, equal-tailed interval; HDI, highest-density interval; MCMC, Markov chain Monte Carlo; PSRF, potential scale reduction factor; ROPE, region of practical equivalence.

The contents of the key points (Table 1) should be included somewhere in the report, but whether each point is reported in the main text or in appendices or supplementary materials depends on the specific outlet and audience. Some outlets have severe constraints on word count, or audiences who want an emphasis on domain theory and not on details of the analysis, which might be best served by summaries in the main text and further details in appendices or supplementary materials.

## Example of applying the BARG

An example of applying the BARG is provided in the Supplementary Information (and is also available at https://osf.io/w7cph/). The example considers the star ratings of two films, and evaluates the differences of their means and variances using parameter estimation and hypothesis testing. Despite the simplicity of the application, the write-up is quite extensive and provides an elaborate illustration of details from the BARG. The example is an essential component of this article, and the reader is strongly encouraged to consult it.

## Preamble

Ideally, the report should motivate the use of Bayesian methods and explain the goals of the analyses (Table 1).

Some audiences are not familiar with Bayesian analysis, and appreciate an explanation of why the analysis is not frequentist^31,37,38^. In the report, the benefits of Bayesian analysis, rather than the perceived shortcomings of frequentist approaches, should be emphasized. One of the benefits of Bayesian analysis is its flexibility in specifying models that are appropriate for the data. Another important benefit of Bayesian analysis is the ability to generate estimates and credible intervals for any derived parameter or predicted variable. Differences, ratios, effect sizes and novel parameter combinations or predicted quantities are directly computed from the posterior distribution (see the example in the Supplementary Information). Another benefit of Bayesian analysis is computationally robust estimates of parameter values and their credible intervals. The credible intervals do not depend on large-*N* approximations (as confidence intervals often do in frequentist approaches), nor do credible intervals depend on which tests are intended (as confidence intervals do in frequentist approaches). If there is hypothesis testing, another key strength of Bayesian analysis is that it provides methods for quantifying support in favour of the null hypothesis, and not only against the null hypothesis.

The goals of the analysis frame the expression of the model and results of the analysis. Most applications are covered by three types of goals listed below, where each subsequent goal builds on the preceding goal.

### Description, including measurement

Data are described by mathematical models, and the analysis finds parameter values that best mimic the data. The parameter values, in the context of the model, describe the data. In measurement, the parameter values are thought to refer not merely to a pattern in the data but to a characteristic of the natural mechanism that generated the data. An essential aspect of description or measurement is quantifying the uncertainty of the estimated parameter values. The uncertainty is expressed by a credible interval on continuous values or on the probabilities of discrete values.

### Prediction, including model averaging

In some applications (for example, any type of forecasting) a key goal is prediction of dependent variables for candidate values of independent variables. How far the model is intended to extrapolate beyond the observed data should be made explicit. In most applications, prediction is based on a descriptive model with meaningfully interpreted parameters. An exception is ‘black box’ models, which have so many parameters and such complex model structure that the model cannot be interpreted meaningfully as a descriptive or measurement model, and is instead used for prediction only. In a Bayesian setting, prediction can take advantage of multiple models by taking a posterior-weighted average of the predictions, as in Bayesian model averaging^39^. In prediction, an essential aspect is quantifying the uncertainty of the predicted values. The uncertainty is expressed by a credible interval on continuous values or on the probabilities of discrete values.

### Formal model selection, including null hypothesis testing

In some applications, it is a goal to select a best model or hypothesis from a defined set of discrete possibilities. Model selection may be a primary goal when models are of structurally different types and compete to explain a domain. Tests of full versus restricted nested models may seek parsimonious descriptions^40^. For example, in multiple regression, analysts might pursue variable selection for which the goal is to select predictors that achieve a balance of parsimony and fit to the data. Formal null-hypothesis testing is especially relevant when the null value of a parameter is qualitatively distinct from any small non-null values. Analysts should be careful, however, to avoid unnecessary ritualistic null-hypothesis testing, which can lead to cognitive errors and publication bias^41–43^.

## Explaining the model

The foundational idea of statistical analysis, both frequentist and Bayesian, is that data are understood through a mathematical model that mimics the data. A mathematical model is a machine that generates random values around a trend. The machine has control knobs, called parameters, that determine the location of the trend and the spread of the randomly generated data around that trend. For example, numerical data that when plotted appear as a unimodal histogram might be described as a normal distribution, which is a mathematical function that has (1) a mean parameter that specifies the trend location and (2) a standard deviation parameter that specifies the spread of random values around the trend. In this example, the data are understood through the normal distribution that mimics it. The essential goal of statistical analysis is to find a mathematical model and its parameter settings that usefully mimic the data, along with the uncertainty of those parameter settings. Therefore, every report of a statistical analysis (whether Bayesian or frequentist) must clearly explain the mathematical model and all of its parameters.

In Bayesian analysis (but not in frequentist analysis), an additional fundamental idea is that different parameter values have different credibilities, and the credibility of each parameter value can be represented by a relative probability value. Across the range of parameter values there is a probability distribution that represents the relative credibility of each parameter value. When the probability distribution on a parameter is very broad, there are many parameter values that are all weakly credible, which represents high uncertainty about the value of the parameter. Conversely, when the probability distribution on a parameter is peaked over a narrow range of values, the narrowness represents low uncertainty about the value of the parameter.

A Bayesian statistical analysis begins with a prior probability distribution across all the parameters. Data from the research at hand are then incorporated into the analysis, and the probability distribution is shifted towards parameter values that are relatively consistent with the data (and shifted away from parameter values that are relatively inconsistent with the data). The re-allocated credibility across parameter values is called the posterior distribution. In many routine applications, the prior distribution is very broad to represent unbiased uncertainty, but in some applications, the prior distribution is informed by knowledge from previous research. Because the prior distribution can influence the posterior distribution, every report of a Bayesian analysis must clearly explain the prior distribution on the parameters.

Formally, a model in Bayesian analysis includes both the likelihood function, which expresses the probability of data given the parameter values, and the prior probability distribution, which expresses the probability of the parameter values before taking into account the novel data. For example, for data modelled by a normal distribution, the likelihood function indicates that the data *y* are distributed as a normal distribution with mean *μ* and standard deviation *σ*, which is written formally as y ~ normal(*μ*, *σ*), where ‘~’ means ‘is distributed as’. The prior distribution on the parameters could take many forms, but one typical prior could specify *μ* ~ normal(0, 10) and *σ* ~ lognormal(0, 10), where ‘lognormal’ refers to the log-normal distribution. The constants in the prior distribution are set to reflect prior knowledge of the domain, which may be broad and uncertain. The description of the likelihood and prior should include formal details expressed either mathematically or as well-annotated computer code. These details could be in the main document or in supplementary material.

When an analysis includes multiple models, as in model comparison or hypothesis testing, all of the models need to be clearly explained, including their likelihood functions, parameters and prior distributions. Moreover, the prior probabilities of the models or hypotheses should also be discussed.

Because the posterior distribution can sometimes depend strongly on the choice of prior distribution, a key part of reporting priors is justifying the choice. That is, it is not sufficient merely to state what prior was used, there must be a rationale for the prior. Two further steps are involved in justifying a prior: a prior predictive check and a sensitivity analysis. A prior predictive check displays simulated data that are generated from parameter values in the prior distribution. The simulated data from the mathematically specified prior should show trends that match the trends assumed by prior knowledge. A sensitivity analysis considers how much the posterior distribution changes when the prior is changed in relevant ways. Sensitivity analysis is discussed in BARG step 5.

Because a key part of reporting priors is justifying them, and justification entails considerations of best practices, this discussion now ventures into best practices for setting priors. However, best practices are evolving, and what is noted here may be superseded in the future. Moreover, there may be trade-offs of costs and benefits among different practices, and experts may disagree on what is best for any particular application.

When estimating continuous parameters and using a broad prior intended to express great uncertainty, it should be confirmed that the prior really is broad on the particular scale of the data. For example, a prior that is broad on the scale of hair diameter might not be broad on the scale of distance between galaxies. When the prior is intended to be informed by previous data or theory, it should be checked that it really does accurately represent the previous data or theory. This is called a prior predictive check^19,44^ because simulated data generated from the prior distribution are examined and checked for consistency with the previous data or theory. One way of creating an informed prior is to use the posterior distribution from a set of representative data and a very broad initial prior. The example in the Supplementary Information demonstrates this technique and a prior predictive check.

When conducting model comparisons or hypothesis tests, the prior distributions within each model must be carefully considered so that the comparison is meaningful. A common approach to model comparison uses default priors that satisfy certain mathematical consistency properties^45–48^. One benefit of this approach is computational efficiency. Another benefit is conventionality, which allows default priors to be implemented in packaged software. Moreover, with default priors, there is limited ability of different analysts to choose arbitrary or idiosyncratic priors, and there is enhanced efficiency of communication among those who are familiar with the default conventions. However, the default priors might not accurately capture the prior information or theory needed by the analyst. Thus, it is important to justify that the default priors constitute theoretically or empirically meaningful models for the specific application.

Another useful approach to setting priors of multiple models is to inform every model with the same small set of representative prior data. “Empirical regularities for … phenomena are often well established. These regularities provide an accessible and substantial source of information for constructing priors”^49^. The representative data could be either actual previous data or a fictitious small set of data that accurately represent previously observed trends. In this approach, every model is initially given a very broad prior which is then updated with the representative data. The resulting posterior is used as the informed prior for the target data at hand. “The idea of using a small amount of training data to inform the priors for model comparison has been discussed at length in the literature and is an active research topic. A selective overview was provided by Berger and Pericchi^50^, who discussed conventional default priors^46^, ‘intrinsic’ Bayes factors (BFs)^51^, and ‘fractional’ BFs^52,53^, among others”^15^. Both intrinsic and fractional BFs take a small subset of the data to update a broad prior into an informed prior, and then use the remaining bulk of the data to compute the BF. “… we begin by sacrificing some data to construct informed priors. We will then show that the model with informed priors make more constrained, and arguably more sensible predictions than when we use vague priors”^54^. Instead of using a subset of the current data, priors can be informed by previous data. An illustration of using a small set of representative previous data to inform priors of all models is provided in the example in the Supplementary Information. This technique of informing all models with the same representative data is greatly facilitated by the availability of a computational technique called bridge sampling^55,56^. Bridge sampling works for arbitrary models and does not require simplifying assumptions required by Jeffrey’s default priors^46,47^ or the Savage–Dickey method^57^.

Finally, when doing model comparison or hypothesis testing, it is important to consider the prior probabilities of the models or hypotheses. In practice, the prior probabilities of the models or hypotheses are routinely ignored because they can be uncertain. However, if decisions about models are to be based on their posterior probabilities, then the prior probabilities of the models should be considered. This issue of prior and posterior model probabilities is discussed further in BARG steps 3 and 4.

Reporting points for this step are included in Table 1.

## Reporting details of the computation

There are many different software products that compute Bayesian analyses, including JAGS, WinBUGS/OpenBUGS, Stan, PyMC3, Pyro, dozens of R packages (https://cran.r-project.org/view=Bayesian), JASP, Jamovi, Minitab, SAS/JMP, Stata, SPSS, Mplus and others. Software and features are continually evolving. For a list of statistical software, some of which may have Bayesian options, consider https://en.wikipedia.org/wiki/List_of_statistical_software. It is up to the user to understand the specific computations and output of whatever software is being used.

Most contemporary Bayesian analyses are accomplished using MCMC^15,58,59^. MCMC creates a representation of a probability distribution over parameters by taking a random walk through parameter space, tending to walk in the high-probability regions of the space and only occasionally walking in the low-probability regions. After a long walk, the footsteps provide a high-resolution representation of the underlying mathematical posterior distribution. There is nothing inherently Bayesian in MCMC; MCMC merely provides a high-resolution pixelated representation of the posterior distribution.

The MCMC random walk, called a chain, must have explored the parameter space sufficiently to be genuinely representative of the posterior distribution. There are two key aspects of the chain that the user must check. First, there should be evidence that the chains did not get stuck in some unrepresentative region of the parameter space but instead converged on a representative walk. A popular method for checking convergence is to run at least three separate chains and check that all the chains overlap each other, as measured by the potential scale reduction factor (PSRF)^60^, also called R-hat ($\hat R$). “… [T]herethere are several other commonly implemented convergence diagnostics in programs such as R; for example, the Geweke diagnostic^61^, the Heidelberger and Welch diagnostic^62^, and the Raftery and Lewis diagnostic^63,64^ for determining the length of the burn-in and post-burn-in portions of the chain”^7^. Regardless of which statistic is used, the goal is to demonstrate reasonable assurance that the MCMC chains are not stuck.

The second key aspect of the MCMC chain to check is the stability of its estimates. Even if chains have converged, they might not be long enough to smoothly and accurately represent the distribution with high resolution. A key indicator is the ESS, which is the effective number of steps in the MCMC chain after the clumpiness of autocorrelation is factored out. The ESS has nothing to do with the number of points in the data, which is fixed. A less confusing name for ESS might be ‘effective MCMC length’, but this term is never used. Sufficient ESS is essential for having stable parameter estimates^65^, in particular for stable limits of credible intervals. Recommendations for specific best practices may differ among experts depending on the application. For reasonably stable estimates of limits of highest-density intervals (HDIs), I recommend^15^ that ESS ≥ 10,000. For stable estimates of limits of equal-tailed intervals, ESS can be lower. The central tendency can be stably estimated with smaller ESS (when the central tendency is in a high-density region of the distribution)^66^.

Every parameter and derived value has a distinct PSRF and ESS. Therefore, the convergence and resolution values of every parameter should be reported. The main text could summarize these, with details being provided in the supplementary materials.

When computing BFs for model comparison or hypothesis testing, different computational procedures might be used. It is up to the user to know what method is being used by their software. When MCMC underlies the computation, it is again relevant to report the convergence and stability of the chain. For instance, the example in the Supplementary Information uses bridge sampling^55,56^ to compute BFs, and reports an estimate of approximation error.

Some Bayesian software uses methods other than MCMC to compute posterior distributions, such as integrated nested Laplace approximation^67^ or variational inference^68^. In these cases, it is not relevant to report MCMC convergence statistics. However, if the method is not an exact calculation of the posterior (as it would be when using a fully conjugate prior, for instance), there will be approximations inherent in the result, and any such approximations should be noted.

Reporting points are listed in Table 1.

## Describing the posterior distribution

Statistical models can only be meaningful if they mimic the data in some relevant way. Therefore a prerequisite for reporting the parameter estimates is demonstrating that the model does indeed usefully describe the data. This prerequisite applies to frequentist and Bayesian methods. In the Bayesian framework, showing that the model mimics the data is called a posterior predictive check^69–71^. The predictions of the model, using parameter values from the posterior distribution, are compared with the actual data. There is no universally best way to perform a posterior predictive check, because different applications can have very different data structures and different aspects of data might be of different theoretical importance. A posterior predictive check is usually qualitative (for example, graphical), but quantitative posterior predictive checks are possible and could involve formal model selection. In any case, a posterior predictive check should show that the important trends in the data are usefully captured by the model. After the posterior predictive check, details of the parameters can be reported.

The marginal posterior distribution of a parameter or derived measure can usually be summarized adequately in words. In the vast majority of cases, the marginal posterior distribution of a parameter is unimodal and only modestly skewed, and that form can be summarized in words by reporting the central tendency and limits of a credible interval. A conventional probability mass for the credible interval is 95%, but in any case the chosen credible interval probability mass must be clearly reported. The central tendency and credible interval can be based on probability density, in which case the report specifies the mode and 95% HDI. Alternatively, the central tendency and credible interval can be based on quantiles (cumulative probability), in which case the report specifies the median and 95% ETI. Values based on densities or on quantiles will usually be quantitatively similar except in highly skewed distributions or in multimodal distributions with deep troughs^15,72^. In any case, the report must clearly indicate which form (density or quantile) is being used.

Graphical representations of unimodal marginal distributions are usually redundant with the text description. Moreover, many applications involve tens or hundreds of parameters and derived variables, and including graphs of them all could be counterproductive and superfluous. Online supplementary material usually has less constraint on length than the main text of publications, and can therefore include more graphical representations. Graphical representations can be especially useful to explain unusual distributions—for example, when truncated priors produce truncated posteriors.

When doing model comparison or hypothesis tests, it is conventional to report the BF for pairs of models. The BF is the coefficient that converts the prior probabilities of the models to the posterior probabilities of the models, and the magnitude of the BF indicates the degree to which model probabilities are shifted from their prior probabilities^47,73^. A formal definition of the BF is provided in the Supplementary Information. The BF alone does not indicate the posterior model probabilities. Nevertheless, posterior probabilities are the ultimate output of Bayesian inference and are essential. Despite the ultimate interest in posterior model probabilities, they are often not reported because analysts demur at committing to specific prior model probabilities.

**Fig. 1:**
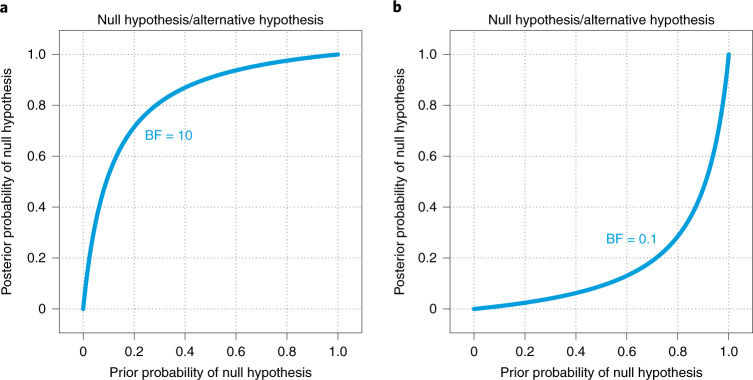
Posterior model probability as a function of prior model probability. **a**, BF = 10. **b**, BF = 0.1.

To satisfy both the need for reporting the BF and the need for posterior model probabilities, I recommend that analysts report the BF and posterior model probabilities for a range of prior model probabilities. Figure 1 plots the posterior probability of a model as a function of its prior probability for specific values of the BF. The plots are a variation of the leaf plot^74^ for interpreting diagnostic tests. In Fig. 1, I call the models null and alternative hypotheses. The titles of the panels show the null hypothesis as the numerator of the BF and the alternative hypothesis as the denominator. If we suppose the BF for the null hypothesis relative to the alternative hypothesis is 10, probabilities are shifted towards the null hypothesis, as shown in Fig. 1a. The curve shows the posterior probability of the null hypothesis as a function of its prior probability. For example, if the prior probability of the null hypothesis is 0.05, then its posterior probability is 0.34; if the prior probability of the null hypothesis is 0.5, then its posterior probability is 0.91; and if the prior probability of the null hypothesis is 0.95, then its posterior probability is 0.99. Figure 1b shows the case in which BF = 0.1, which shifts the model probabilities away from the null hypothesis. In this case, if the prior probability of the null hypothesis is 0.05, then its posterior probability is 0.01; if the prior probability of the null hypothesis is 0.5, then its posterior probability is 0.09; and if the prior probability of the null hypothesis is 0.95, then its posterior probability is 0.66. This graphical device is offered as one way to concisely convey information about both the BF and the posterior model probability simultaneously, without committing to a specific prior model probability. This is discussed further in Supplementary Information. The graph does not have to be included in reports, but it becomes even more useful in the context of making decisions about models, as elaborated in BARG step 4.

Reporting points are listed in Table 1.

## Reporting any decisions

The Bayesian inference per se produces the posterior distribution that was emphasized in step 3. Some analysts use the posterior distribution to make a decision about specific parameter values, hypotheses or models. Such a decision is an additional consideration that involves establishing thresholds for each decision. This is exactly analogous to frequentist decision making, in which a *p* value for a particular test statistic is computed from the data and sampling intentions, but deciding whether the *p* value is significant requires specifying a threshold value such as 0.05.

It is important to distinguish (1) the posterior distribution produced by the Bayesian inference from (2) the decision rule and its thresholds, because the posterior distribution depends only on the data and prior distribution, whereas the decision depends on separate considerations such as decision thresholds and the costs and benefits of errors and correct decisions^75^. There can be agreement among researchers that a particular posterior distribution is richly informative and robust, but disagreement regarding which decision procedure or threshold to use. Again, this is analogous to frequentist decision making, in which researchers might disagree about the significance threshold for making decisions^76^ or disagree about which test statistic to use (for example, a likelihood-ratio test of models or a direct test of parameter null value within a model). Importantly, if the Bayesian posterior distribution is thoroughly and reproducibly reported, readers who prefer different decision procedures can make their own decisions.

A full-blown decision-theoretic approach would require specification of utilities for each possible correct or erroneous decision, and specification of a loss function that is minimized by the decision rule^75,77,78^. Such utilities are rarely specified in behavioural research. If, however, utilities and a loss function are used, they should be reported and explained in full.

As was emphasized in the preamble of the BARG, it should be considered whether decisions are needed at all, or if a declaration of significance is merely compliance with ritual^42,43,79^. Whichever decision procedure is used, it should be kept in mind that the decision thresholds are often merely conventional, and what counts as a negligible effect size should be considered, not merely whether the sample size of the data was large enough to ‘significantly’ favour one hypothesis or the other with a tiny effect^80,81^.

Some analysts find it useful to base decisions about null values by considering where the posterior distribution falls relative to the null value. With these decision rules, when the bulk of the posterior distribution falls sufficiently close to the null value, then the null value is ‘accepted’ and when the bulk of the posterior distribution falls sufficiently far away from the null value, then the null value is ‘rejected’. The threshold for being sufficiently close or far away goes by different names, but I will refer to it as the region of practical equivalence (ROPE) to the null value. The ROPE limits are established by practical and theoretical considerations before seeing the results^81^. Some decision rules consider only the proportion of the posterior distribution that falls within the ROPE^82^. Other decision rules consider the relationship of the credible interval to the ROPE^81,83^. If this type of decision rule is used, it is important to report the ROPE and its justification.

Some analysts find it useful to base decisions about null values by formalizing the null value as a distinct model, called the null hypothesis, and doing Bayesian model comparison of the null hypothesis and an alternative hypothesis. This approach posits distinct prior probability mass at (or in a narrow region near) the null value. As explained in step 3, the BF quantifies the shift in model probabilities away from the prior model probabilities. Basing a decision on the BF alone is tantamount to assuming that the prior probability of the null hypothesis is fixed at 0.50. For example, in disease diagnosis, the BF of a diagnostic test result can be derived directly from the rates of false negatives and false positives, and basing diagnostic decisions on the BF ignores the base rate of the disease and instead assumes that the base rate of the disease is 50%. As another example, testing the existence of extrasensory perception (the ‘psi effect’), using the BF assumes that the prior probability of the psi effect is 50%, but “it is appropriate to hold very low prior odds of a psi effect, and appropriate [prior] odds may be as extreme as millions, billions, or even higher against psi. Against such [prior] odds, a BF of even 330 to 1 seems small and inconsequential in practical terms”^84^. Therefore, I recommend that reports include not only the BF, but also what prior model probability would be needed for the posterior model probability to exceed a decision threshold, as explained next.

**Fig. 2:**
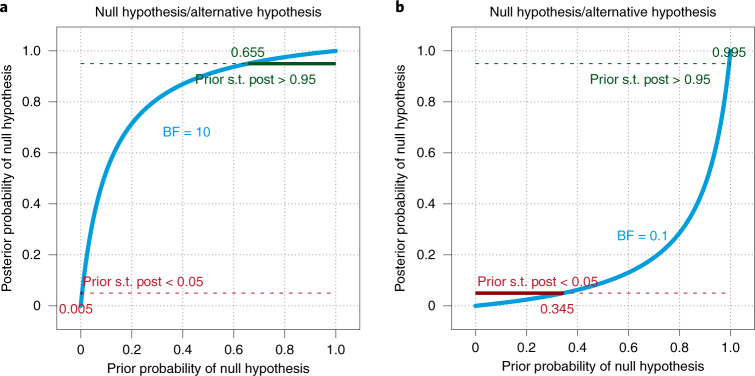
Decision threshold for posterior model probability with corresponding prior model probability. **a**,**b**, Decision thresholds on posterior model probabilities are plotted as dashed horizontal lines, with BF values of 10 (**a**) and 0.1 (**b**). Where the posterior probability exceeds the decision threshold, the decision threshold lines are marked by solid segments and annotated with the corresponding prior model probability. ‘Prior s.t. post > 0.95’ means the prior model probability such that the posterior model probability is greater than 0.95. The two panels show different BFs.

Figure 1 shows the BF curve that plots posterior model probability as a function of prior model probability. The curve can be marked at a criterion posterior model probability for accepting or rejecting to make visually explicit the range of prior model probabilities that result in exceeding the decision criterion. Examples are shown in Fig. 2, using a criterion posterior probability of 0.95, which means the winning model is at least 19 times more probable than the losing model. In Fig. 2a, with BF = 10, the annotated horizontal line near the top indicates that to decide to ‘accept’ the null hypothesis with posterior probability of at least 0.95, the prior probability of the null hypothesis must be at least 0.655. In Fig. 2b, with BF = 0.1, the annotated horizontal line near the bottom indicates that to decide to ‘reject’ the null hypothesis with posterior probability no greater than 0.05, the prior probability of the null hypothesis must be no greater than 0.345. These graphical displays are offered as useful visualizations to relate BFs to posterior model probabilities and decision thresholds. The essential information to report is the BF, the criterion posterior model probability for accept (or reject), and the minimum (or maximum) prior model probability needed to exceed that decision threshold. To be clear, this recommendation is unique to the BARG, but would be very useful to help researchers understand the implications of BFs. A Bayesian treatment of uncertain prior model probabilities is provided in ref. ^85^.

If deciding about null values, the decision about the null value should never be reported without reporting the estimate of the parameter value (that is, its central tendency and credible interval). This is crucial to forestall fallacies of black-and-white thinking and to facilitate meta-analysis^31,43,86^.

Typically, reports of decisions are integrated with reports of descriptions. Nevertheless, the two topics are separated as distinct steps in the BARG to emphasize that they are separate issues. The reporting points apply even if reports of decisions are interspersed among the descriptions. Care should be taken in the report to distinguish the summary of the posterior distribution from the decision procedure. Reporting points are listed in Table 1.

## Reporting sensitivity analysis

The researcher should always conduct an analysis of how sensitive the posterior distribution is to the choice of prior distribution. Different types of applications tend to have different sensitivities to the prior. Applications with small datasets tend to be more sensitive to the prior than applications with large datasets. In continuous parameter estimation with broad priors, the posterior is typically not very sensitive to the choice of prior, and sensitivity analyses can be minimal if the prior is truly broad relative to the posterior (though unanticipated sensitivity can arise; for example, on higher-level covariance priors^7^). By contrast, when using a strongly informed prior it is important to demonstrate the results from other priors. And when doing model selection or hypothesis testing, the BF can sometimes be very sensitive to the choice of prior within models, so a sensitivity analysis is crucial.

A prior specification may involve numerous constants that specify the location, scale, covariance and shape (for example, skew or kurtosis) of the prior distribution on the parameters. Note that there can be many more prior constants than there are estimated parameters; for example, a normal prior on a single parameter involves two constants (the mean and standard deviation). Some models may involve tens or hundreds of prior constants. Because of combinatorial explosion (for example, *C* prior constants, each with *L* candidate values, result in *L*^*C*^ combinations), it is unrealistic to factorially manipulate all the prior constants and assess their impact on the posterior distribution. For instance, the example in the Supplementary Information involves a model with 6 parameters and *C* = 27 prior constants. Therefore, the analyst must selectively consider changes to the prior that are theoretically relevant or that address the concerns of a reasonable sceptic.

If using representative data to inform all models, then it may be useful to try different sets of representative data. The example in the Supplementary Information shows results using three priors: (1) a generic broad prior, (2) a prior informed by comparable data other than the target data, and (3) a prior informed by a representative subsample of the target data.

If using default priors, it may be useful to vary the uncertainty of the prior on the effect size. For example, Wagenmakers, Verhagen and Ly^87^ considered BFs for a Pearson correlation parameter, *ρ*. The null hypothesis set *ρ* ≡ 0, and the sensitivity analysis considered priors for the alternative hypothesis that remained centred on *ρ* = 0 but varied from flat to strongly peaked. This family of variation might not accurately capture empirically informed priors, which involve priors centred at observed effect sizes not at zero. An example using empirically informed priors was also provided in what they called a ‘replication’ BF^88^ that used the entire dataset from a previous study (which can create a fairly narrow alternative prior), rather than a small representative dataset (which yields a broader alternative prior that nevertheless captures the trends) as in the example in the Supplementary Information.

Assessing differences between posterior distributions from different priors is itself a complex issue. The posterior distribution is a joint distribution on a multidimensional parameter space. It is conceivable that parameter correlations may change while their marginal distributions remain relatively unchanged. Typically, however, analysts are concerned primarily with the individual parameter marginal distributions. Two useful ways to visually compare distributions are superimposed density curves and superimposed cumulative-distribution curves. Density curves are especially useful for visually highlighting the modes and HDIs, whereas cumulative-distribution curves are especially useful for visually highlighting medians and ETIs. Density curves are inherently distorted by smoothing kernels, whereas cumulative-distribution curves are limited only by the pixel resolution of the display. The example in the Supplementary Information provides illustrations. It can be wasteful of page space to make graphs for every parameter in a model, especially in models with tens or hundreds of parameters. Whether or not visual graphs are displayed, it is important to include numerical tables showing the central tendency and credible interval for the marginal posterior distribution of every parameter or relevant derived measure for every prior, either in the main text or in appendices or supplementary material.

When assaying sensitivity of BFs to choice of prior, it is conceivable to superimpose several BF curves (as in Fig. 2), but this would be cluttered and difficult to read. Therefore, BFs from different priors may be best presented in tabular format along with the minimum prior probabilities required to achieve decisive posterior probability.

Procedures for conducting a convincing sensitivity analysis may depend strongly on the specific model and data, and such procedures are still being developed^89–93^. The choice of other priors to compare is crucial, yet can be controversial. In applications for which the duration of each individual MCMC is long, an exhaustive sensitivity analysis may take a very long time, and efficiencies may need to be introduced. Therefore the guidelines here are general, and the analyst is encouraged to explore the literature for model-specific recommendations. Ultimately, the analyst must be thoughtful in exploring plausibly interesting variations in the prior and be forthright in presenting the results. Because of the potential length of a thorough presentation, online supplementary material may be needed and is encouraged (see ‘Make it reproducible’ (BARG step 6)). Reporting points are listed in Table 1.

## Making it reproducible

For an analysis to be reproducible, it should be thoroughly and transparently explained in the first place, and the preceding points have been designed with this goal in mind. A final and important part of reproducibility is making the computer code and data easily available to others by posting them at a public and persistent website. The data, code and other files should be findable, accessible, interoperable and reusable (the FAIR principles^94,95^). Of the several essential points listed in Table 1, I highlight two: first, make the computer code readable by human beings. Annotate the computer code with explanatory comments, and arrange the code with ample spaces between terms and indented breaks across lines so that a human being can visually parse the syntax. Second, check that the computer code runs as posted when it is downloaded to another computer. This helps to verify that all necessary files and directories are set for use by third parties.

## Encouraging use of guidelines

Despite the existence of previous guidelines for reporting research, guidelines are rarely mentioned in reports and are probably rarely consulted. If the BARG are to be useful, they must be used. Researchers have argued that reporting guidelines may have benefits and should be endorsed by journals across many fields^9,96–101^. It has been observed that “Journals promoting [guidelines] were both key motivators and awareness mechanisms; peers and educational workshops were also important influencing factors to a lesser degree”^102^. The recommendations of the International Committee of International Journal Editors^103^ state that “Journals are encouraged to ask authors to follow … guidelines because they help authors describe the study in enough detail for it to be evaluated by editors, reviewers, readers, and other researchers …”.

When promoting guidelines such as the BARG, we must avoid promoting mindless statistical rituals that perversely encourage questionable research practices^41,42^ and embracing a culture of obedient compliance that shames individual practitioners^79^. The BARG avoid mindless compliance with ritualized norms by encouraging reflective application of essentials to produce thoughtful, thorough, transparent and reproducible Bayesian analyses.

The BARG have assimilated many previous checklists and guidelines, but also include additional points, organization, explanation and an extended example (Supplementary Information). If researchers, authors, editors, reviewers, educators and students thoughtfully follow the BARG, statistical analyses may be better in quality, transparency, impact and reproducibility. Statistical methods and practices are continually evolving, but the key points emphasized by the BARG should be applicable for years to come.

## Supplementary information

Supplementary Information Example of applying the reporting guidelines and review of previous reporting guidelines.
