# Supplementary material for: Bayesian Analysis Reporting Guidelines
Source: Nat Hum Behav. 2021 Aug 16;5(10):1282–91. doi: 10.1038/s41562-021-01177-7 (PMC8526359; doi:10.1038/s41562-021-01177-7)
Supplement: Supplementary file 1 — Example of applying the reporting guidelines and review of previous reporting guidelines. [file 41562_2021_1177_MOESM1_ESM.pdf]

nature portfolio

https://doi.org/10.1038/s41562-021-01177-7

Supplementary information

Bayesian Analysis Reporting Guidelines

In the format provided by the authors and unedited

Code Code ▾
Code
Code

This is Supplementary Information (SI) to the article, “Bayesian analysis reporting guidelines” (BARG; Kruschke, J. K., *Nature Human Behaviour*, 2021). This SI has two main sections:

- First, there is an example of a detailed Bayesian analysis with full reporting according to the BARG. The example of applying the BARG begins in the next section. A subsection of that that example, headed “Outline…”, provides additional details regarding the organization of its components.
- Second, there is a review of previous Bayesian reporting guidelines. The review of previous Bayesian reporting guidelines appears after the example at the end of this document, in the final section.

This SI is best viewed onscreen from the HTML format available at https://osf.io/w7cph/ (https://osf.io/w7cph/). The HTML version of this document has a floating and dynamic table of contents visible in the left margin on wide screens. The table of contents is intended to aid navigation through the various subsections. The HTML version also has clickable buttons on the right side which dynamically unfold R code chunks if the reader wishes to examine them. The buttons can be clicked again to hide the code chunks. The HTML version also has tables that can be dynamically re-sorted by different columns.

1 Example of applying the BARG

This example applies the Bayesian analysis reporting guidelines (BARG) to ordinal data, specifically the ratings of movies from Liddell and Kruschke (2018)[1] and its supplemental material at https://osf.io/53ce9/ (https://osf.io/53ce9/).

The primary goal of this document is to illustrate thorough reporting of the process and results of a Bayesian analysis. The goal is *not* to explain details of the specific computer programming I happened to use. Therefore most computer code is folded out of sight but can be revealed in the HTML document by clicking the “Code” buttons throughout the document. Moreover, this document is *not* intended to be a tutorial on Bayesian methods, and it is assumed that the reader is familiar with the basic concepts of Bayesian analysis (for introductions, see, e.g., references 2; 3; 4; 5; 6; 7).

The analysis begins with setting up the R software environment; various packages are loaded.

Code

Next, various utility functions are defined. The functions defined here are idiosyncratic and do not need to be studied to understand the results presented later. (Some functions defined in this section might be vestigial and ultimately not used later.)

Code

Readers might be interested in a specific function I created for plotting Bayes factors (BF’s), results from which appeared in the article that describes the BARG. The function takes the BF as input (along with other inputs) and creates a plot of posterior model probability as a function of prior model probability. The plot also shows the range of prior probabilities that yield posterior probabilities beyond a decision threshold. Examples of the plots appear near the end of this document, in the section on hypothesis testing. The function, called `bfplot()`, is defined in the following code chunk.

Code

2 The Data

(Cf. BARG Step 1.A.)

The data are ratings of movies from Amazon.com, on an ordinal scale from 1 to 5 stars. For details of data acquisition see Ref 1. The original full data set contains ratings of 36 movies. For purposes of the present illustration, two of the movies are selected for comparison. The cases are selected to demonstrate that the conventional assumption of homogeneous variances can be very misleading, which was one of the main points of Ref 1.

Code

The data, displayed numerically: In the frequency table below, there is one row per movie, with the rows labeled by arbitrary ID numbers (9 , 15) subsequently generically called ‘Case 1’ and ‘Case 2’. There is one column per star-rating level with the columns labeled arbitrarily as n1, n2, n3, n4, n5. Each cell contains the count (frequency) of ratings.

Code

```
##      n1 n2 n3  n4  n5
## 9   110 63 52  89 209
## 15   71 43 92 147 190
```

The data, displayed graphically:

Code

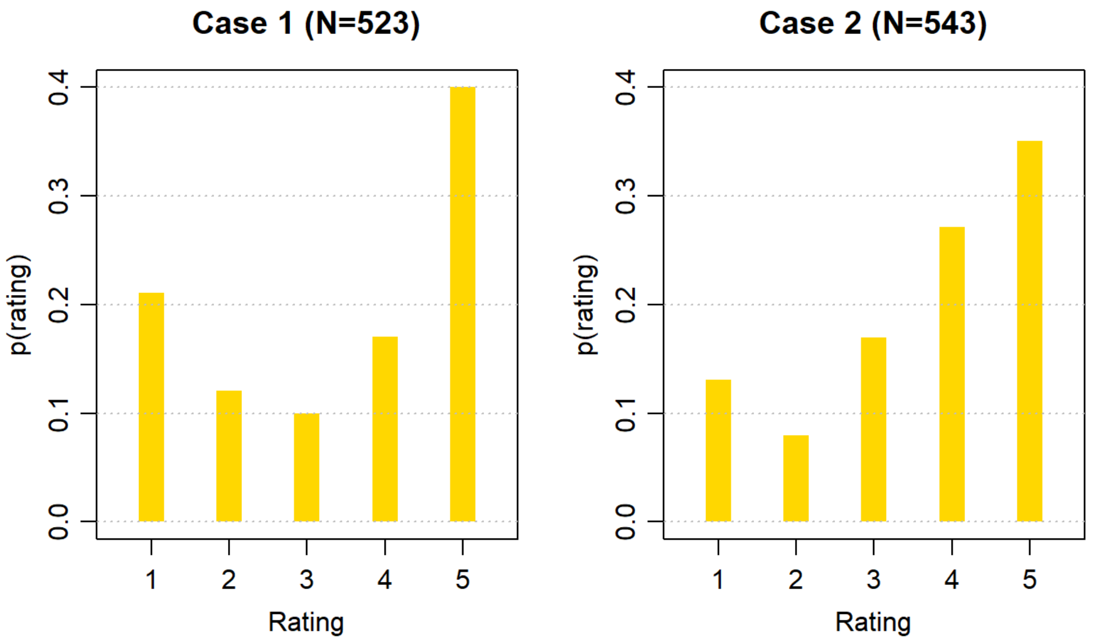

Visual inspection of the graphs and table shows that the distribution of ratings is “J” shaped, which is not unusual for rating distributions.[8] Moreover, while Case 1 has a higher proportion of 5-star ratings than Case 2, Case 1 also has a higher proportion of 1-star ratings than Case 2.

3 Goals of the analysis

(Cf. BARG Preamble B.)

I am interested in (i) the differences of the central tendencies of the cases, and (ii) the differences of the standard deviations of the cases. First, the magnitude of difference between central tendencies indicates how much better is one case than the other, on average. The difference of central tendencies will be converted to effect size (Cohen’s *d*), which is the difference relative to the average standard deviation. The effect size indicates the magnitude of difference between movies relative to the variability of opinion within movies. Second, the standard deviation indicates how much opinion varies across people, and the magnitude of difference between standard deviations indicates how much one case has more variance of opinion than the other. In other words, some movies elicit consistent opinions while other movies elicit diverse opinions. The model, explained later, describes means and standard deviations on a *latent metric scale, not on the observed scale of ordinal labels*.

I will also report decisions regarding whether the difference of means and difference of variances (on the latent scale) are effectively zero or non-zero. Decisions will be made two ways. First, decisions will be made by using the posterior estimated magnitudes of means and variances. The analysis for estimating parameter values and assessing null values takes approximately the first third of

the example text.

A second way for making decisions is model comparison for null hypothesis testing. The full model has a distinct mean and variance for each case, whereas one restricted model requires the same variance for all cases (but allows different means), and a second restricted model requires the same mean for all cases (but allows different variances). The analysis of model comparison for null hypothesis testing takes approximately the latter two-thirds of the example text.

A note on terminology: A “null *value*” is the value of a parameter that indicates a null effect, such as an effect size of zero. By contrast, a “null *hypothesis*” is an entire model that has the relevant parameter fixed at its null value. An analyst can “*assess a null value*” by estimating the parameter value and considering the relation of its posterior distribution to its null value. An analyst can “*test a null hypothesis*” by comparing a full model with a restricted model that fixes the parameter at the null value.

4 The Model

(Cf. BARG Step 1.B.)

The data are ordinal values, which I choose to describe with an *ordered-probit model*. It is *not* appropriate to treat the data as if they were metric, a.k.a. interval, values. (In fact, treating the data as normally-distributed metric values and applying a t-test yields the inappropriate and misleading conclusion that the two movies have significantly different mean ratings.) There is no claim that an ordered-probit model is the correct model of the data or even the best model of the data. Rather, the ordered-probit model is better than treating the data as if they were normally distributed metric values. Moreover, the ordered-probit model fits the rating distributions reasonably well (as shown later by posterior predictive checks).

The ordered-probit model is explained in more detail below. For extensive background information about ordered-probit models and their analysis in Bayesian software, see Ref 1 and its supplemental material at https://osf.io/53ce9/ (https://osf.io/53ce9/).

Another resource for Bayesian analysis of ordinal data was provided by Ref 9, but unfortunately the `brms` software featured in that article does not (at the time of this writing) have a multivariate joint prior on all the parameters as explained below. Also, in the present application the JAGS software used here is much faster than the Stan software[10] used by `brms` .

(Cf. BARG Preamble A.)

The Bayesian approach is especially useful for this application because of its flexibility for specifying exactly the desired model structure. Moreover, the Bayesian approach directly yields credible intervals for every parameter and derived variable, and yields posterior model probabilities for null and alternative models.

4.1 Likelihood function

(Cf. BARG Step 1.D.)

In an ordered-probit model, there is a latent continuous variable underlying the ordinal response. The population is assumed to be a normally distributed on the latent variable, with mean μ_i (for case i) and standard deviation σ_i. The latent variable is cut at thresholds, θ_1 to θ_{K-1} (for K response levels), such that latent values between θ_{k-1} and θ_k produce ordinal response j. The figure below illustrates the ordered-probit model:

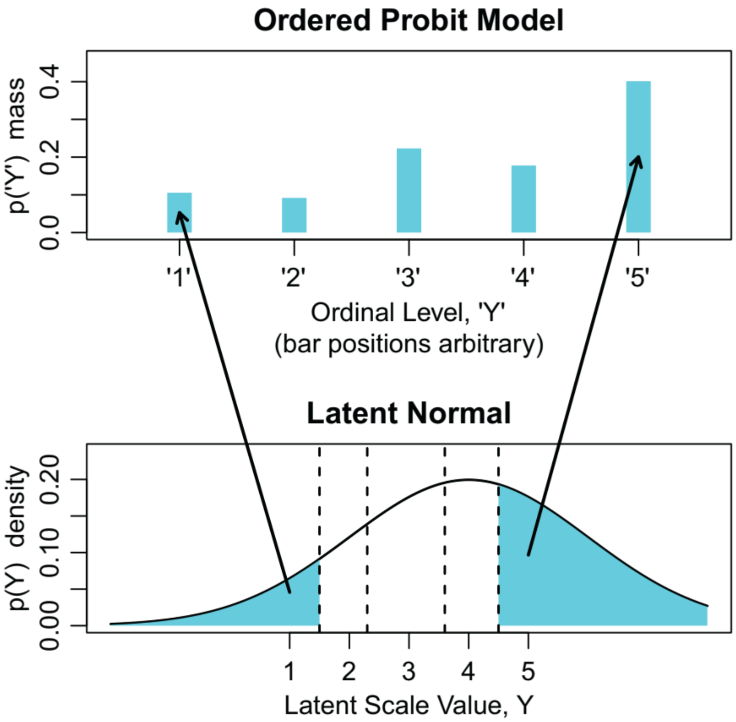

Figure: Ordinal data in upper panel are produced from thresholded cumulative-normal in lower panel. (Diagram is from Figure 1 of Liddell and Kruschke (2018)[1], p. 329, used with permission.)

Mathematically, the probability of response level k is

$$p\big(k \,|\, \mu_i, \sigma_i, \{\theta_j\}\big) = \Phi\big((\theta_k - \mu_i)/\sigma_i\big) - \Phi\big((\theta_{k-1} - \mu_i)/\sigma_i\big)$$

where $\Phi()$ is the standardized cumulative-normal function. For the highest response level K the threshold θ_K is effectively $+\infty$, and for the lowest response level $k = 1$ the threshold θ_{k-1} is effectively $-\infty$. The thresholds are assumed to be determined by the response process and are therefore the same across all cases i (because all cases are measured by the same response process).

Parameters: The parameters consist of

- μ_i and σ_i for each case i
- the thresholds θ_1 to θ_{K-1}.

However, the "stretch" and position of the latent scale are arbitrary —by analogy, the latent scale could be Fahrenheit or Celsius— and therefore *two parameter values are fixed at arbitrary constants*. In the traditional parameterization for an ordered-probit model, $\mu_1 \equiv 0.0$ and $\sigma_1 \equiv 1.0$ and all other parameters are specified relative to those constants. I find that parameterization to be unintuitive,[1,3] and prefer instead to fix $\theta_1 \equiv 1.5$ and $\theta_{K-1} \equiv K + 0.5$ (where K is the highest ordinal level), *which makes the values of the parameters correspond roughly to the response scale of* 1 *through* K. Thus, in the present application with two cases (i.e., two movies) and five response levels, *there are a total of six estimated parameters:* μ_1, σ_1, μ_2, σ_2, θ_2, *and* θ_3, with $\theta_1 \equiv 1.5$ and $\theta_4 \equiv 4.5$.

The means (μ_1 and μ_2) describe the central tendency of ratings on the latent scale, and the standard deviations (σ_1 and σ_2) describe the variability of ratings across people on the latent scale. *Primary interest is in the magnitudes of the means and standard deviations, and in the magnitudes of the differences of means and standard deviations across cases.*

In the threshold-pinned parameterization that has $\theta_1 \equiv 1.5$ and $\theta_4 \equiv 4.5$, a mean of 1.5 indicates that 50% of the responses will be level "1" and 50% of the responses will be levels $>$"1". A mean of 4.5 indicates that 50% of the responses will be level "5". A mean of 3.0 suggests the underlying (latent) rating is near a "3" on the response scale, subject to exact placement of the thresholds. A difference between means of 1.0 suggests the underlying (latent) ratings have central tendencies roughly 1 response level apart.

4.2 Prior

(Cf. BARG Step 1.C. and D.)

For basic parameter estimation from a broad prior, the prior can simply use diffuse univariate distributions on each parameter, as was done by Ref 1 and for which software was provided in the supplementary material at https://osf.io/53ce9/ (https://osf.io/53ce9/).

The present application will also illustrate model comparison with Bayes factors. Bayes factors can be very sensitive to the priors, and therefore it is crucial to specify meaningful priors that reasonably mimic parameter distributions that could have arisen from realistic data (or from idealized data generated from theory). It turns out that posterior distributions from the ordered-probit model can have strongly correlated parameters, and therefore these correlations should be represented in the prior distributions that are used for Bayes factors.

The threshold parameters are typically strongly correlated with each other, in any application of an ordered-probit model. The correlation of thresholds is natural because if one threshold estimate is scooted higher then the probabilities of intervals can be roughly maintained by also scooting the other threshold estimates a bit higher. Moreover, the μ and σ parameters can be strongly correlated. The correlation is especially strong for μ values near or beyond end thresholds, as is typical for movie ratings. For example, when there are a lot of 5-star ratings, then μ is estimated to be near or above the highest threshold, and the data can be reasonably well fit by scooting μ to a higher value while compensating by making σ also a higher value. The μ and σ parameters can also be correlated with the threshold parameters. Examples will be shown later.

I have chosen to represent these correlations by using a multivariate normal distribution as the joint prior for all parameters simultaneously. A multivariate normal is specified by the mean of each variable (i.e., a vector of means) and the covariance matrix of the variables. The mean of the multivariate normal indicates the central tendency of the parameter value, and *the variances represent the uncertainty of the prior* such that larger variances represent greater uncertainty. The covariances represents how strongly the parameters are thought to covary.

Because each variable of a multivariate normal spans the real values from $-\infty$ to $+\infty$ but a standard-deviation parameter (σ_j) can only be non-negative, *the multivariate normal is a prior on the logarithm of the standard deviation,* $\log(\sigma_j)$. Formally, the prior is given by

$$\begin{bmatrix} \mu_1 \\ \mu_2 \\ \log(\sigma_1) \\ \log(\sigma_2) \\ \theta_2 \\ \theta_3 \end{bmatrix} \sim \text{mvnorm}\left(\begin{bmatrix} M_{\mu_1} \\ M_{\mu_2} \\ M_{\log(\sigma_1)} \\ M_{\log(\sigma_2)} \\ M_{\theta_2} \\ M_{\theta_3} \end{bmatrix}, \begin{bmatrix} \text{V}_{\mu_1,\mu_1} & \text{C}_{\mu_1,\mu_2} & \text{C}_{\mu_1,\log(\sigma_1)} & \text{C}_{\mu_1,\log(\sigma_2)} & \text{C}_{\mu_1,\theta_2} & \text{C}_{\mu_1,\theta_3} \\ \text{C}_{\mu_2,\mu_1} & \text{V}_{\mu_2,\mu_2} & \text{C}_{\mu_2,\log(\sigma_1)} & \text{C}_{\mu_2,\log(\sigma_2)} & \text{C}_{\mu_2,\theta_2} & \text{C}_{\mu_2,\theta_3} \\ \text{C}_{\log(\sigma_1),\mu_1} & \text{C}_{\log(\sigma_1),\mu_2} & \text{V}_{\log(\sigma_1),\log(\sigma_1)} & \text{C}_{\log(\sigma_1),\log(\sigma_2)} & \text{C}_{\log(\sigma_1),\theta_2} & \text{C}_{\log(\sigma_1),\theta_3} \\ \text{C}_{\log(\sigma_2),\mu_1} & \text{C}_{\log(\sigma_2),\mu_2} & \text{C}_{\log(\sigma_2),\log(\sigma_1)} & \text{V}_{\log(\sigma_2),\log(\sigma_2)} & \text{C}_{\log(\sigma_2),\theta_2} & \text{C}_{\log(\sigma_2),\theta_3} \\ \text{C}_{\theta_2,\mu_1} & \text{C}_{\theta_2,\mu_2} & \text{C}_{\theta_2,\log(\sigma_1)} & \text{C}_{\theta_2,\log(\sigma_2)} & \text{V}_{\theta_2,\theta_2} & \text{C}_{\theta_2,\theta_3} \\ \text{C}_{\theta_3,\mu_1} & \text{C}_{\theta_3,\mu_2} & \text{C}_{\theta_3,\log(\sigma_1)} & \text{C}_{\theta_3,\log(\sigma_2)} & \text{C}_{\theta_3,\theta_2} & \text{V}_{\theta_3,\theta_3} \end{bmatrix} \right)$$

where mvnorm denotes the multivariate normal distribution, M is the prior mean, V is the prior variance (uncertainty), and C is the prior covariance. The covariance matrix is symmetric around the main diagonal (i.e., $C_{i,j} = C_{j,i}$). The formula above specifies only thresholds 2 and 3 because those are the only estimated thresholds in this application; thresholds 1 and 4 are pinned at fixed values as explained earlier. In summary, the prior multivariate normal distribution is specified by 27 constants: 6 means, 6 variances, and 15 distinct covariances.

Three choices for prior constants. The choice of M, V, and C constants will be explained in detail below, the first time each specific prior is introduced in an analysis. There will be three versions of the prior, to explore the sensitivity of the posterior to the choice of prior.

- One choice of prior will set the M, V, and C constants to values that make the prior *broad* and symmetrically span a very wide range on the ordinal response space.
- A second choice of prior will be mildly informed by representative data from *other* movies, with both target movies informed the same.
- A third choice of prior will be mildly informed by a representative *subset* of the data from the target movies, with each movie informed by its own tendency. This type of mildly informed prior is especially useful for computing Bayes factors in hypothesis testing.

4.3 Restricted models

Two restricted models will be fit for comparison with the full model defined above.

- One restricted model uses a single mean for all cases, but distinct standard deviations. Its structure is the same as the full model except $\mu_1 \equiv \mu_2$. The model therefore has 5 distinct parameters (μ, σ_1, σ_2, θ_2, θ_3) and 20 constants in its multivariate normal prior.
- The other restricted model uses a single variance for all cases, but distinct means. Its structure is the same as the full model except $\sigma_1 \equiv \sigma_2$. The model therefore has 5 distinct parameters (μ_1, μ_2, σ, θ_2, θ_3) and 20 constants in its multivariate normal prior.

4.4 R code for models

(Cf. BARG Step 2.A.)

The Bayesian analysis was computed in R using JAGS; version details are provided at the end of the document in the section on How to Reproduce this Document. The present section shows the R code for the models. This section is not intended to be studied on a first reading, but is included at this point for reference. In particular, interested readers can check that the R code correctly implements the models that were specified mathematically above. (Cf. BARG Step 1.D.)

The functions set initial values for the MCMC chains, so they will generate reproducible outputs (cf. BARG Step 6.H.).

R code for full model. The function takes three types of inputs: (i) data, (ii) prior constants, (iii) MCMC run constants. Click the "Code" button in the HTML document to see the R code.

Code

R code for restricted model with equal means, heterogeneous variances. As with the full model, the function takes three types of inputs: (i) data, (ii) prior constants, (iii) MCMC run constants. Click the "Code" button in the HTML document to see the R code.

Code

R code for restricted model with different means, homogeneous variances. As with the full model, the function takes three types of inputs: (i) data, (ii) prior constants, (iii) MCMC run constants. Click the "Code" button in the HTML document to see the R code.

Code

5 Outline of remaining sections

The remaining sections proceed as follows (see also the dynamic table of contents floating in the left margin when viewing the HTML version):

- **Full model** (different means, heterogeneous variances; abbreviated Diffm Hetv)
  - **Broad prior.**

    If the goal of the analysis were to estimate parameters (not to do model comparisons for hypothesis tests), then the analysis is nearly finished, as the next sub-steps just check the sensitivity of the posterior to the choice of prior.

  - **Prior informed by other movies, same for both.**
  - **Prior informed by subset of data from the target movies.**
  - **Check sensitivity of posterior to choice of prior.**

    If the goal of the analysis were to estimate parameters (not to do model comparisons for hypothesis tests), then the analysis is complete at this point.

    The remaining steps consider model comparisons for hypothesis tests.

- **Equal mean model** (equal means, heterogeneous variances; abbreviated Eqm Hetv)
  - **Broad prior**

- **Prior informed by other movies, same for both**
  - **Prior informed by subset of data from the target movies**
  - **Check sensitivity of posterior to choice of prior**
- **Equal variance model** (different means, homogeneous variances; abbreviated Diffm Homv)
  - **Broad prior**
  - **Prior informed by other movies, same for both**
  - **Prior informed by subset of data from the target movies**
  - **Check sensitivity of posterior to choice of prior**
- **Hypothesis tests:** *Eqm* Hetv vs *Diffm* Hetv; and Diffm *Homv* vs Diffm *Hetv*.
  - **Broad prior**
  - **Prior informed by other movies, same for both**
  - **Prior informed by subset of data from the target movies**
  - **Check sensitivity of Bayes factors to choice of prior**

6 Full model (different means, heterogeneous variances)

6.1 Broad Prior (full model)

6.1.1 Determine prior constants (full model, broad prior)

(Cf. BARG Step 1.C.)

The constants for the prior are chosen to be generically broad relative to the response scale.

Code

The value for M_μ is set at the middle of the latent scale, at 3. The value for its uncertainty, V_μ, is set to be very large relative to the latent scale, at SD=2 which is V=4. The value for M_σ is set to be middling on the latent scale, at 2, which is $M_{\log(\sigma)}$=0.693. The uncertainty of $M_{\log(\sigma)}$ is set to be large, at SD_σ=2 which is $V_{\log(\sigma)}$=0.48. Similarly, the prior for the thresholds sets $M_{\theta_k} = k + 1$, with high uncertainty on the latent scale such that SD_θ=2 or V_θ=4.

The broad-prior covariances are all set at zero. Note that setting a prior covariance to zero does not prevent the posterior distribution from having a strong correlation of parameters; the prior merely does not impose a correlation on the parameters.

In summary, the numerical settings of the broad prior constants are displayed here:

Code

```
## Broad prior mean vector:
```

Code

```
##       mu[1]       mu[2] logsigma[1] logsigma[2]   thresh[2]   thresh[3]
##       3.000       3.000       0.693       0.693       2.500       3.500
```

Code

```
## Broad prior covariance matrix:
```

Code

```
##              mu[1] mu[2] logsigma[1] logsigma[2] thresh[2] thresh[3]
## mu[1]            4     0        0.00        0.00         0         0
## mu[2]            0     4        0.00        0.00         0         0
## logsigma[1]      0     0        0.48        0.00         0         0
## logsigma[2]      0     0        0.00        0.48         0         0
## thresh[2]        0     0        0.00        0.00         4         0
## thresh[3]        0     0        0.00        0.00         0         4
```

6.1.2 Prior predictive check (full model, broad prior)

(Cf. BARG Step 1.E.)

The broad prior was specified abstractly to be symmetric on the response scale and to provide opportunity for extreme responses on either end of the scale (which can happen in ratings data). The broad prior is not intended to mimic any particular realistic response distribution. Nevertheless, a prior predictive check can at least verify the intended symmetry and extremity of the prior.

Code

Show prior predictions:

Code

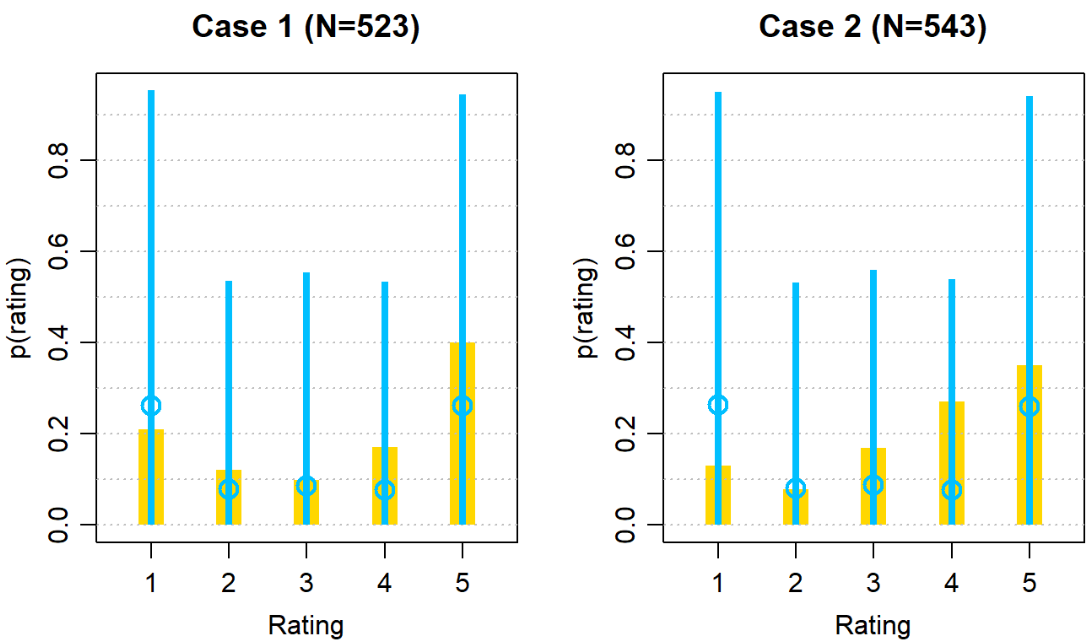

Above, it can be seen that the broad prior (blue dots are medians, bars are 95% ETI's) is indeed symmetric on the rating scale, and provides opportunity for either end of the scale to have a high probability.

6.1.3 Posterior MCMC diagnostics (full model, broad prior)

(Cf. BARG Step 2.B and 2.C.)

Run the MCMC:

Code

We must check that the MCMC chains for every parameter have converged and are long enough to provide stable estimates.

Convergence here is indicated by the *potential scale reduction factor* (PSRF)[11], which must be near 1.0 to indicate convergence. (Some practitioners deem psrf< 1.1 to be acceptable but I prefer psrf< 1.05.)

The effective length of an MCMC chain is indicated by the *effective sample size* (ESS), which refers to the sample size of the MCMC chain *not* to the sample size of the data. For reasonably stable estimates of the limits of 95% HDI's, I recommend ESS$> 10,000$ (see Section 7.5.2 of Ref 3).

There are different specific formulations of PSRF and ESS; in this application the PSRF and ESS are computed using functions supplied in the `coda` package,[12] specifically `gelman.diag()` for PSRF and `effectiveSize()` for ESS.

The table below has a row for every estimated parameter in the model. The columns indicate MCMC diagnostics and estimated values. Column headers are as follows:

- **psrfPt** is the point value of the psrf
- **psrfUpCI** is an upper bound on the psrf
- **ESS** is the effective sample size of the MCMC chain
- **50%** is the median of the estimate
- **2.5%** and **97.5%** indicate the limits of the 95% equal-tailed interval (ETI)
- **Mode** is the value with highest density computed by a kernel-density estimator
- **HDIlow** and **HDIhigh** indicate the limits of the 95% highest-density interval (HDI).

Code

Show 10 **entries** **Search:**

	psrfPt	psrfUpCI	ESS	50%	2.5%	97.5%	Mode	HDIlow	HDIhigh
mu[1]	1	1	40910.276	3.739	3.466	4.026	3.739	3.462	4.02
mu[2]	1	1.001	39181.35	3.769	3.586	3.959	3.764	3.587	3.959
sigma[1]	1	1	42062.834	2.877	2.569	3.24	2.889	2.557	3.224
sigma[2]	1	1.001	41933.594	1.956	1.781	2.159	1.943	1.771	2.147
thresh[2]	1	1	31353.65	2.315	2.195	2.442	2.315	2.19	2.436
thresh[3]	1	1	29880.04	3.195	3.068	3.319	3.206	3.07	3.321

Showing 1 to 6 of 6 entries

Previous 1 Next

Code

From the table above, we observe

- PSRF: The maximum PSRF of any parameter is 1.000445, indicating good MCMC convergence.
- ESS: The minimum ESS of any parameter is 29880.04, indicating stable estimates of limits of credible intervals.

6.1.4 Posterior predictive check (full model, broad prior)

(Cf. BARG Step 3.A.)

Here we view the model's posterior predictions visually superimposed on the data.

The graph below shows the data (as bars) with posterior-predicted median probabilities (as circles) and their 95% ETI's (as vertical segments). The ETI's are for the posterior counts of each rating, including both the uncertainty of the estimated probability and the randomness of the sample. *It can be seen that the model provides a reasonably accurate description of the data.*

Code

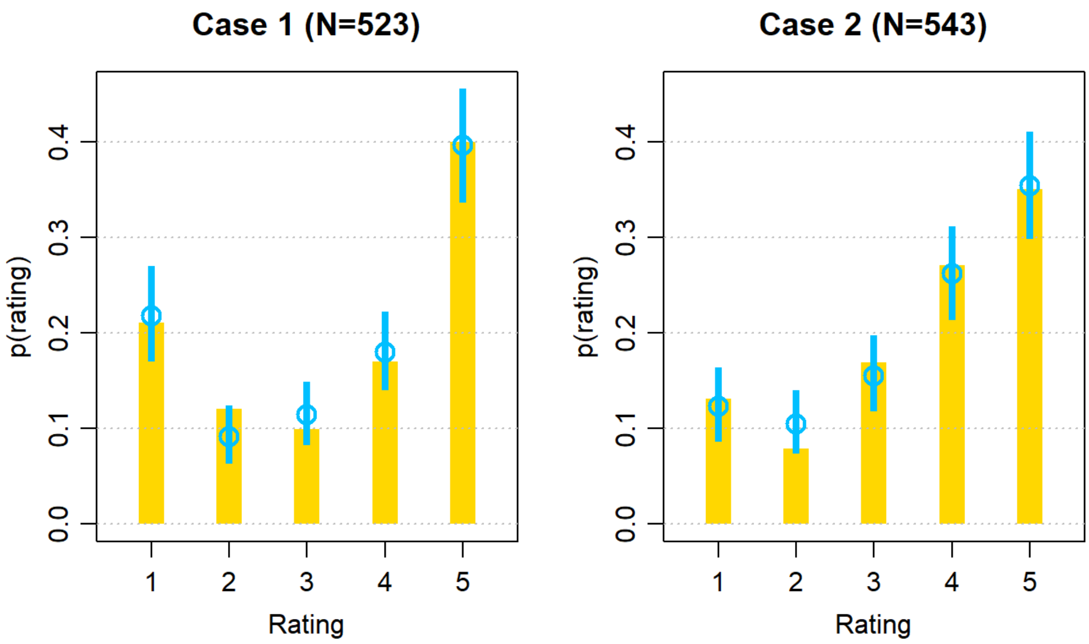

6.1.5 Posterior details (full model, broad prior)

(Cf. BARG Step 3.B.)

The table in the previous section reported numerical details of mode and HDI, and of median and ETI, for all parameters.

As mentioned previously, primary interest is in the magnitudes and uncertainties of the means and their difference, and in the magnitudes and uncertainties of the standard deviations and their difference. The difference of means can also be converted to *effect size*, measured as Cohen's $d = \left(\mu_1 - \mu_2\right) \Big/ \sqrt{(\sigma_1^2 + \sigma_2^2)/2}$, which is on the scale of pooled SD units.

Difference of means:

Code

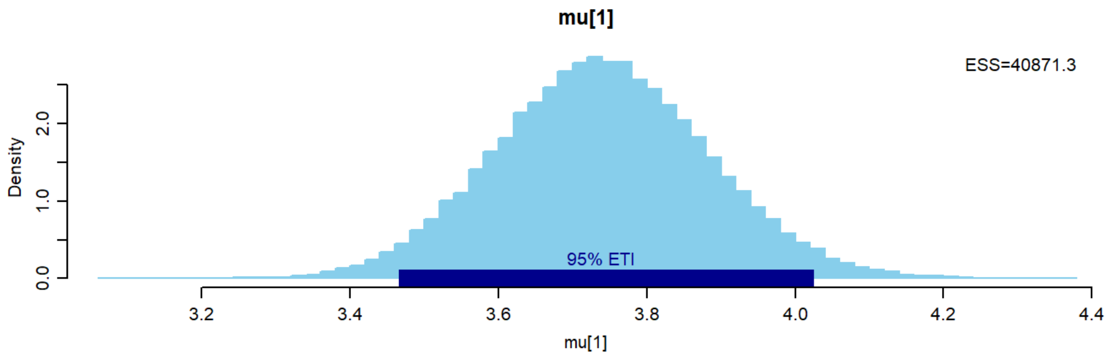

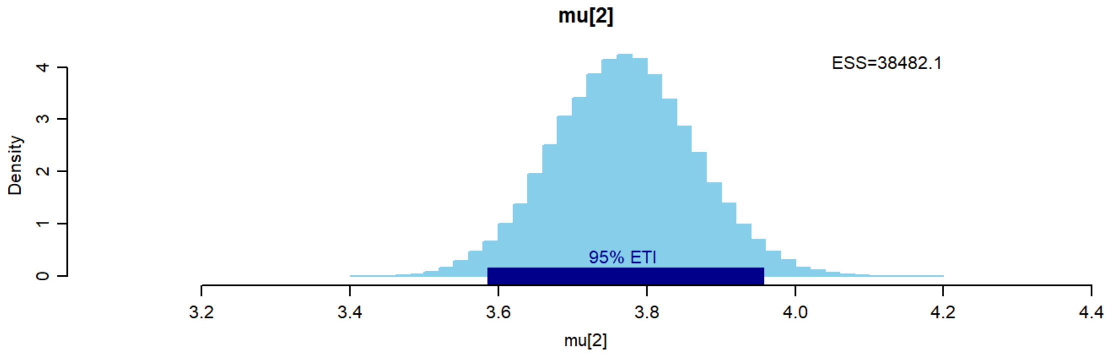

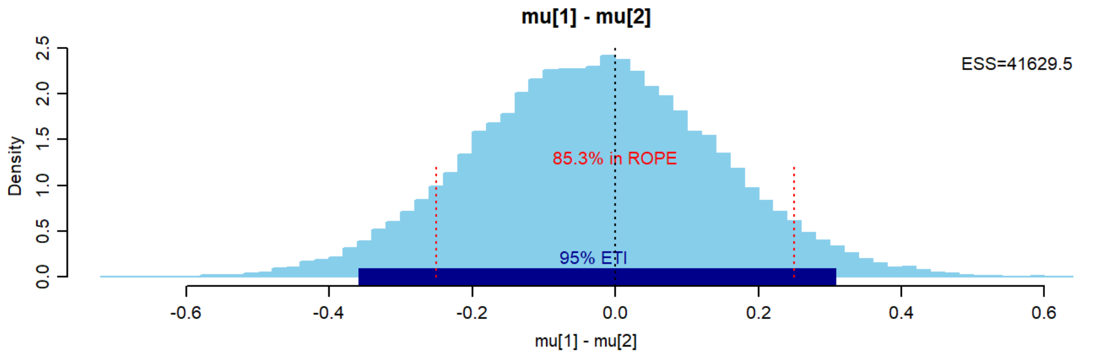

Effect size (Cohen's d), $(\mu_1 - \mu_2)\Big/\sqrt{(\sigma_1^2 + \sigma_2^2)/2}$:

Code

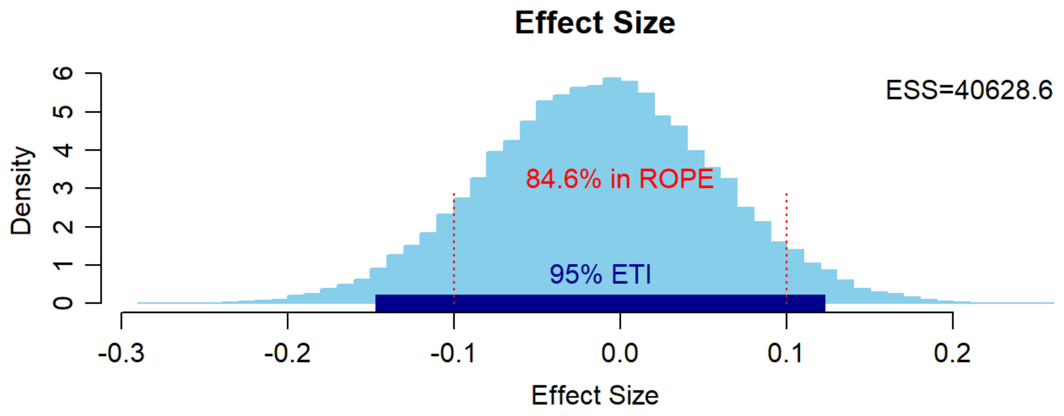

Difference of standard deviations:

Code

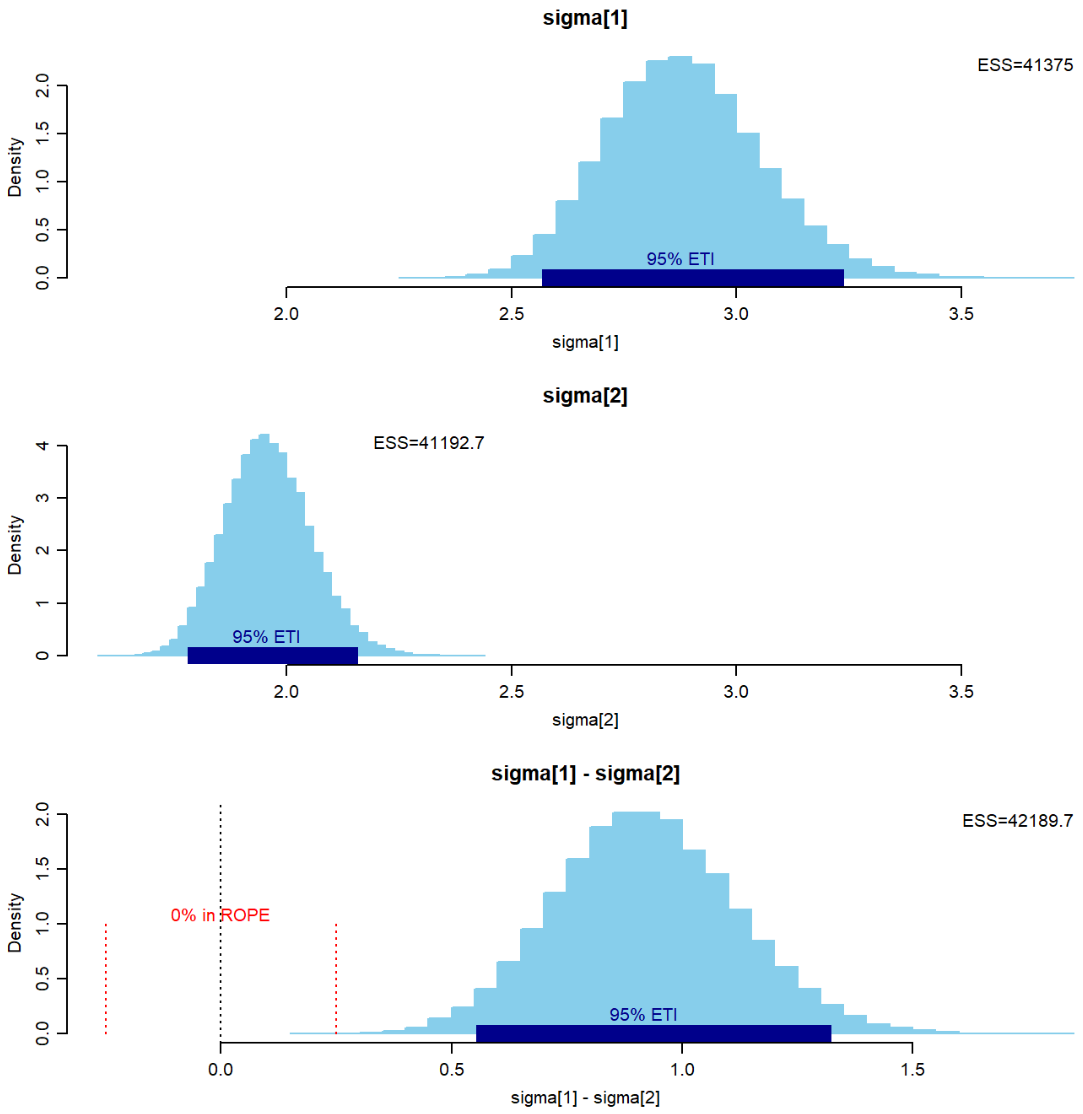

Thus, the two movies have nearly the same (latent) mean rating but they have very different (latent) SD's, with movie 1 having a much larger SD than movie 2. The analysis suggests that the primary difference between the movies is the range (SD) of reactions they evoke, not the central tendency of those reactions. For movie 2 there is a more consistent reaction than for movie 1.

Decisions:

(Cf. BARG Step 4.C.)

Some of the posterior distributions graphed above include annotations that mark a *region of practical equivalence* (ROPE) to zero difference. These ROPE's are used only for making decisions and are not an inherent part of the posterior distribution. According to different decision rules, the null value of zero difference can be *rejected* if the 95% credible interval falls completely outside the ROPE or if the percentage of the posterior distribution inside the ROPE is sufficiently small (e.g., less than 2.5%). The null value of zero difference can be *accepted for practical purposes* if the 95% credible interval falls completely inside the ROPE or if the percentage of the posterior distribution inside the ROPE is sufficiently large (e.g., greater than 95%).[13] In this application, the ROPE on the scale of effect size (Cohen's *d*) was set at -0.1 to $+0.1$ because $d = 0.1$ is *half* of a typical "small" effect size in the social sciences as suggested by Ref 14. For ROPE's on the threshold-pinned latent scale, a difference of 0.25 was deemed negligible for practical purposes (especially in light of the huge variability of ratings across people).

If a decision about the difference of means were to be made based on the indicated ROPE's, we would withhold a decision because either (i) the percentage of the posterior distribution of effect size or difference of means, while large, does not exceed a criterion of 95%, or (ii) the 95% CI does not fall entirely inside the ROPE.

If a decision about the difference of SD's were to be made based on the indicated ROPE, we would decide that the standard deviations are different because either (i) the percentage of the posterior distribution of the difference inside the ROPE is tiny (less than 2.5%), or (ii) the 95% CI of the difference falls well outside the ROPE.

Despite the accurate fit of the model to the data, there is no claim that this model is the *correct* description of the ratings. This model implies that the larger-SD movie has more extremely-high latent-scale evaluations than the smaller-SD movie, but those extremely-high latent-scale evaluations are censored (in the statistical sense) by the threshold for the highest response level. This suggests that if the response scale provided more levels for extremely high ratings, then the larger-SD movie would show more extremely high ratings than the smaller-SD movie.

If the goal of the analysis were to estimate parameters, and not to do model comparisons for hypothesis tests, then the analysis is nearly finished, as the next sub-steps just check the sensitivity of the posterior to the choice of prior. The estimates of means and SD's shown above closely match the estimates reported by Ref 1, which used a different broad prior. Additional sensitivity analysis is provided below, after re-doing the analysis with other priors.

6.2 Prior informed by other movies (full model)

6.2.1 Determine prior constants (full model, informed by other)

One approach to specifying priors is to directly intuit plausible values for the parameters and their uncertainty. I believe this approach is fraught with perils. For example, the degree of correlation between parameters is far from intuitive (to me), yet the correlation of parameters is quite strong in this application (as will be shown below). Moreover, domain-specific values for the means of threshold parameters and their uncertainties are not at all intuitive (to me). As another example, consider analogous parameters that are used across different models, such as the means in the heterogeneous-variance and homogeneous-variance models. Those means should not necessarily have the same uncertainties in the different models, and it is not intuitively obvious how different the uncertainties should be. Recall that the full model has 27 distinct constants in its prior, and setting all of those constants by intuition or even by theory seems highly implausible.

By contrast, a different approach that yields automatically consistent and meaningful priors is to start with a small set of representative data and inform all the models with that small set of representative data. In this scheme, every model begins with an extremely broad, diffuse, non-committal prior that is updated with the small set of representative data. Because the initial prior is so diffuse, its influence can be effectively erased (in typical models with modest numbers of parameters relative to the size of the small representative data set). The resulting posterior acts as the mildly informed prior for analyzing the actual data. By starting all the models this way, the models are equally informed and they are equivalently "tuned" to the same initial assumptions, not relying on user intuitions to attempt to set priors comparably across models. (For more discussion and an application to power analysis, see Section 13.2.5 of Ref 3.)

Code

To create a representative data set, I first summed the frequencies of ratings across all 34 the movies in the original set *other than the two movies being analyzed* to create a representative profile of ratings for a movie. Notice this summation automatically gives movies with many ratings more influence than movies with fewer ratings. Across the 34 movies, there were a total of 283605 ratings with an average of N=8341.3 ratings per movie. Then I used a small N that would mimic the average ratings profile while giving the smallest-probability rating a non-zero count. (Non-zero counts are required for the thresholds between levels to be estimated.) The smallest N for all non-zero counts was $N = 26$, but an N that better represented the proportions was $N = 32$.

Code

The following graph shows the actual proportion of each rating from the full data set and the small-sample proportion achieved by N=32:

Code

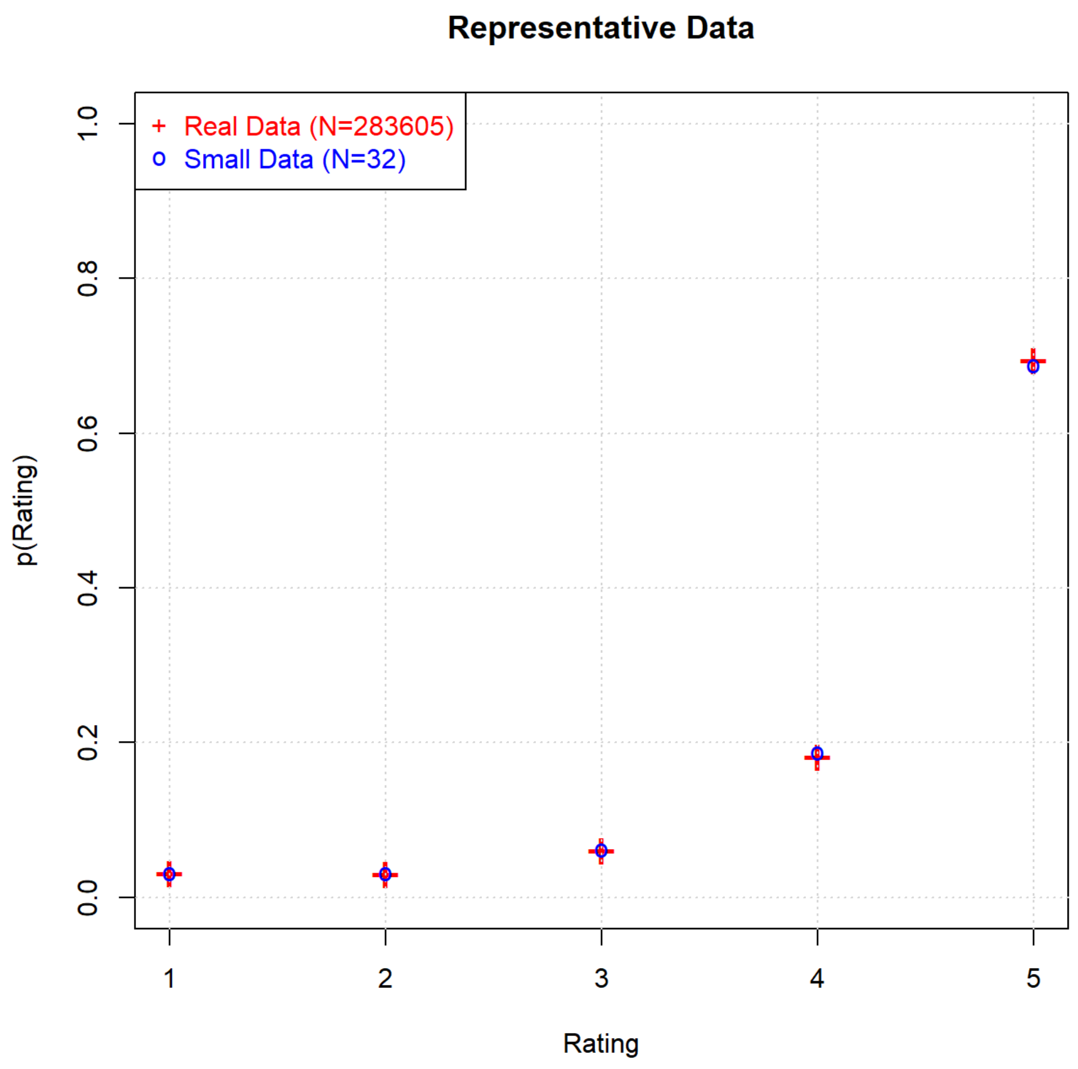

Here (below) is the small representative data set that will be used to inform all the models. Each row is a case (i.e., movie), each column is an ordinal rating (1-5), and each cell is the count for the corresponding case and rating.

Code

```
##           n1 n2 n3 n4 n5
## fPrior32  1  1  2  6 22
## fPrior32  1  1  2  6 22
```

Notice that the data are identical for the two movies, and therefore this set of data (weakly) favors a model with equal means and equal variances. These data will be used to inform every model, starting each with a very broad prior. The resulting posterior distributions will serve as mildly informed priors for subsequent model comparison. Thus, all models will be equivalently informed in the model comparison.

Now I run the broad-prior model with the small representative data. I use same broad prior as before; here is a review of the broad-prior constants:

Hide

```
print(round(paramM_DiffmHetvBroad,3))
```

```
##       mu[1]       mu[2] logsigma[1] logsigma[2]   thresh[2]   thresh[3]
##       3.000       3.000       0.693       0.693       2.500       3.500
```

Hide

```
print(round(paramVCOV_DiffmHetvBroad,3))
```

```
##             mu[1] mu[2] logsigma[1] logsigma[2] thresh[2] thresh[3]
## mu[1]           4     0        0.00        0.00         0         0
## mu[2]           0     4        0.00        0.00         0         0
## logsigma[1]     0     0        0.48        0.00         0         0
## logsigma[2]     0     0        0.00        0.48         0         0
## thresh[2]       0     0        0.00        0.00         4         0
## thresh[3]       0     0        0.00        0.00         0         4
```

I run JAGS with the small representative data:

Code

Check MCMC diagnostics:

Code

Show 10 **entries** **Search:**

	psrfPt	psrfUpCI	ESS	50%	2.5%	97.5%	Mode	HDIlow	HDIhigh
mu[1]	1	1.001	14367.143	5.426	4.522	6.841	5.36	4.449	6.72
mu[2]	1	1.001	16833.404	5.425	4.52	6.825	5.345	4.445	6.698
sigma[1]	1	1	15821.525	2.192	1.367	3.752	2.083	1.245	3.521
sigma[2]	1	1	15780.422	2.191	1.374	3.743	2.038	1.248	3.511
thresh[2]	1	1.001	17491.289	2.226	1.701	2.941	2.142	1.645	2.868
thresh[3]	1.001	1.002	17378.817	3.109	2.458	3.688	3.134	2.474	3.7

Showing 1 to 6 of 6 entries   Previous 1 Next

Code

- PSRF: The maximum PSRF of any parameter is 1.000702, indicating good MCMC convergence.
- ESS: The minimum ESS of any parameter is 14367.14, indicating stable estimates of limits of credible intervals.

Posterior predictive check:

I check here that the posterior distribution accurately reflects the small representative data set. The graph below shows the data (as bars) with posterior-predicted probabilities (as circles) and their 95% ETI's (as vertical segments). The ETI's are for the posterior counts of each rating, including both the uncertainty of the estimated probability and the randomness of the sample. It can be seen that the model provides an accurate description of the data.

Code

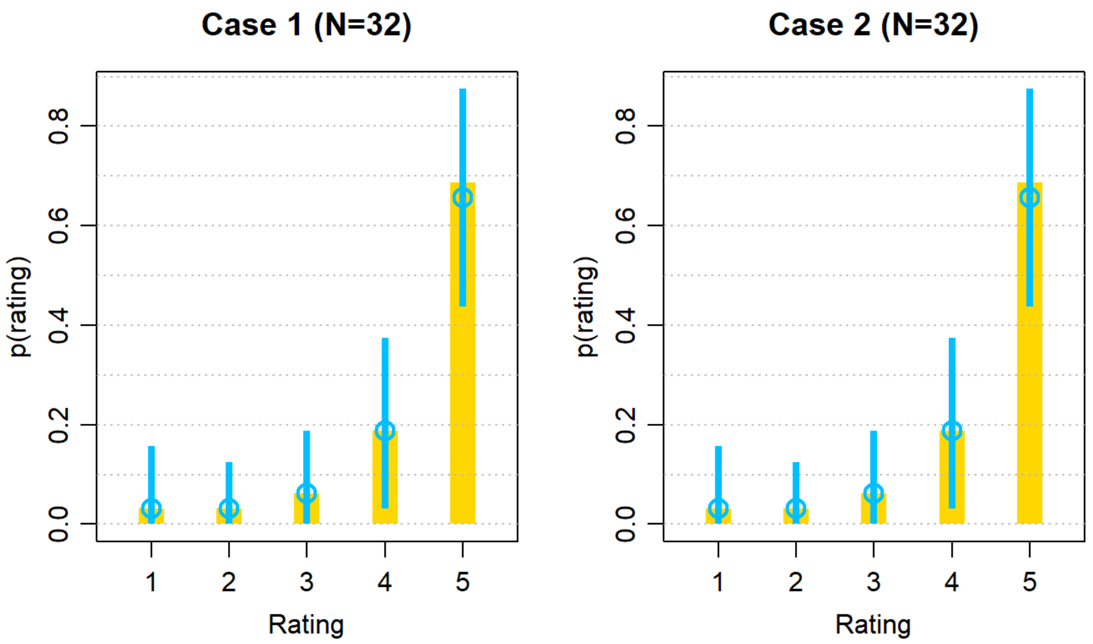

Examine posterior (from small other-movie data, broad prior):

This posterior distribution from the small representative data will serve as the prior for subsequent analysis of the actual data. Pair-wise scatter plots of parameters in MCMC are shown below, where it can be seen that there can be strong covariation of `mu[j]` with `logsigma[j]` and among the thresholds. The prior distribution for subsequent analysis with the full data will approximate the means and covariances of this mildly informed posterior distribution.

Code

Code

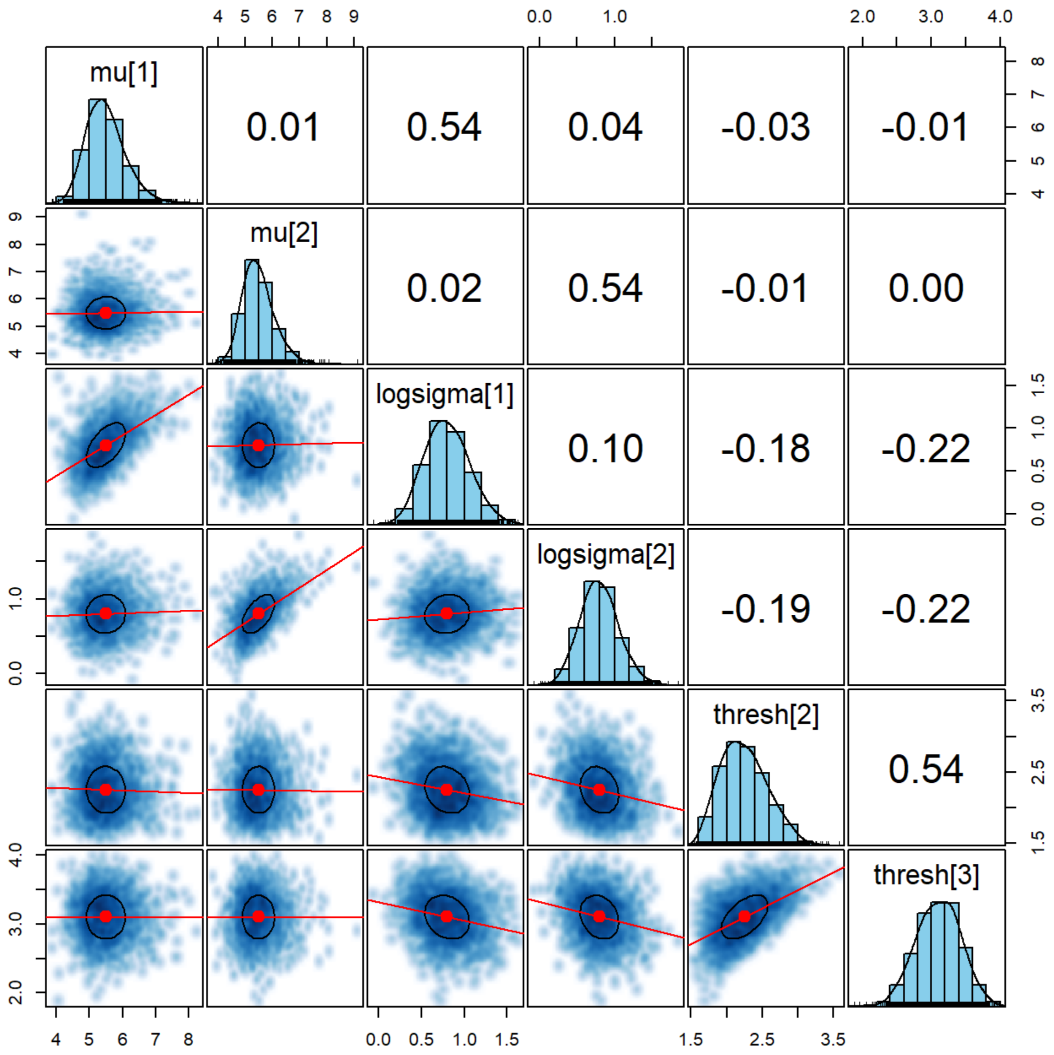

Next I compute and display the prior constants from the posterior of the small representative data. Note that these constants are covariances, not correlations, so they will not numerically equal the correlations in the plot above (although they are algebraically equivalent).

Code

Here are the prior constants:

Hide

```
print(round(paramM_DiffmHetvOther,3) )
```

```
##       mu[1]       mu[2] logsigma[1] logsigma[2]   thresh[2]   thresh[3]
##       5.495       5.494       0.793       0.794       2.251       3.099
```

Hide

```
print(round(paramVCOV_DiffmHetvOther,3) )
```

```
##              mu[1]  mu[2] logsigma[1] logsigma[2] thresh[2] thresh[3]
## mu[1]        0.354 -0.002       0.083       0.000    -0.006    -0.004
## mu[2]       -0.002  0.348       0.000       0.082    -0.006    -0.003
## logsigma[1]  0.083  0.000       0.067       0.004    -0.017    -0.018
## logsigma[2]  0.000  0.082       0.004       0.066    -0.017    -0.019
## thresh[2]   -0.006 -0.006      -0.017      -0.017     0.107     0.057
## thresh[3]   -0.004 -0.003      -0.018      -0.019     0.057     0.100
```

6.2.2 Prior predictive check (full model, prior informed by other)

If the mathematical prior were an exact copy of the posterior from the mildly informed fit, then a prior predictive check would be superfluous. But because the mathematical prior is merely a close approximation of the posterior from the mildly informed fit, a prior predictive check verifies that the prior really does mimic the small data as intended.

Code

Show prior predictions:

Code

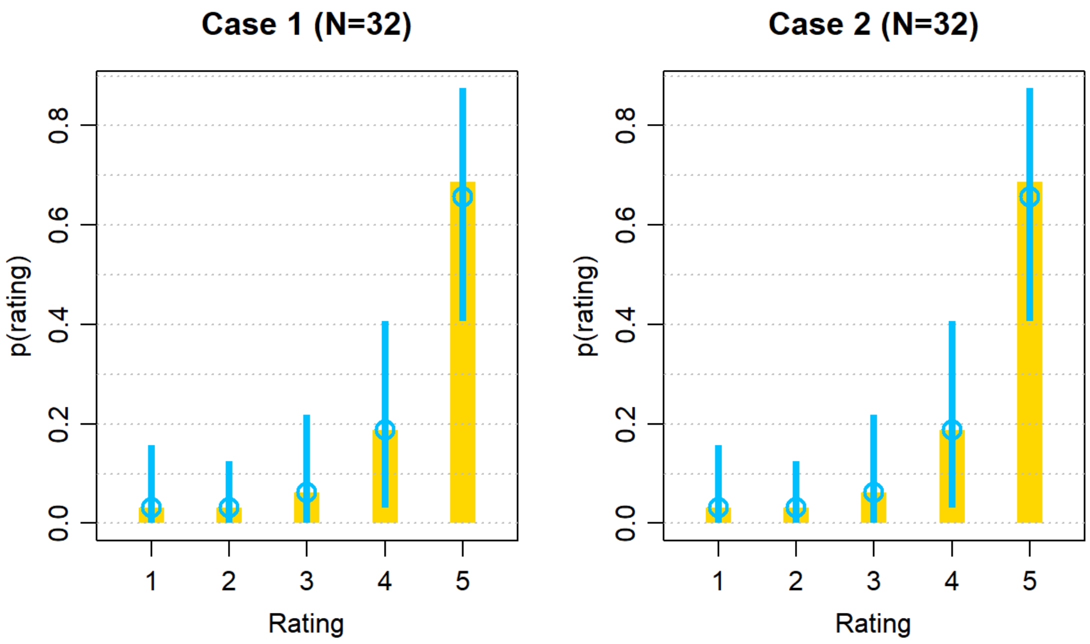

Above, it can be seen that the mildly-informed prior does mimic the small data.

6.2.3 Posterior MCMC diagnostics (full model, prior informed by other)

Now I run actual data using the mildly informed prior. This full model is the same as the go-to model for parameter estimation, but now it will use a mildly informed prior. The posterior that results from the mildly-informed prior should be essentially the same as what would be obtained by starting with the broad prior but using data that merges the actual data with the small representative data set. Because the actual data set has N=533 per movie while the prior data set has N=32 per movie, the posterior from the mildly informed prior should be only a little different from the posterior from the broad prior. But for purposes of model comparison it can be important that all the models have priors that are equivalently and usefully informed.

Run the MCMC on the actual data with the mildly informed prior:

Code

Code

Show 10 **entries**

Search:

	psrfPt	psrfUpCI	ESS	50%	2.5%	97.5%	Mode	HDIlow	HDIhigh
mu[1]	1	1	35102.559	3.872	3.606	4.155	3.88	3.595	4.141
mu[2]	1	1	34051.995	3.82	3.639	4.011	3.817	3.635	4.006
sigma[1]	1	1	35064.272	2.794	2.505	3.136	2.762	2.495	3.122
sigma[2]	1	1.001	36772.645	1.94	1.768	2.137	1.938	1.76	2.127
thresh[2]	1	1.002	24883.382	2.319	2.202	2.443	2.317	2.202	2.442
thresh[3]	1	1.001	25849.094	3.196	3.073	3.319	3.197	3.073	3.319

Showing 1 to 6 of 6 entries

Previous 1 Next

Code

- PSRF: The maximum PSRF of any parameter is 1.000461, indicating good MCMC convergence.
- ESS: The minimum ESS of any parameter is 24883.38, indicating stable estimates of limits of credible intervals.

6.2.4 Posterior predictive check (full model, prior informed by other)

The graph below shows the data (as bars) with posterior-predicted probabilities (as circles) and their 95% ETI's (as vertical segments). The ETI's are for the posterior counts of each rating, including both the uncertainty of the estimated probability and the randomness of the sample. *It can be seen that the model provides an accurate description of the data.*

Code

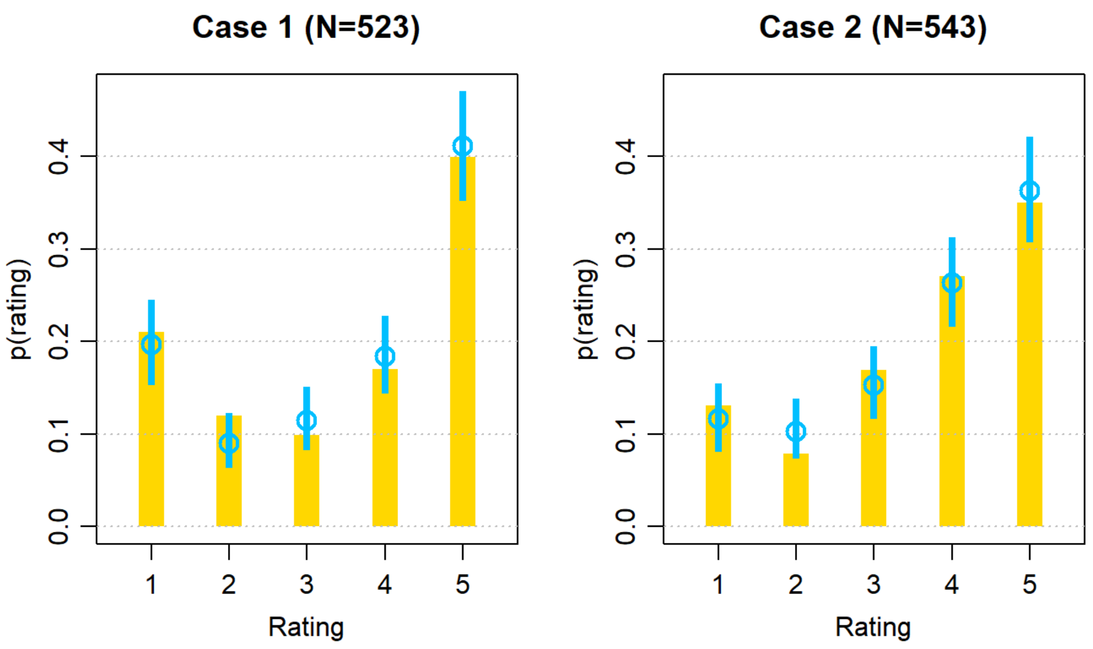

6.2.5 Posterior details (full model, prior informed by other)

Difference of means:

Code

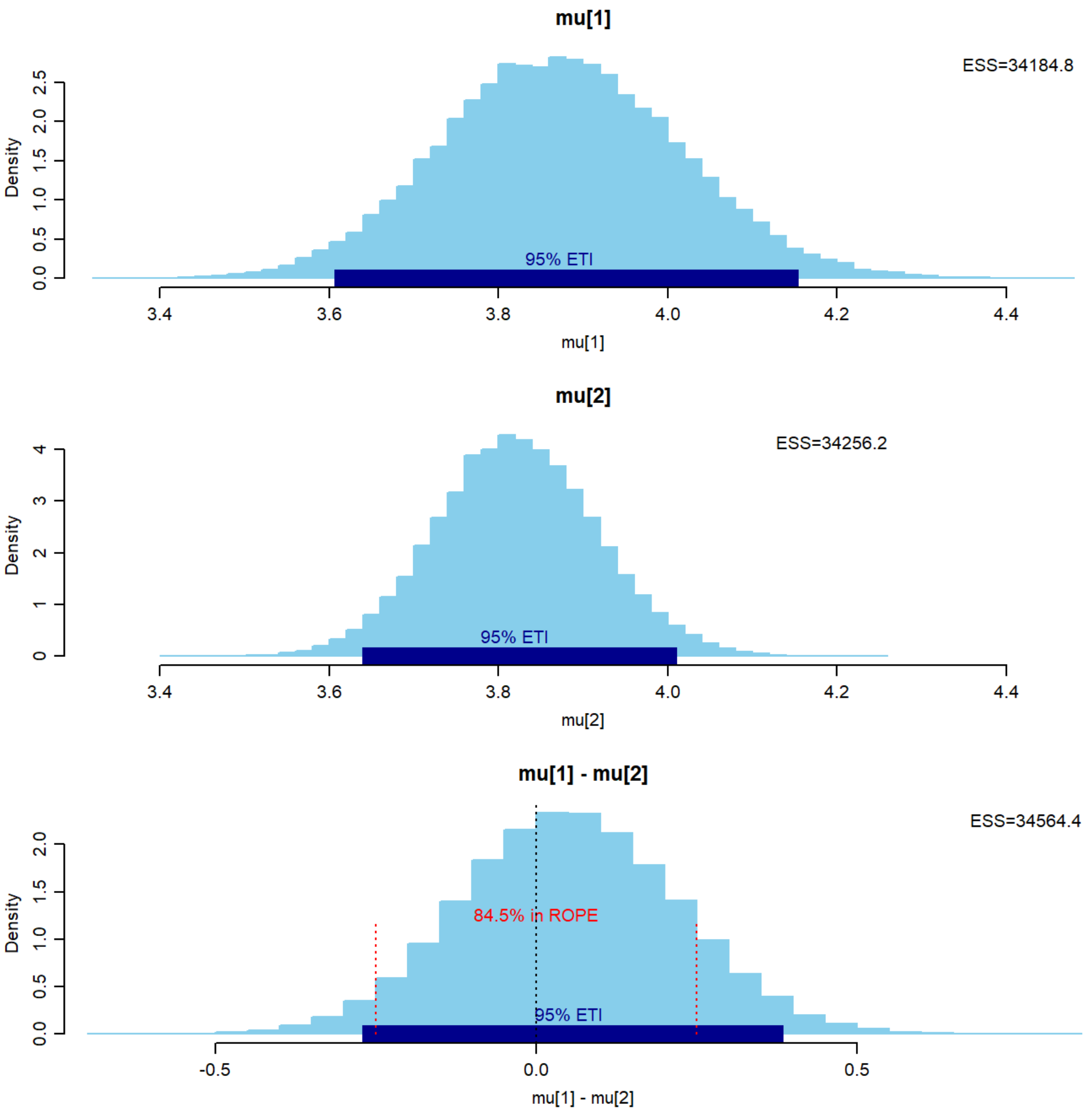

Effect size (Cohen's d), $(\mu_1 - \mu_2) \Big/ \sqrt{(\sigma_1^2 + \sigma_2^2)/2}$:

Code

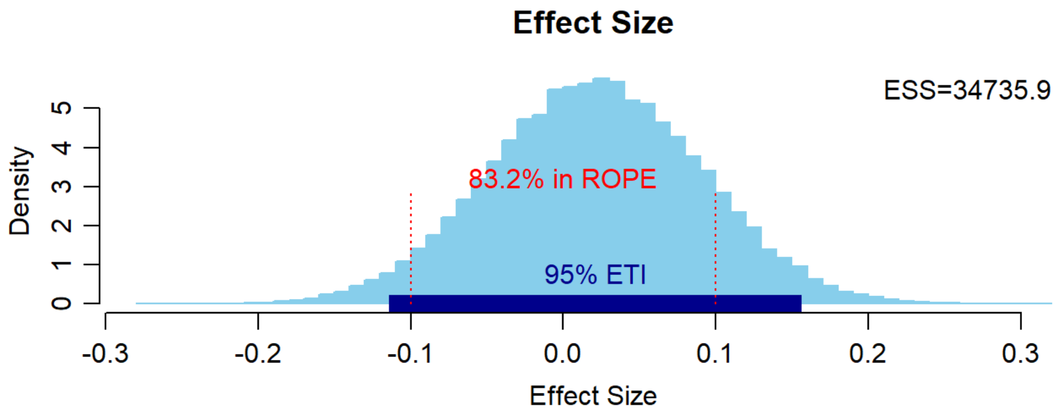

Difference of standard deviations:

Code

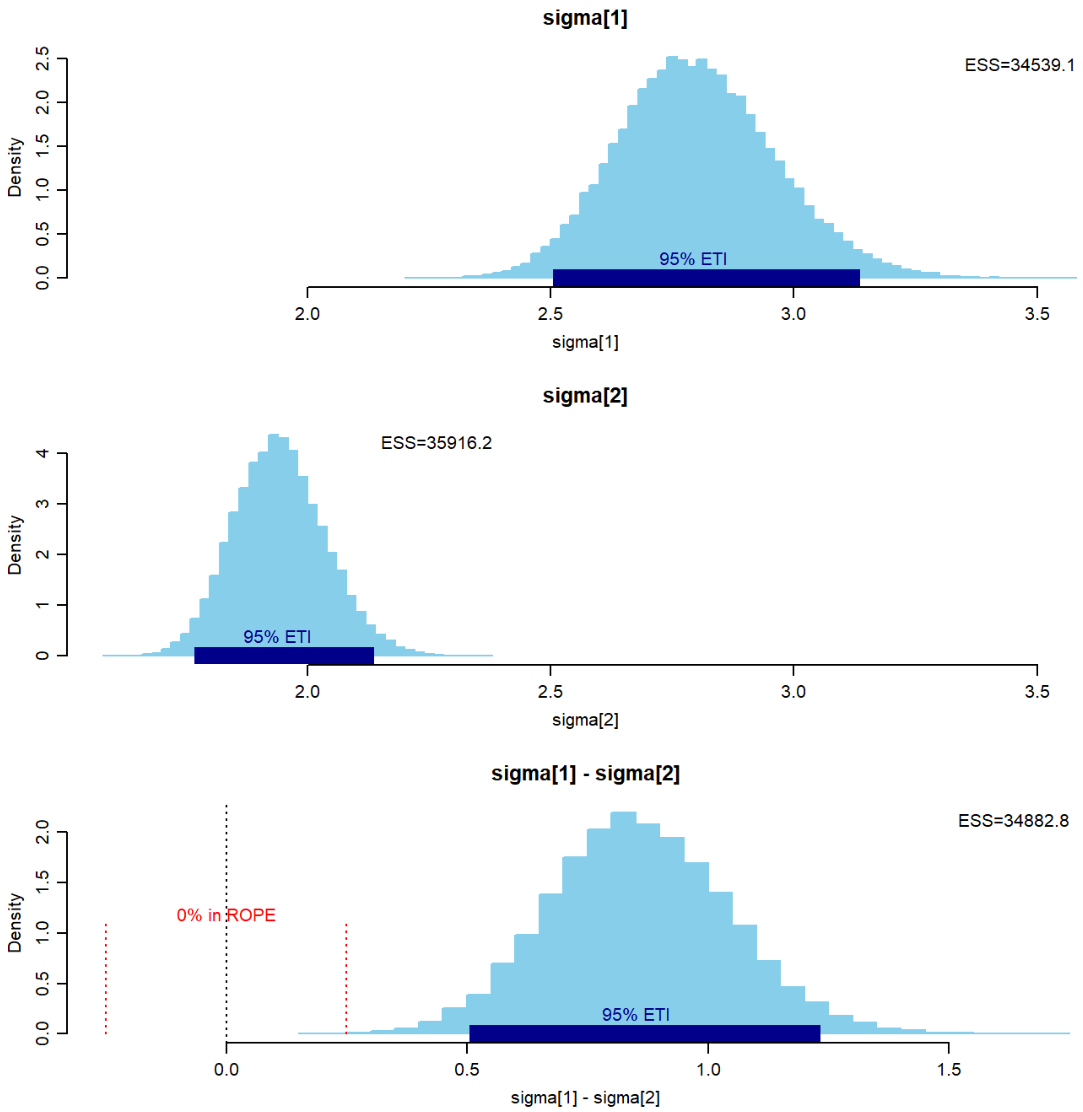

Above, the posterior estimates of the parameters are very similar to the estimates obtained from using a broad prior. A direct comparison will be shown in a subsequent section on *sensitivity analysis*.

6.3 Prior informed by subset of data (full model)

6.3.1 Determine prior constants (full model, prior informed by subset)

Here I apply the method of using a small subset from the actual data to inform the priors. This is related to the method of an 'intrinsic' Bayes factor[15] and an example application was reported by Ref 16. I modify the procedure somewhat by, instead of taking a *random* subset of the data to inform the priors, constructing a small representative data set that accurately mimics the full data. (Also, after informing the priors with the small representative data set, I will use the full data set instead of the complement of the data set after removing the representative data. I do this for consistency with the procedure that used data from other movies to inform the prior.) For each case (i.e., movie), the proportion of ratings at each level was multiplied by a selected small N and the result was rounded to the nearest integer. For consistency with the previous representative data set, I used N=32 in each case. Here is the result:

Code

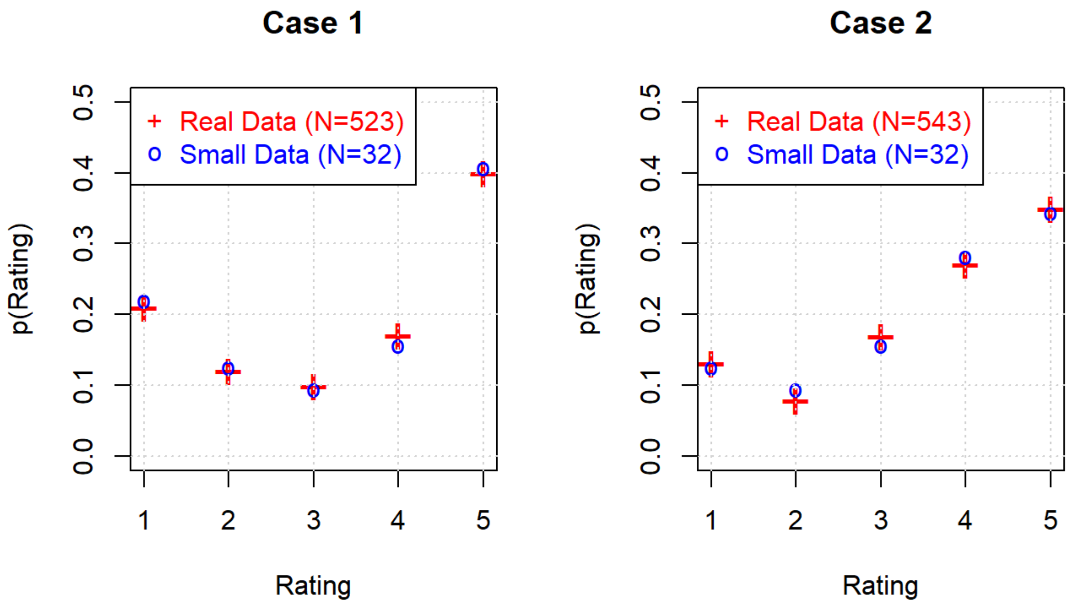

Code

A frequency table of the small data set is shown below. The row labels (9, 15) are the case numbers of the movies.

Code

```
##    n1 n2 n3 n4 n5
## 9   7  4  3  5 13
## 15  4  3  5  9 11
```

Notice that this small data set does *not* have equal means and equal variances (unlike the data set constructed from other movies).

Now I run the broad-prior model with small representative data. I use same broad prior as before. For review, here are the broad-prior constants:

Hide

```
print(round(paramM_DiffmHetvBroad,3))
```

```
##       mu[1]       mu[2] logsigma[1] logsigma[2]    thresh[2]    thresh[3]
##       3.000       3.000       0.693       0.693        2.500        3.500
```

Hide

```
print(round(paramVCOV_DiffmHetvBroad,3))
```

```
##             mu[1] mu[2] logsigma[1] logsigma[2] thresh[2] thresh[3]
## mu[1]           4     0        0.00        0.00         0         0
## mu[2]           0     4        0.00        0.00         0         0
## logsigma[1]     0     0        0.48        0.00         0         0
## logsigma[2]     0     0        0.00        0.48         0         0
## thresh[2]       0     0        0.00        0.00         4         0
## thresh[3]       0     0        0.00        0.00         0         4
```

Run JAGS with the small representative data:

Code

MCMC diagnostics:

Code

Show 10 **entries** **Search:**

	psrfPt	psrfUpCI	ESS	50%	2.5%	97.5%	Mode	HDIlow	HDIhigh
mu[1]	1	1.001	23975.22	3.697	2.533	5.021	3.67	2.48	4.953
mu[2]	1	1.001	24919.22	3.762	3.003	4.608	3.753	2.984	4.583
sigma[1]	1	1	25156.519	3.069	2.035	5.069	2.822	1.895	4.789
sigma[2]	1	1	26832.623	2.003	1.412	3.067	1.884	1.325	2.915
thresh[2]	1	1.001	19226.394	2.376	1.951	2.883	2.336	1.938	2.866
thresh[3]	1	1.001	19231.954	3.221	2.727	3.678	3.243	2.741	3.691

Showing 1 to 6 of 6 entries

Previous 1 Next

Code

- PSRF: The maximum PSRF of any parameter is 1.000365, indicating good MCMC convergence.
- ESS: The minimum ESS of any parameter is 19226.39, indicating stable estimates of limits of credible intervals.

Posterior predictive check:

We check here that the posterior distribution accurately reflects the small representative data set. The graph below shows the data (as bars) with posterior-predicted probabilities (as circles) and their 95% ETI's (as vertical segments). The ETI's are for the posterior counts of each rating, including both the uncertainty of the estimated probability and the randomness of the sample. It can be seen that the model provides an accurate description of the data.

Code

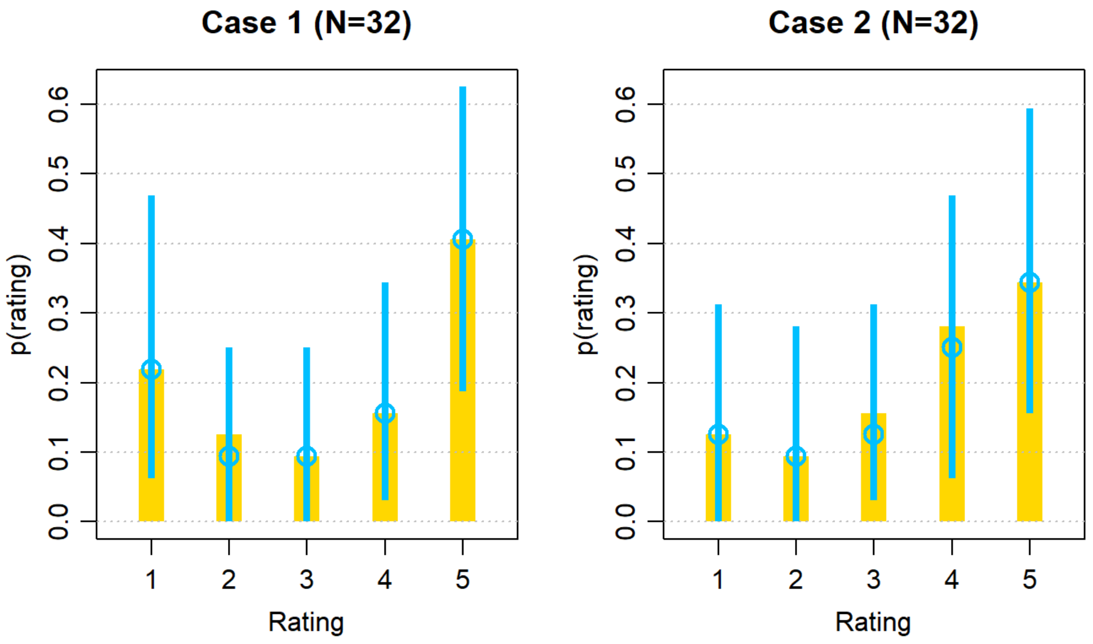

Examine posterior:

This posterior distribution from the small representative data will serve as the prior for subsequent analysis of the actual data. Pair-wise scatter plots of parameters in MCMC are shown below, where it can be seen that there can be strong covariation among some parameters. The prior distribution for subsequent models will approximate the means and covariances of this mildly informed posterior distribution.

Code

Code

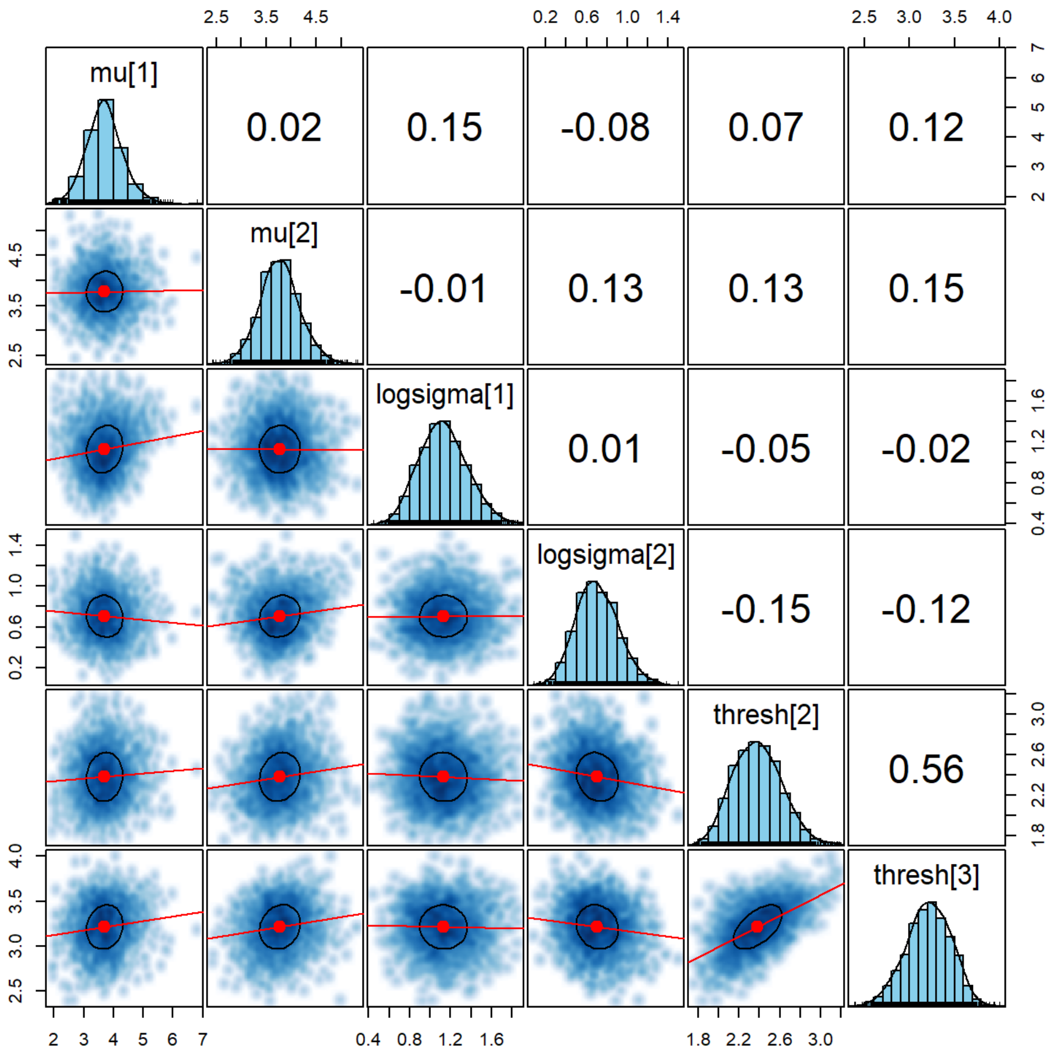

Now I compute the prior constants from the posterior of the small representative data:

Code

Here are the prior constants (again, these are covariances, not correlations, so they will not numerically equal the correlations in the pairs plot above):

Hide

```
print(round(paramM_DiffmHetvSubset,3) )
```

```
##       mu[1]       mu[2] logsigma[1] logsigma[2]   thresh[2]   thresh[3]
##       3.717       3.771       1.133       0.706       2.387       3.217
```

Hide

```
print(round(paramVCOV_DiffmHetvSubset,3) )
```

```
##              mu[1] mu[2] logsigma[1] logsigma[2] thresh[2] thresh[3]
## mu[1]        0.383 0.004       0.021      -0.002     0.011     0.012
## mu[2]        0.004 0.163       0.000       0.012     0.013     0.017
## logsigma[1]  0.021 0.000       0.054       0.000    -0.003    -0.002
## logsigma[2] -0.002 0.012       0.000       0.039    -0.006    -0.004
## thresh[2]    0.011 0.013      -0.003      -0.006     0.058     0.033
## thresh[3]    0.012 0.017      -0.002      -0.004     0.033     0.060
```

6.3.2 Prior predictive check (full model, prior informed by subset)

If the mathematical prior were an exact copy of the posterior from the mildly informed fit, then a prior predictive check would be superfluous. But because the mathematical prior is merely a close approximation of the posterior from the mildly informed fit, a prior predictive check verifies that the prior really does mimic the small data as intended.

Code

Show prior predictions:

Code

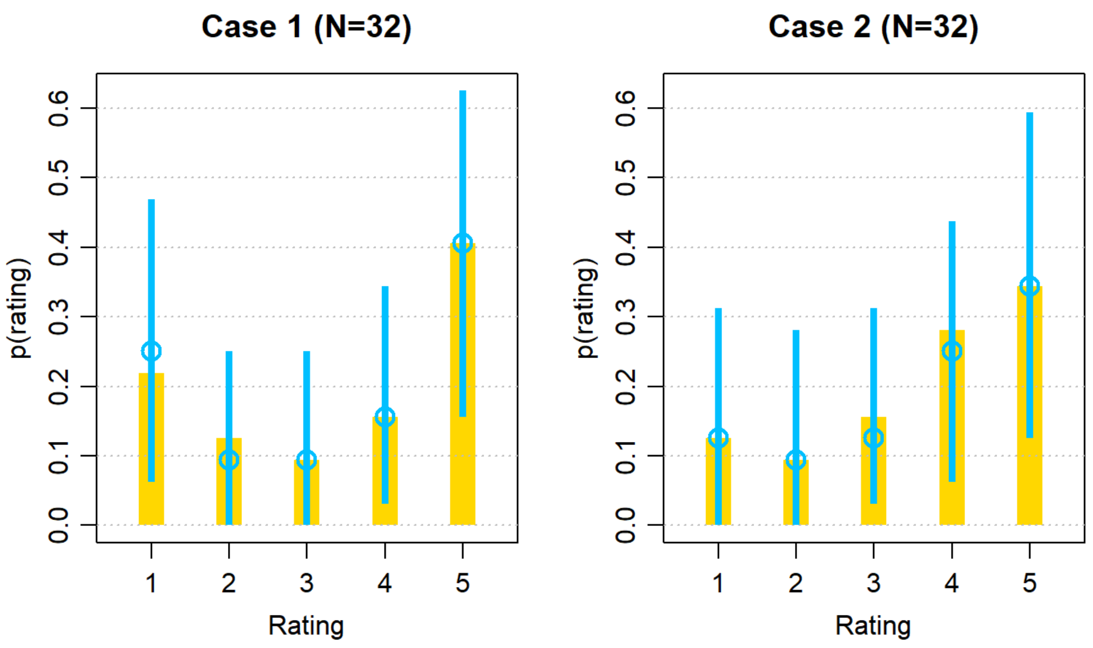

Above, it can be seen that the mildly-informed prior does mimic the small representative data.

6.3.3 Posterior MCMC diagnostics (full model, prior informed by subset)

Run the MCMC on the actual data with the mildly informed prior:

Code

Code

Show 10 entries

Search:

	psrfPt	psrfUpCI	ESS	50%	2.5%	97.5%	Mode	HDIlow	HDIhigh
mu[1]	1	1	37400.761	3.745	3.471	4.026	3.736	3.466	4.02
mu[2]	1	1.001	36685.298	3.772	3.593	3.956	3.78	3.587	3.949
sigma[1]	1	1	38261.328	2.896	2.592	3.253	2.895	2.58	3.238
sigma[2]	1	1	37434.523	1.959	1.787	2.155	1.943	1.781	2.148
thresh[2]	1	1	27151.868	2.319	2.202	2.442	2.314	2.202	2.442
thresh[3]	1	1	26210.191	3.197	3.074	3.316	3.208	3.075	3.318

Showing 1 to 6 of 6 entries

Previous 1 Next

Code

- PSRF: The maximum PSRF of any parameter is 1.000349, indicating good MCMC convergence.
- ESS: The minimum ESS of any parameter is 26210.19, indicating stable estimates of limits of credible intervals.

6.3.4 Posterior predictive check (full model, prior informed by subset)

The graph below shows the data (as bars) with posterior-predicted probabilities (as circles) and their 95% ETI's (as vertical segments). The ETI's are for the posterior counts of each rating, including both the uncertainty of the estimated probability and the randomness of the sample. *It can be seen that the model provides an accurate description of the data.*

Code

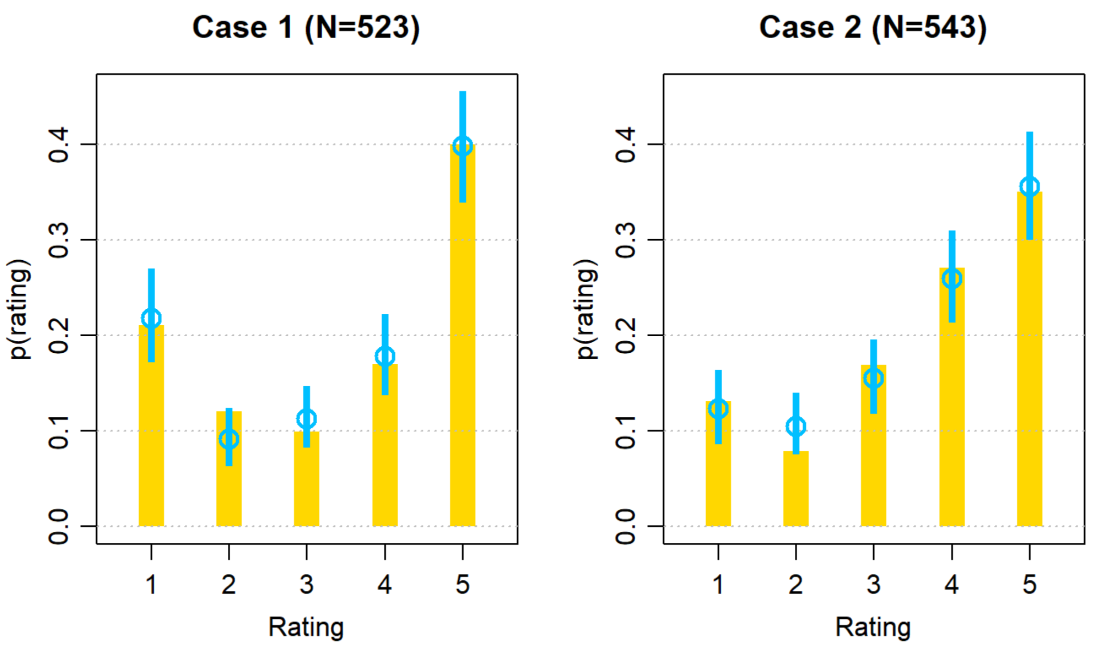

6.3.5 Posterior details (full model, prior informed by subset)

Difference of means:

Code

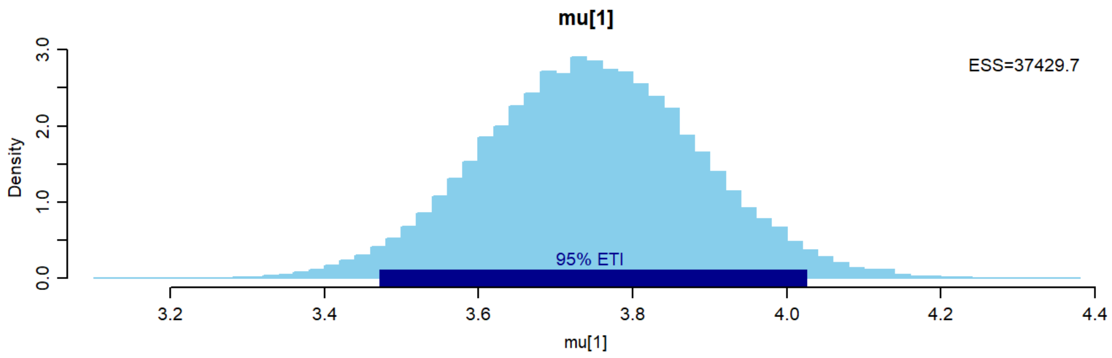

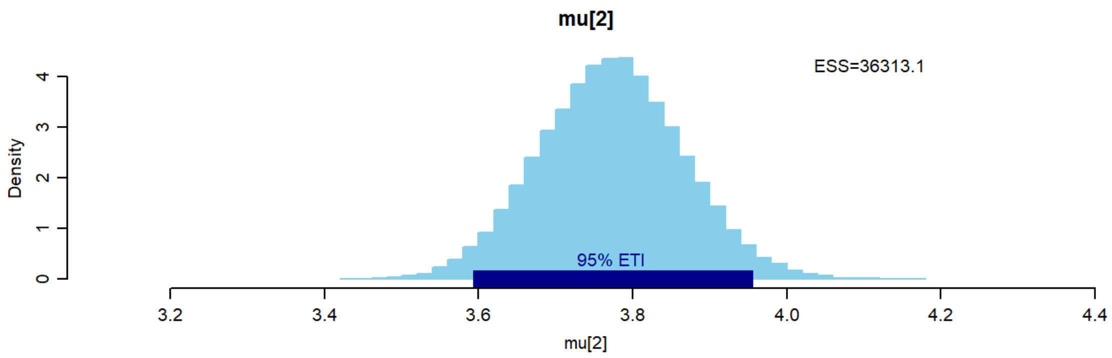

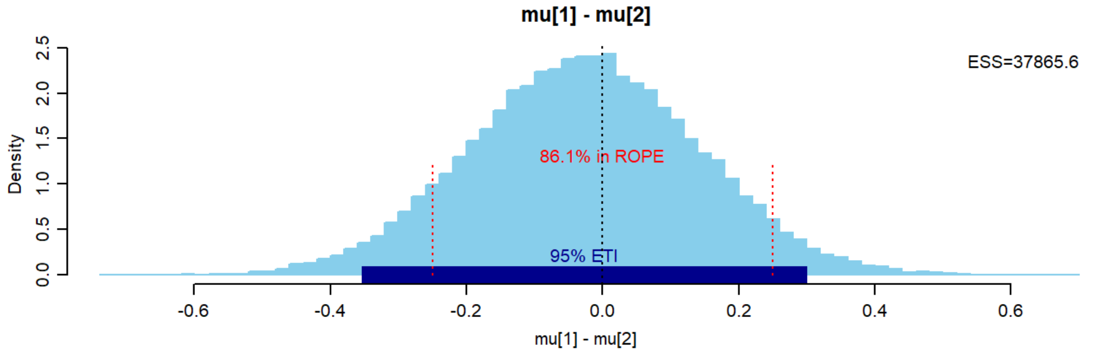

Effect size (Cohen's d), $(\mu_1 - \mu_2)\Big/\sqrt{(\sigma_1^2 + \sigma_2^2)/2}$:

Code

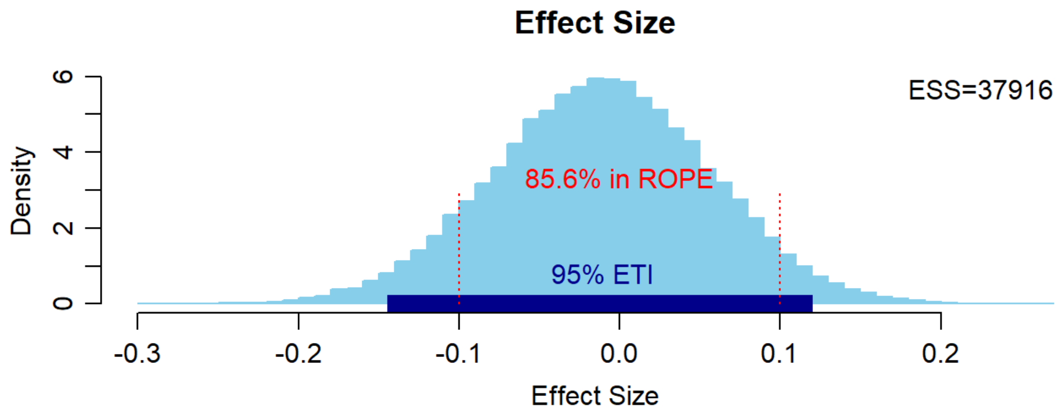

Difference of standard deviations:

Code

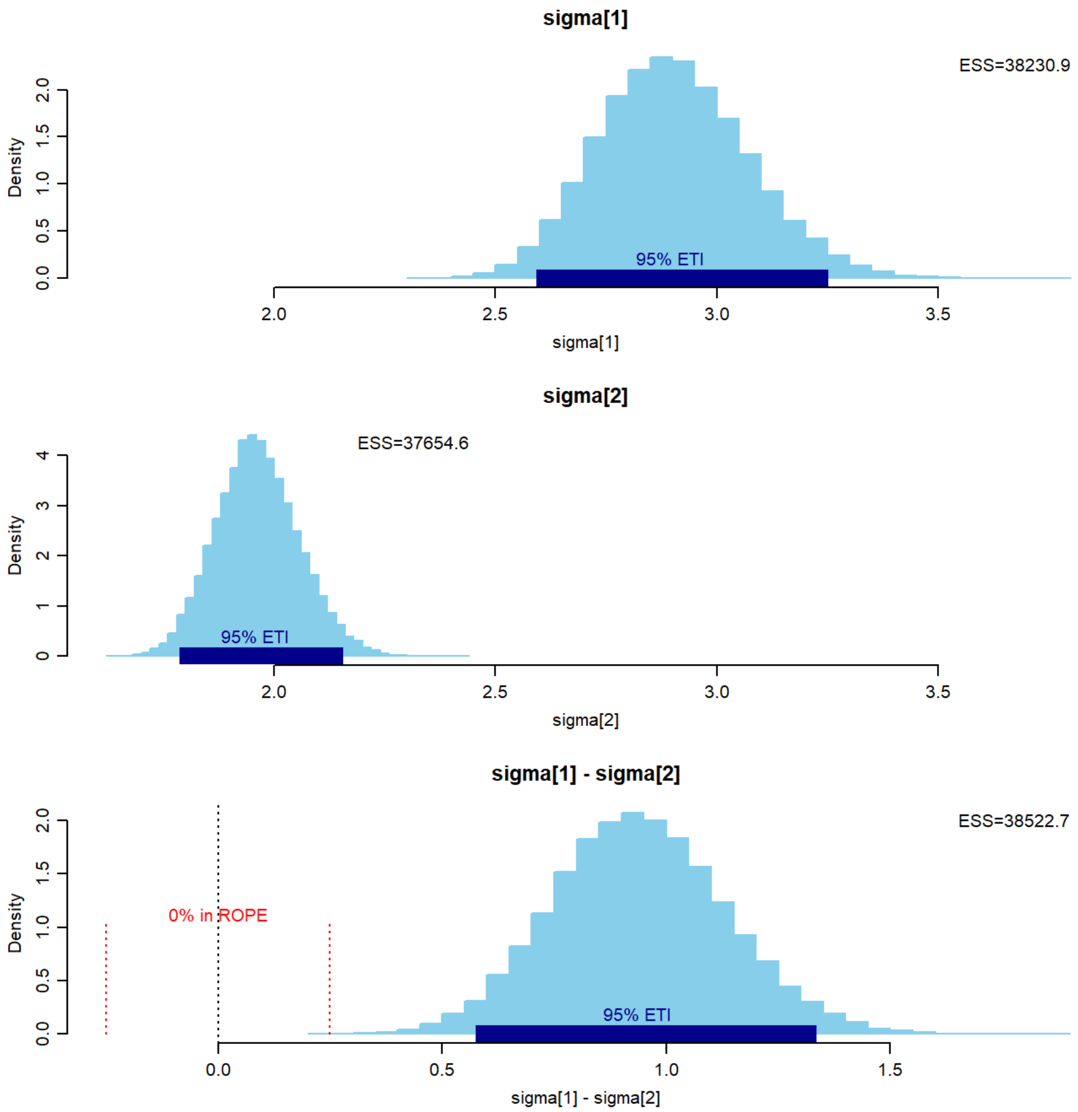

The parameter estimates, above, are very similar to those obtained from other priors. A direct comparison is presented in the following section on *sensitivity analysis*

6.4 Sensitivity to prior (full model)

(Cf. BARG Step 5.A, 5.B, 5.E.)

By inspecting the summary tables and graphs of the posterior distributions, above, it can be seen that there is *very little change* in the posterior estimation when starting with the mildy informed prior, relative to the broad prior.

MCMC distributions can be visually compared by superimposing their smoothed histograms. This is useful for visualizing density-based values such as mode and HDI. Unfortunately, smoothed histograms are inherently prone to approximation error due to the arbitrary width of the smoothing kernel, resulting in random “ripples” in the smoothed curve.

Another useful way to compare MCMC distributions is with the empirical cumulative distribution function (ECDF). (For example, ECDF’s are at the core of the Kolmogorov–Smirnov test of differences between distributions, but we will not be applying that frequentist method here.) One advantage of ECDF’s over smoothed histograms is that no kernel smoother or binning is involved, so there is no loss of resolution. Cumulative distribution functions are useful for visualizing cumulative values such as median and ETI.

Below are graphical comparisons of the marginal posteriors from the two priors. The plot of posterior density relies on smoothing by a kernel with width determined heuristically. The posterior density is a natural way to view the mode and HDI. The plot of cumulative probability does not rely on smoothing (but is effectively smoothed by the pixel resolution). The cumulative probability is a natural way to view the median and ETI.

The impact of the different priors is made clear by the plot, but the magnitude of difference relative to the credible intervals and relative to the ROPE is very small.

Code

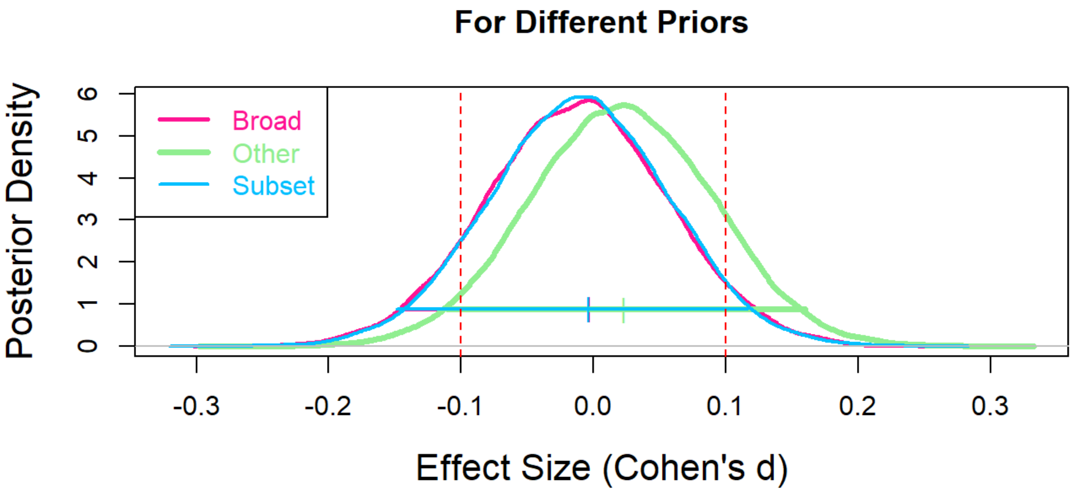

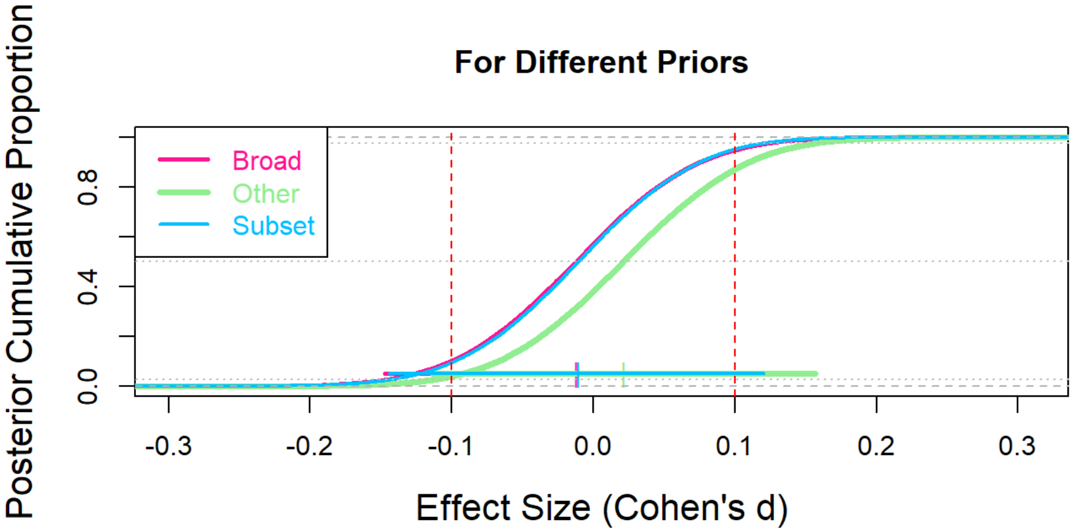

Above: Effect Size estimates from different priors. Broad = broad prior, Other = informed by other movies, Subset = informed by subset.

Code

Code

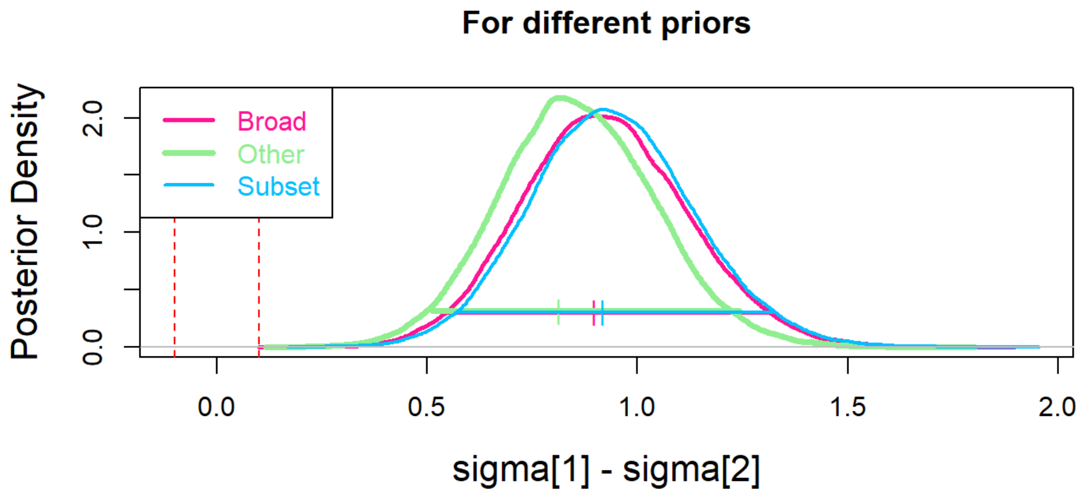

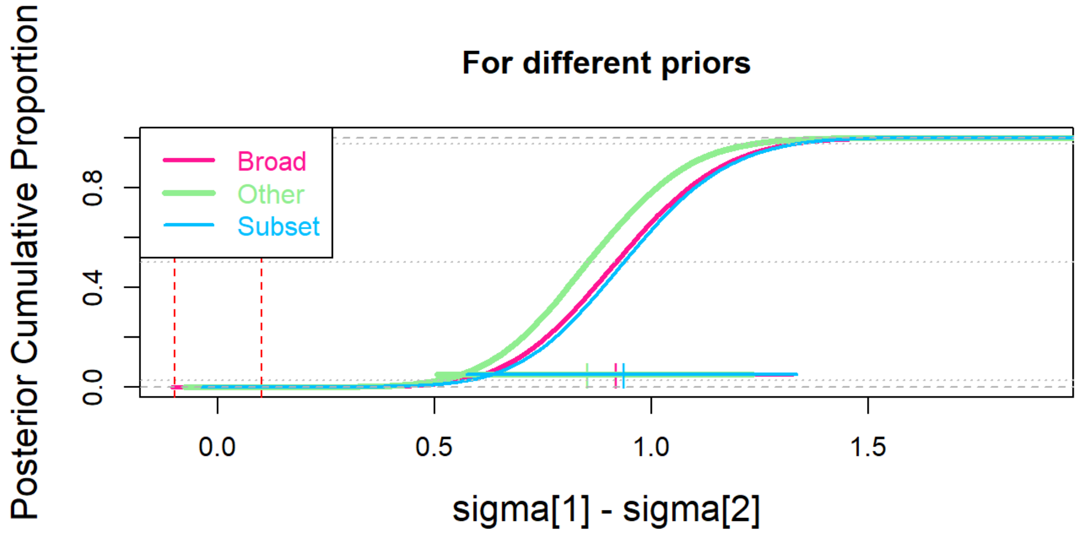

Above: Standard deviation (difference) estimates from different priors. Broad = broad prior, Other = informed by other movies, Subset = informed by subset.

Code

Notice above that the posterior estimates are virtually identical for the broad prior and the subset-informed prior.

The posterior estimates from the other-informed prior are slightly different, but only slightly relative to the uncertainty (width) of the distribution and relative to the ROPE. That is, the conclusion remains the same that there is a notable non-zero difference of standard deviations, but only a tiny difference of means if any. I suspect that the reason the other-informed prior has a visible effect is that this equal-SD prior pulls together the estimates of the two SD’s, thereby forcing the means to compensate. We will see later that the restricted equal-SD model does not fit the data very well.

If the goals of the analysis were to estimate parameters and assess null values from the posterior distribution of the estimates (not to do model comparisons for hypothesis tests), then the analysis is complete at this point. The remaining sections consider model comparisons for hypothesis tests.

---

7 Restricted Model: Equal means, heterogeneous variances

This model allows heterogeneous variances but restricts there to be **a single mean used simultaneously for all cases**. This model is not necessarily directly informative because we are interested in estimating the magnitude of difference between means, even if that difference is small. But the model can be useful as a foil in model comparison. Moreover, it is (remotely) conceivable that ratings of the movies have an identical underlying mean, if, for example, the ratings were supplied by robots that did not actually watch the movies and instead gave random ratings from the same generating mean.

Because there is a single mean, the prior puts a multivariate normal on $\langle \mu, \log(\sigma_1), \log(\sigma_2), \theta_1, \theta_2 \rangle$.

7.1 Broad prior (Eqm Hetv model)

7.1.1 Determine prior constants (Eqm Hetv Model, broad prior)

The same numerical values are used as for the full model:

Code

```
##          mu logsigma[1] logsigma[2]   thresh[2]   thresh[3]
##       3.000       0.693       0.693       2.500       3.500
```

Code

```
##             mu logsigma[1] logsigma[2] thresh[2] thresh[3]
## mu           4        0.00        0.00         0         0
## logsigma[1]  0        0.48        0.00         0         0
## logsigma[2]  0        0.00        0.48         0         0
## thresh[2]    0        0.00        0.00         4         0
## thresh[3]    0        0.00        0.00         0         4
```

7.1.2 Prior predictive check (Eqm Hetv Model, broad prior)

The broad prior was specified abstractly to be symmetric on the response scale and to provide opportunity for extreme responses on either end of the scale. The broad prior is not intended to mimic any particular realistic response distribution. Nevertheless, a prior predictive check can at least verify the intended symmetry and extremity of the prior.

Code

Show prior predictions:

Code

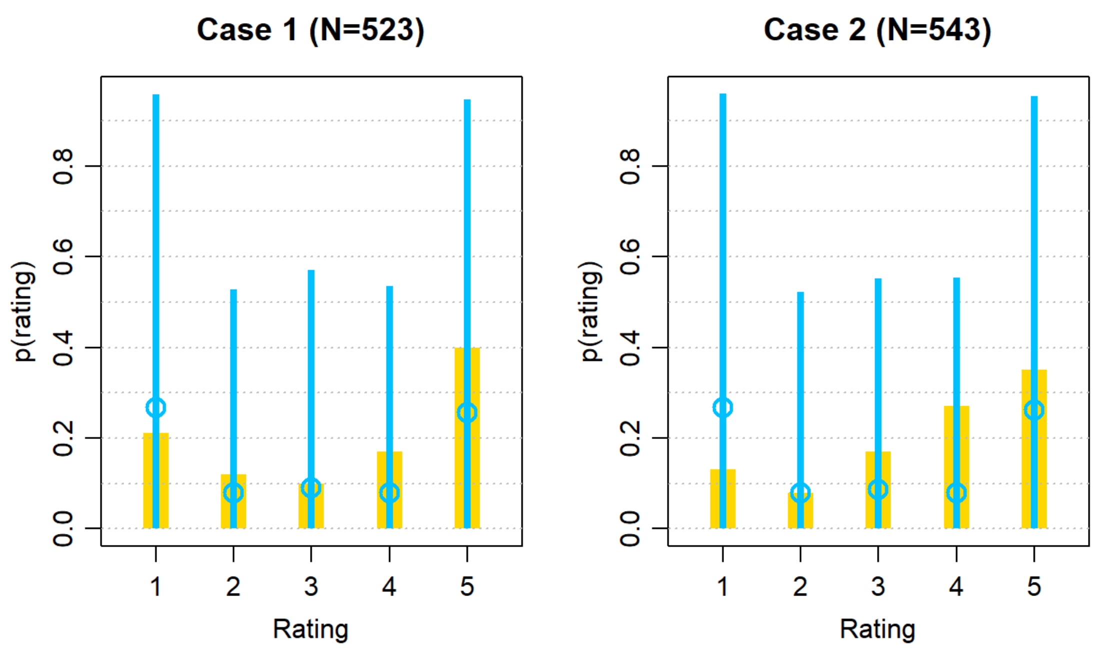

Above, it can be seen that the broad prior (blue dots are medians, bars are 95% ETI's) is indeed symmetric on the rating scale, and provides opportunity for either end of the scale to have a high probability.

7.1.3 Posterior MCMC diagnostics (Eqm Hetv Model, broad prior)

Code

Code

Show 10 entries

Search:

	psrfPt	psrfUpCI	ESS	50%	2.5%	97.5%	Mode	HDIlow	HDIhigh
mu	1	1	35845.286	3.76	3.607	3.916	3.757	3.606	3.915
sigma[1]	1	1.001	39357.269	2.876	2.576	3.234	2.858	2.563	3.217
sigma[2]	1	1	40624.204	1.953	1.78	2.152	1.948	1.772	2.141
thresh[2]	1	1.001	26429.29	2.314	2.195	2.44	2.315	2.193	2.438
thresh[3]	1.001	1.002	26672.666	3.195	3.068	3.319	3.204	3.073	3.323

Showing 1 to 5 of 5 entries

Previous 1 Next

Code

From the table above, we observe

- PSRF: The maximum PSRF of any parameter is 1.000592, indicating good MCMC convergence.
- ESS: The minimum ESS of any parameter is 26429.29, indicating stable estimates of limits of credible intervals.

7.1.4 Posterior predictive check (Eqm Hetv Model, broad prior)

Code

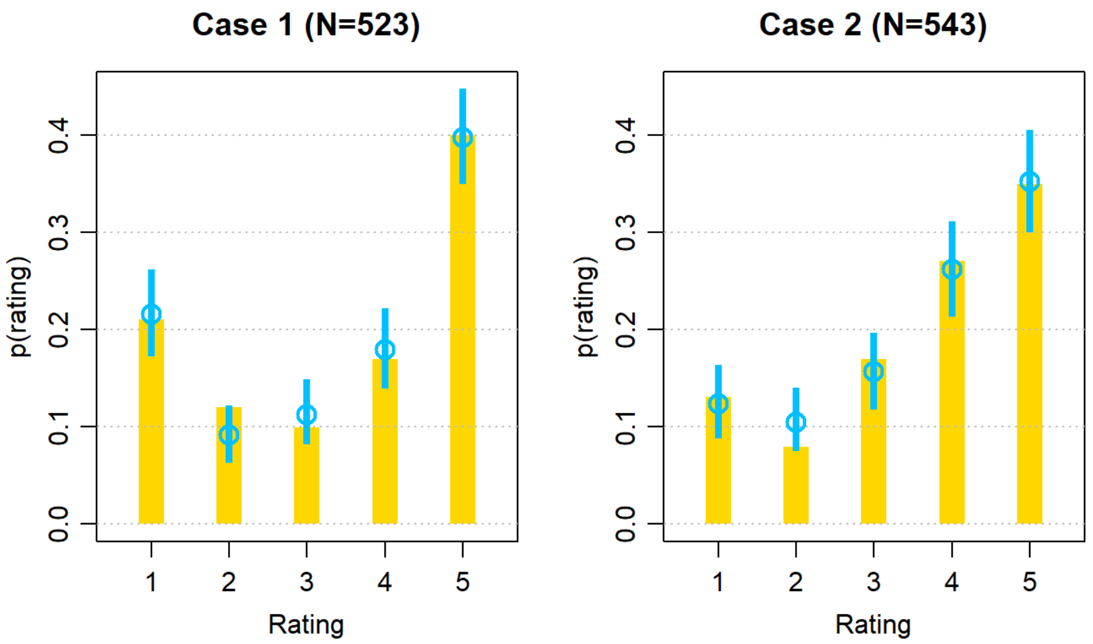

7.1.5 Posterior details (Eqm Hetv Model, broad prior)

Code

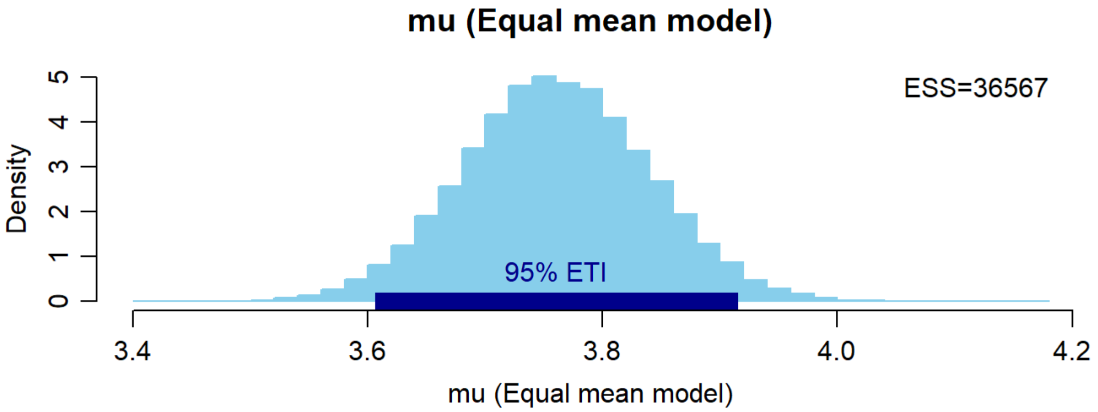

Code

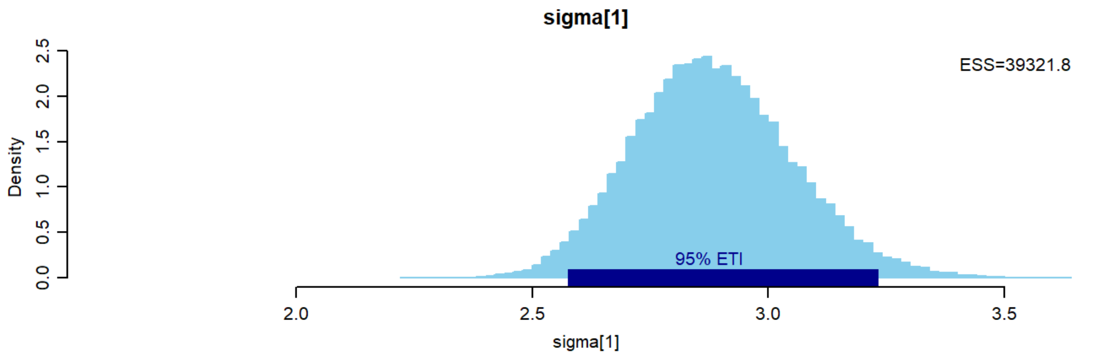

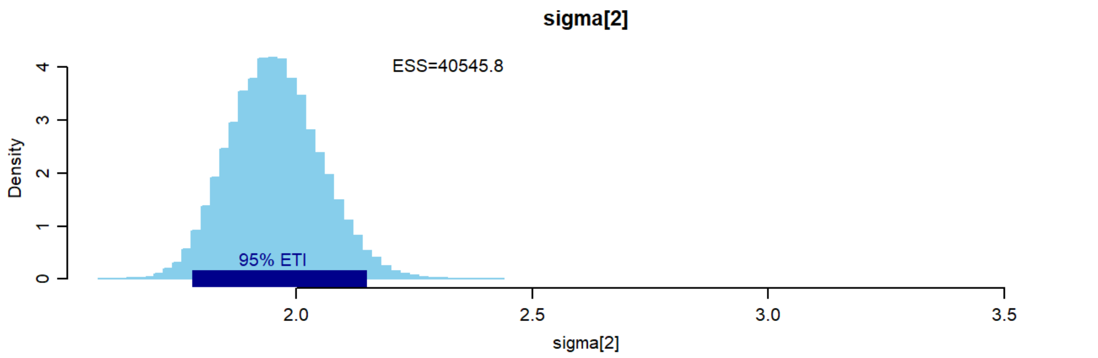

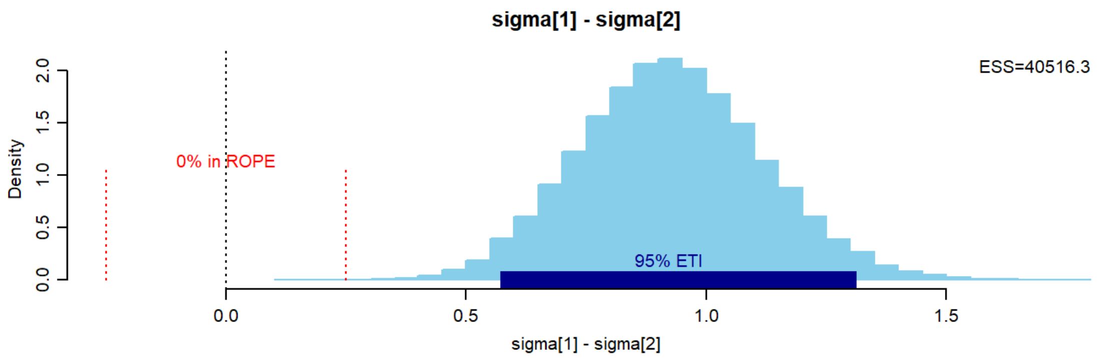

From the output above, it can be seen that the parameter estimates from the equal-mean heterogeneous-variance model are very similar to the estimates from the different-means heterogeneous variance model, which is not surprising because the two means estimated in the different-means model were very close to each other.

7.2 Prior informed by other cases (Eqm Hetv model)

The small representative data set was described earlier, the first time it was introduced.

7.2.1 Determine prior constants (Eqm Hetv model, informed by other)

Run the Eqm,Hetv model with the small representative data set, starting with the broad prior:

Code

MCMC Diagnostics:

Code

Show 10 **entries**

Search:

	psrfPt	psrfUpCI	ESS	50%	2.5%	97.5%	Mode	HDIlow	HDIhigh

	psrfPt	psrfUpCI	ESS	50%	2.5%	97.5%	Mode	HDIlow	HDIhigh
mu	1.001	1.002	14533.072	5.464	4.776	6.478	5.353	4.71	6.385
sigma[1]	1	1	17630.524	2.179	1.389	3.631	2.064	1.246	3.385
sigma[2]	1	1.001	16614.649	2.176	1.392	3.605	2.036	1.295	3.401
thresh[2]	1	1	17988.937	2.238	1.708	2.971	2.196	1.658	2.896
thresh[3]	1	1	18086.652	3.125	2.475	3.698	3.191	2.48	3.701

Showing 1 to 5 of 5 entries

Previous 1 Next

Code

From the table above, we observe

- PSRF: The maximum PSRF of any parameter is 1.00051, indicating good MCMC convergence.
- ESS: The minimum ESS of any parameter is 14533.07, indicating stable estimates of limits of credible intervals.

Posterior predictive check for prior data:

Code

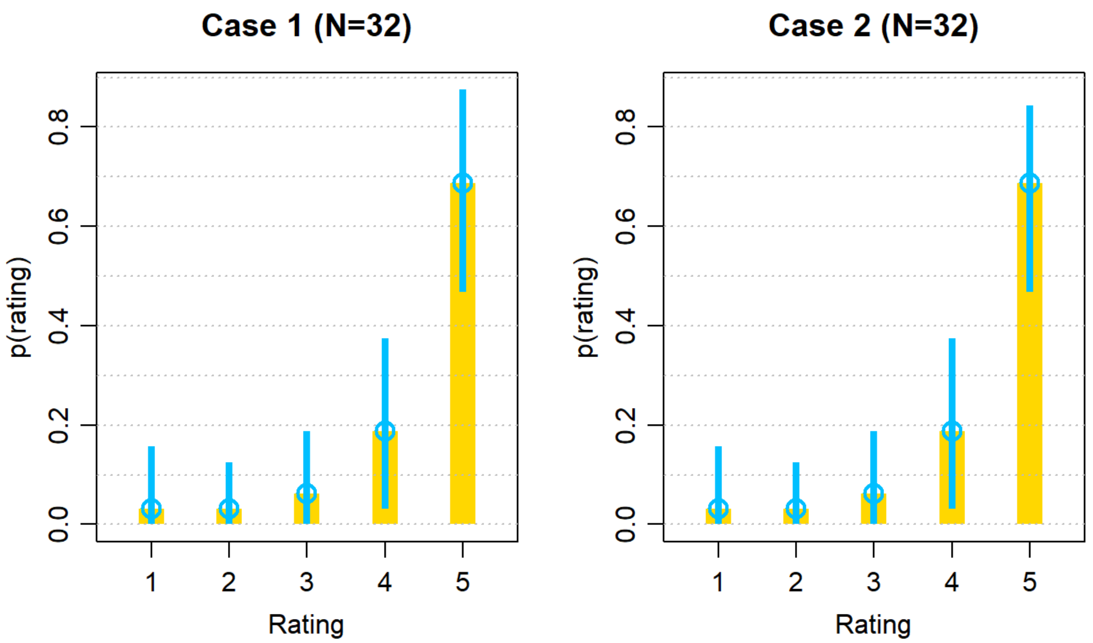

Above plot shows that the prior data are well represented by the model.

Now examine the posterior distribution. First, compute log(sigma):

Code

Pairs plots:

Code

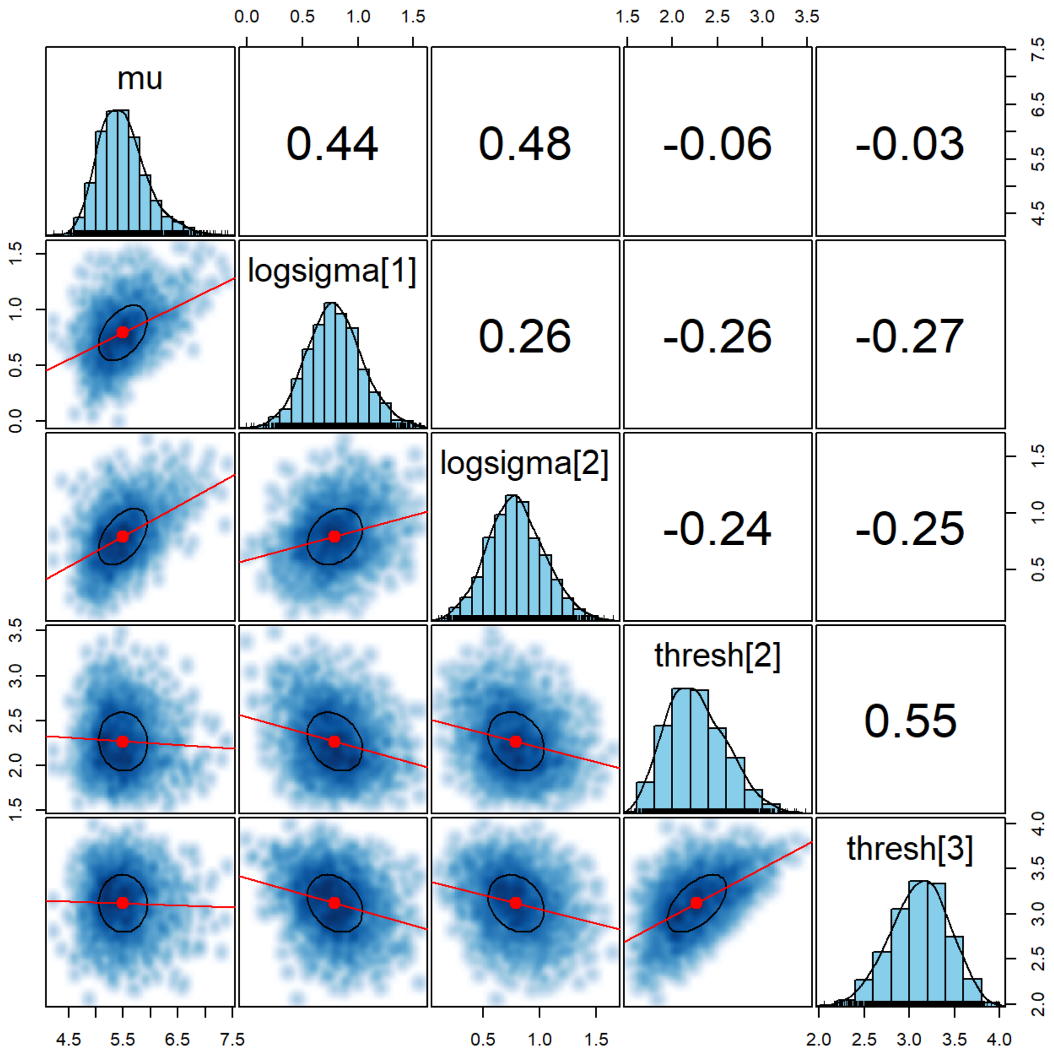

Here are the M and V,C constants used for the informed prior:

Code

```
##          mu logsigma[1] logsigma[2]   thresh[2]   thresh[3]
##       5.503       0.786       0.785       2.266       3.115
```

Code

```
##                 mu logsigma[1] logsigma[2] thresh[2] thresh[3]
## mu           0.189       0.049       0.049    -0.010    -0.008
## logsigma[1]  0.049       0.059       0.017    -0.019    -0.020
## logsigma[2]  0.049       0.017       0.059    -0.019    -0.020
## thresh[2]   -0.010      -0.019      -0.019     0.109     0.057
## thresh[3]   -0.008      -0.020      -0.020     0.057     0.099
```

7.2.2 Prior predictive check (Eqm Hetv model, informed by other)

If the mathematical prior were an exact copy of the posterior from the mildly informed fit, then a prior predictive check would be superfluous. But because the mathematical prior is merely a close approximation of the posterior from the mildly informed fit, a prior predictive check verifies that the prior really does mimic the small data as intended.

Code

Show prior predictions:

Code

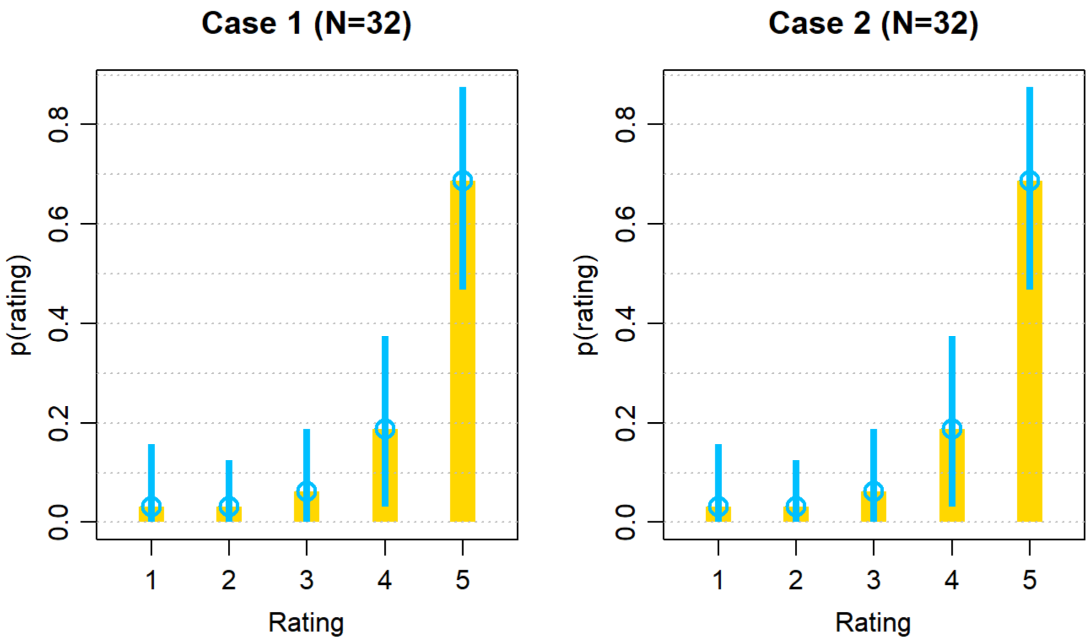

Above, it can be seen that the mildly-informed prior does mimic the small data.

7.2.3 Posterior MCMC diagnostics (Eqm Hetv model, informed by other)

Run Eqm, Hetv model on actual data with informed prior:

Code

Diagnostics:

Code

Show 10 **entries** **Search:**

	psrfPt	psrfUpCI	ESS	50%	2.5%	97.5%	Mode	HDIlow	HDIhigh
mu	1	1.001	36113.274	3.835	3.685	3.992	3.828	3.683	3.99
sigma[1]	1	1	41489.094	2.767	2.491	3.089	2.761	2.476	3.071
sigma[2]	1	1.001	37354.564	1.936	1.77	2.131	1.922	1.764	2.124
thresh[2]	1	1	26484.478	2.321	2.203	2.446	2.32	2.203	2.446
thresh[3]	1.001	1.001	25784.248	3.198	3.075	3.32	3.192	3.074	3.32

Showing 1 to 5 of 5 entries Previous 1 Next

Code

From the table above, we observe

- PSRF: The maximum PSRF of any parameter is 1.000576, indicating good MCMC convergence.

- ESS: The minimum ESS of any parameter is 25784.25, indicating stable estimates of limits of credible intervals.

7.2.4 Posterior predictive check (Eqm Hetv model, informed by other)

Code

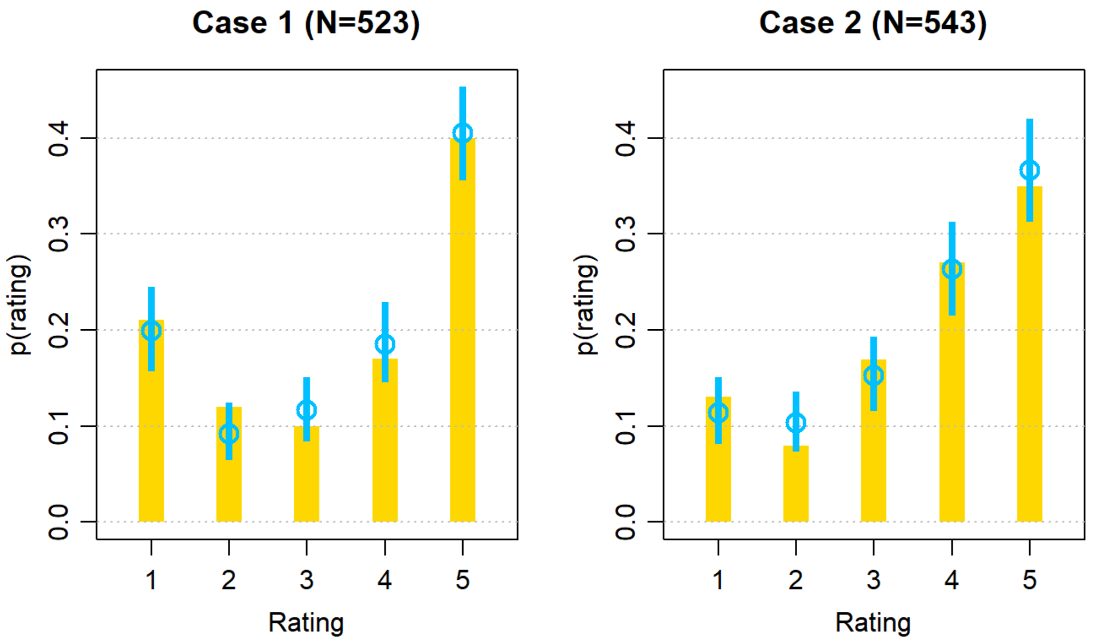

7.2.5 Posterior details (Eqm Hetv model, informed by other)

Posterior mu and sigma's:

Code

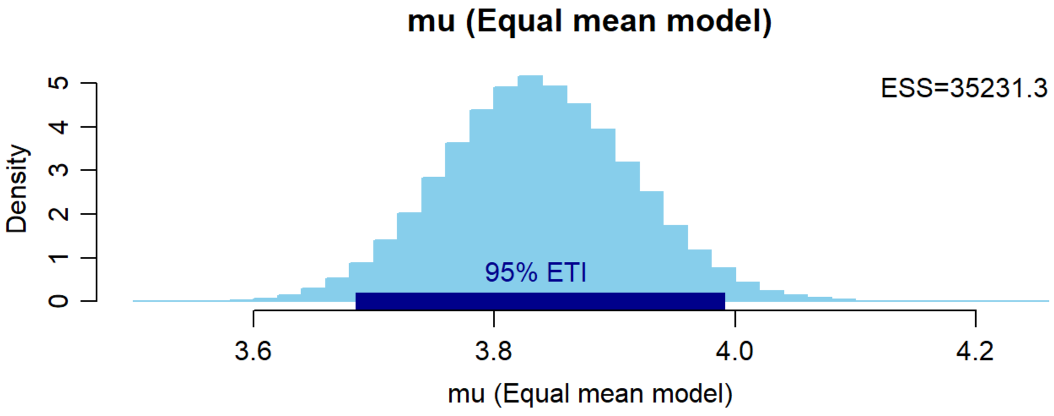

Code

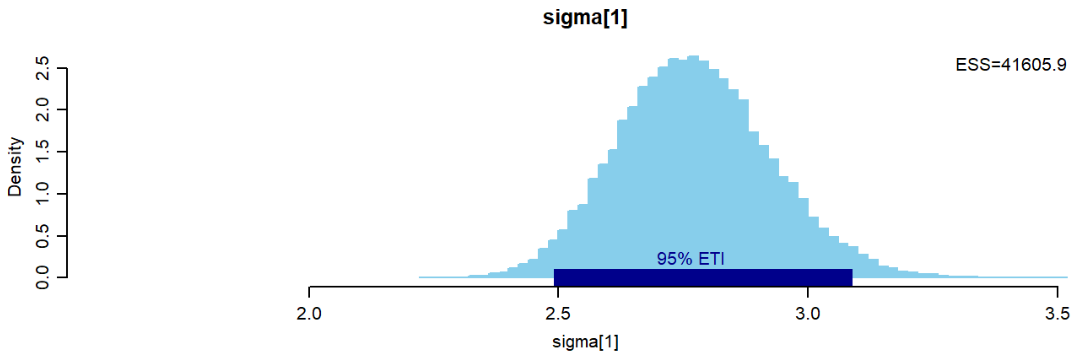

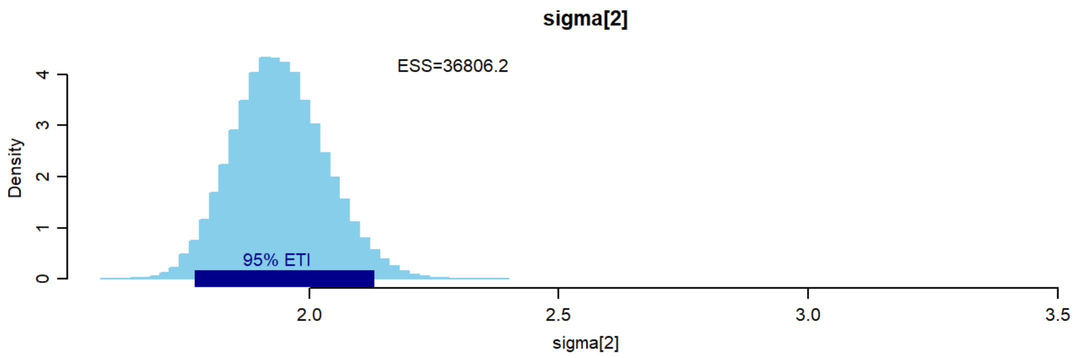

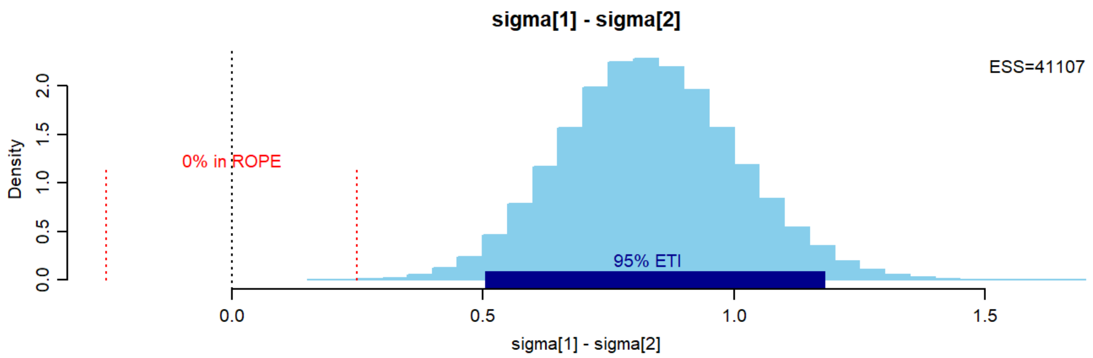

From the output above, it can be seen that the parameter estimates from the equal-mean heterogeneous-variance model are very similar to the estimates from the different-means heterogeneous-variance model, which is not surprising because the two means estimated in the different-means model were very close to each other (i.e., nearly equal).

7.3 Prior informed by subset of target data (Eqm Hetv model)

The representative subset was described above, the first time it was used.

7.3.1 Determine prior constants (Eqm Het model, prior informed by subset)

Run the model on the small representative data set using broad prior:

Code

MCMC diagnostics:

Code

Show 10 **entries** **Search:**

	psrfPt	psrfUpCI	ESS	50%	2.5%	97.5%	Mode	HDIlow	HDIhigh

	psrfPt	psrfUpCI	ESS	50%	2.5%	97.5%	Mode	HDIlow	HDIhigh
mu	1	1.001	27907.34	3.75	3.124	4.424	3.764	3.12	4.419
sigma[1]	1	1	30091.561	2.982	1.999	4.83	2.798	1.838	4.531
sigma[2]	1	1.001	29371.965	1.982	1.4	3.011	1.899	1.339	2.885
thresh[2]	1	1.001	20179.538	2.387	1.952	2.893	2.403	1.933	2.868
thresh[3]	1	1	19126.048	3.23	2.738	3.691	3.225	2.754	3.703

Showing 1 to 5 of 5 entries

Previous 1 Next

Code

From the table above, we observe

- PSRF: The maximum PSRF of any parameter is 1.000469, indicating good MCMC convergence.
- ESS: The minimum ESS of any parameter is 19126.05, indicating stable estimates of limits of credible intervals.

Posterior predictive check of small data:

Code

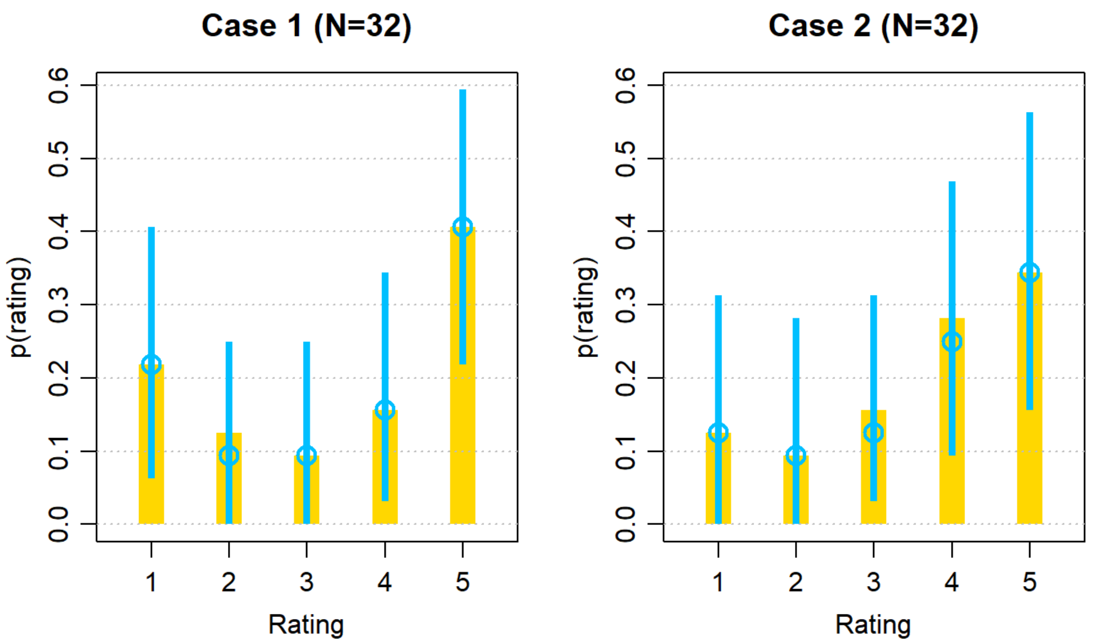

Above plot shows that the prior data are well represented by the model.

Now examine the posterior distribution (which will be used as the prior for the actual data). First, compute log(sigma):

Code

Pairs plots:

Code

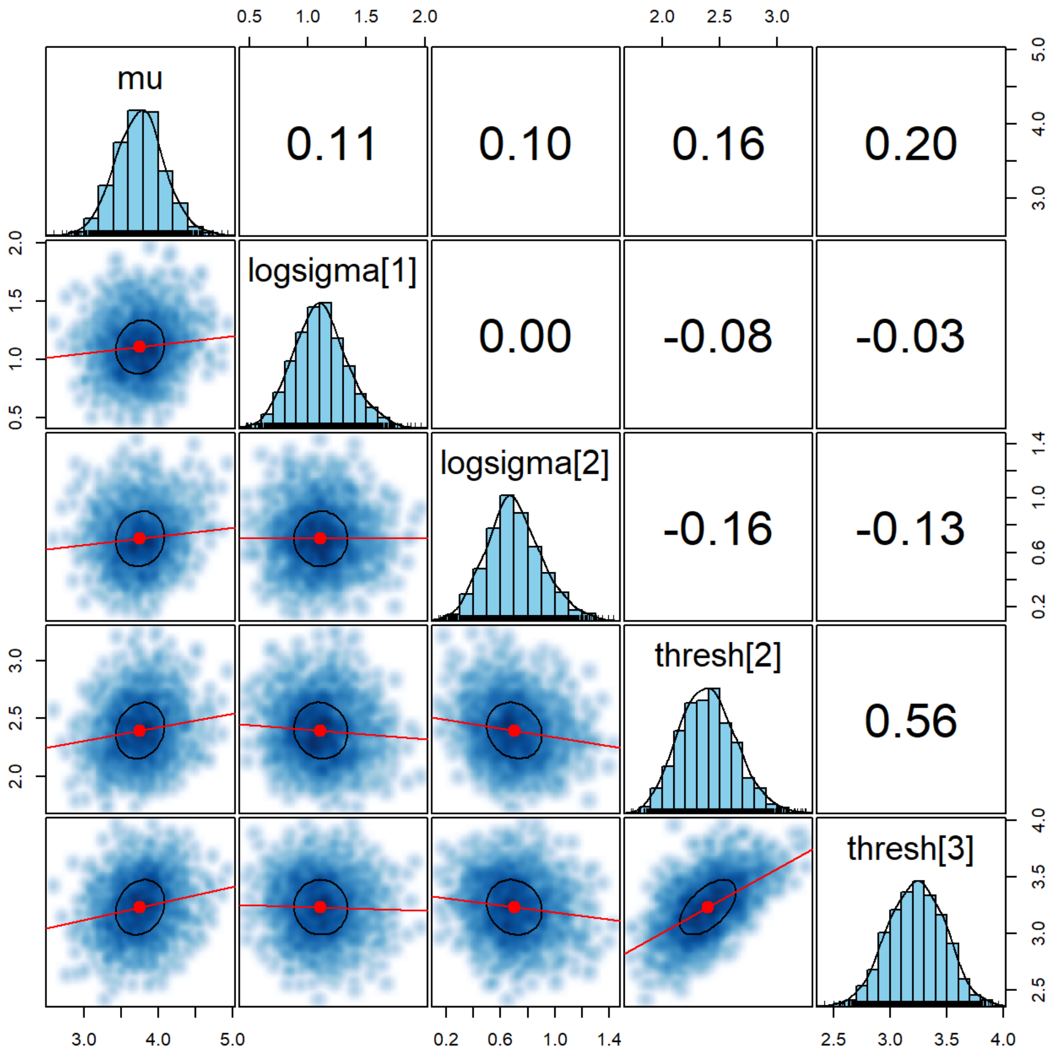

Here are the M, V, and C constants used for the informed prior:

Code

```
##          mu logsigma[1] logsigma[2]    thresh[2]    thresh[3]
##       3.756        1.104       0.692        2.396        3.225
```

Code

```
##                 mu logsigma[1] logsigma[2] thresh[2] thresh[3]
## mu           0.108       0.008       0.007     0.013     0.016
## logsigma[1] 0.008       0.051       0.001    -0.003    -0.002
## logsigma[2] 0.007       0.001       0.038    -0.006    -0.005
## thresh[2]   0.013      -0.003      -0.006     0.059     0.033
## thresh[3]   0.016      -0.002      -0.005     0.033     0.059
```

7.3.2 Prior predictive check (Eqm Het model, prior informed by subset)

If the mathematical prior were an exact copy of the posterior from the mildly informed fit, then a prior predictive check would be superfluous. But because the mathematical prior is merely a close approximation of the posterior from the mildly informed fit, a prior predictive check verifies that the prior really does mimic the small data as intended.

Code

Show prior predictions:

Code

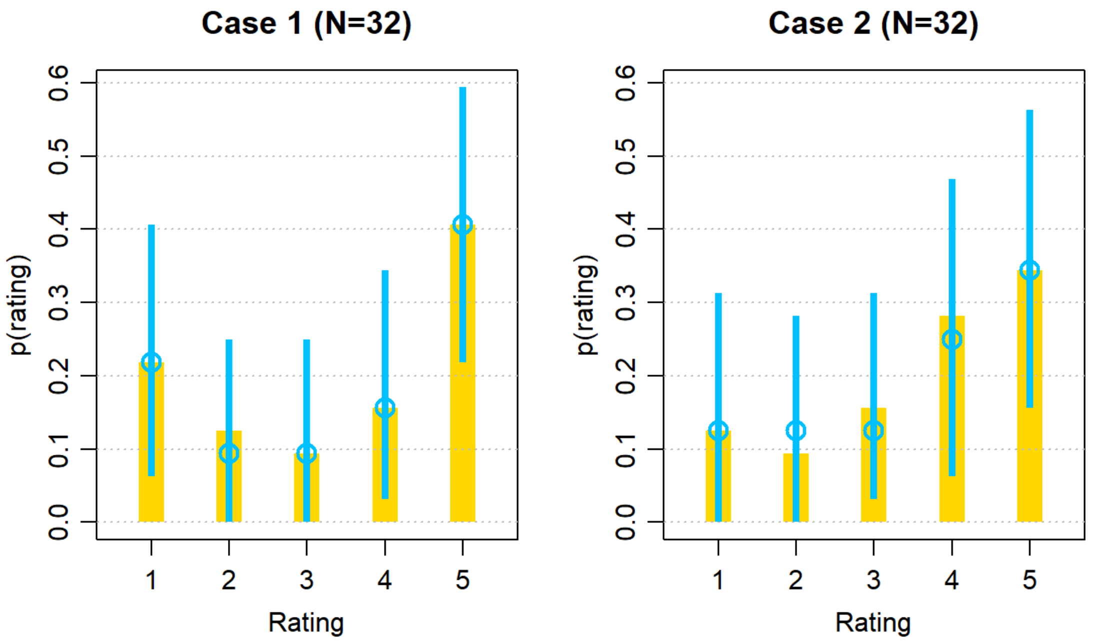

Above, it can be seen that the mildly-informed prior does mimic the small representative data.

7.3.3 Posterior MCMC diagnostics (Eqm Het model, prior informed by subset)

Run Eqm, Hetv model on actual data with informed prior:

Code

Diagnostics:

Code

Show 10 **entries** **Search:**

	psrfPt	psrfUpCI	ESS	50%	2.5%	97.5%	Mode	HDIlow	HDIhigh
mu	1	1	36686.128	3.761	3.613	3.916	3.756	3.614	3.917
sigma[1]	1	1.001	39511.847	2.891	2.596	3.238	2.873	2.588	3.23
sigma[2]	1	1	39486.429	1.956	1.786	2.152	1.958	1.779	2.142
thresh[2]	1	1	29091.317	2.319	2.203	2.442	2.318	2.202	2.441
thresh[3]	1	1.001	28006.703	3.197	3.073	3.317	3.197	3.072	3.316

Showing 1 to 5 of 5 entries Previous 1 Next

Code

From the table above, we observe

- PSRF: The maximum PSRF of any parameter is 1.000358, indicating good MCMC convergence.

- ESS: The minimum ESS of any parameter is 28006.7, indicating stable estimates of limits of credible intervals.

7.3.4 Posterior predictive check (Eqm Het model, prior informed by subset)

Code

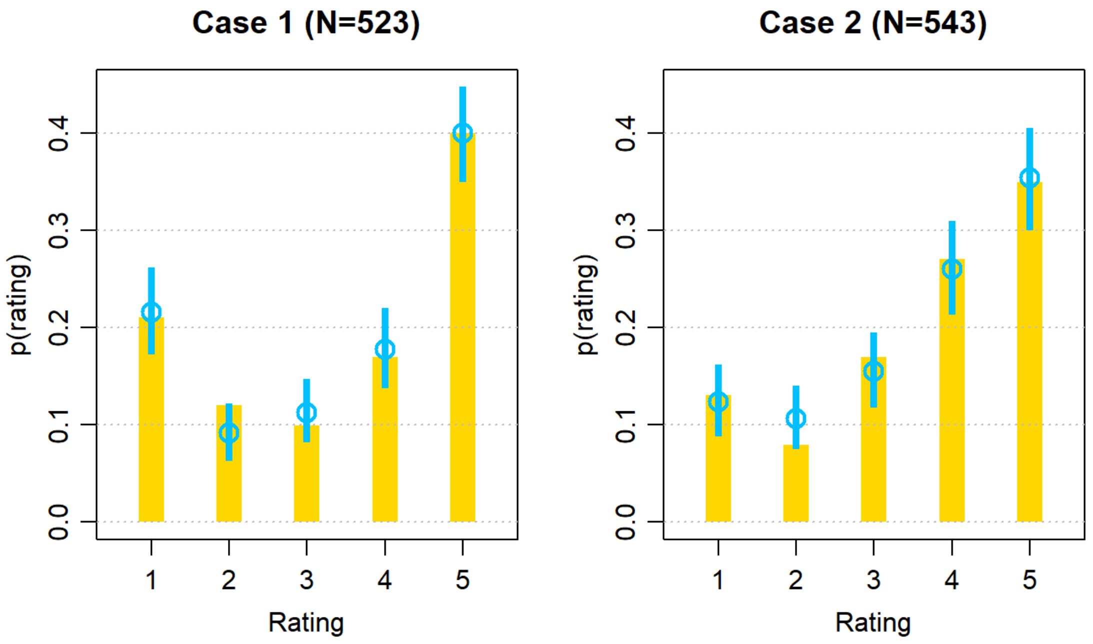

7.3.5 Posterior details (Eqm Het model, prior informed by subset)

Posterior mu and sigma's:

Code

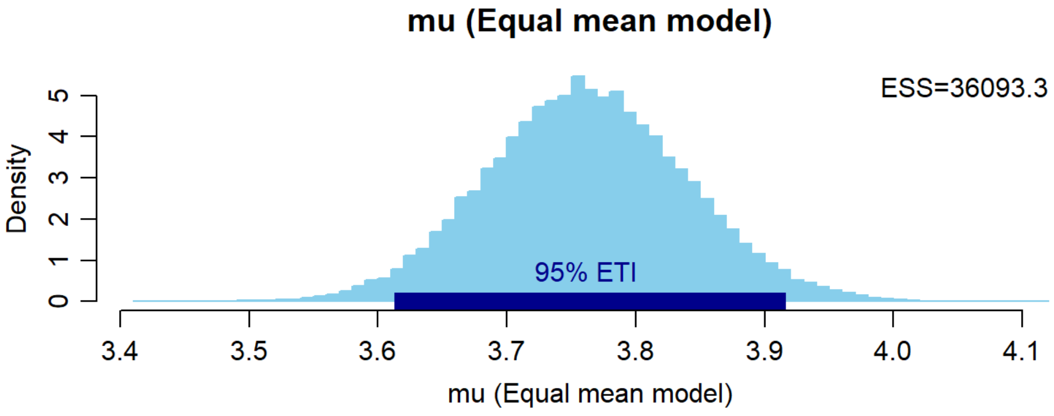

Code

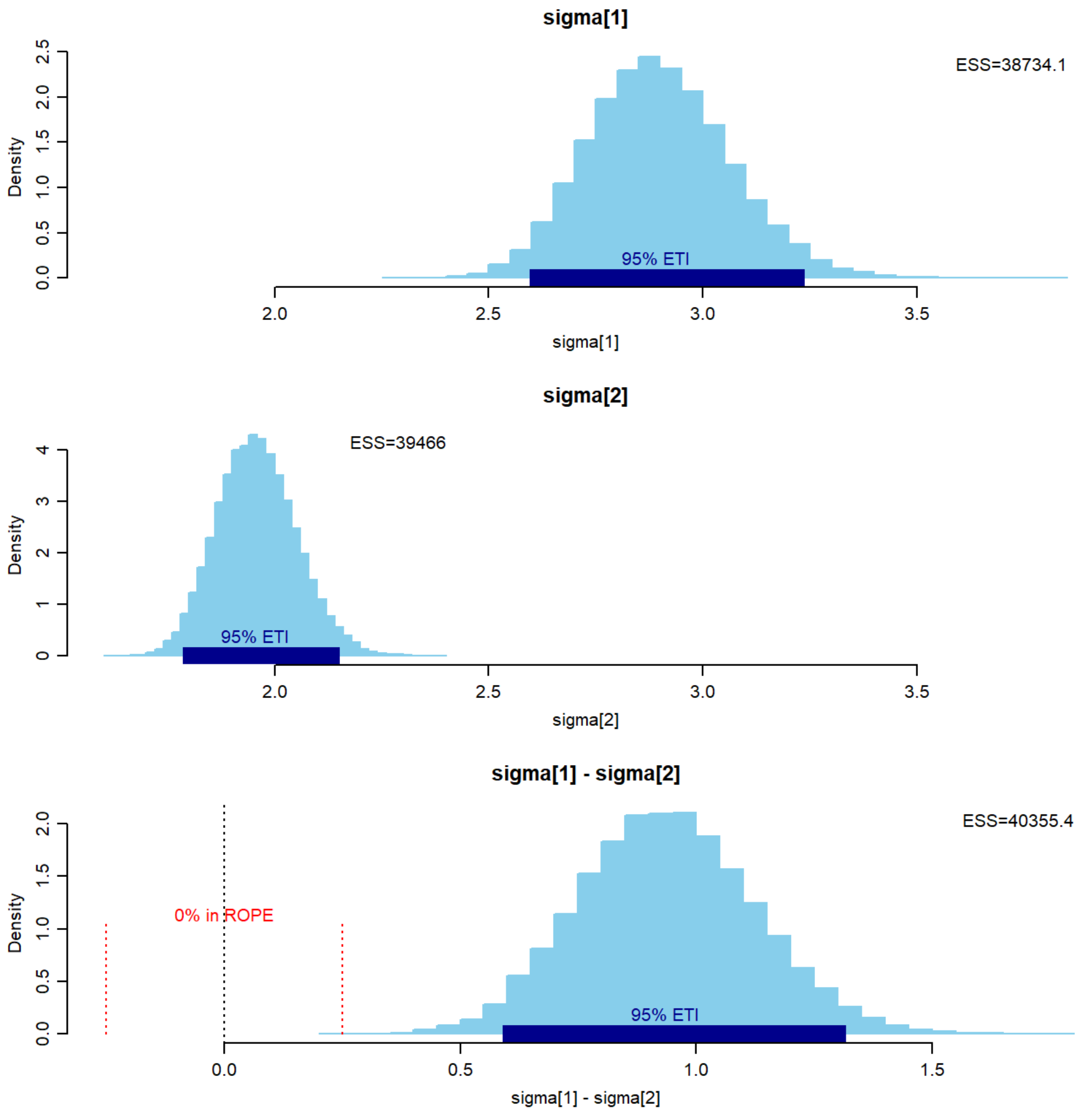

From the output above, it can be seen that the parameter estimates from the equal-mean heterogeneous-variance model are very similar to the estimates from the different-means heterogeneous variance model, which is not surprising because the two means estimated in the different-means model were very close to each other.

7.4 Sensitivity of Eqm, Hetv model to prior

Below are graphical comparisons of the marginal posteriors from the three priors. The plot of posterior density relies on smoothing by a kernel with width determined heuristically. The posterior density is a natural way to view the mode and HDI. The plot of cumulative probability does not rely on smoothing (but is effectively smoothed by the pixel resolution). The cumulative probability is a natural way to view the median and ETI.

Code

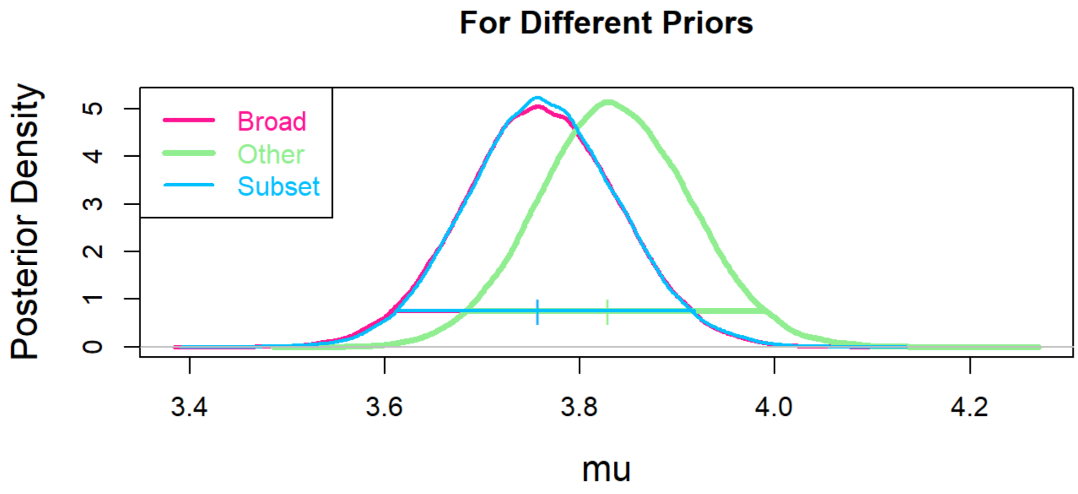
For Different Priors
Posterior Density
Broad
Other
Subset
0
1
2
3
4
5
3.4
3.6
3.8
4.0
4.2
mu

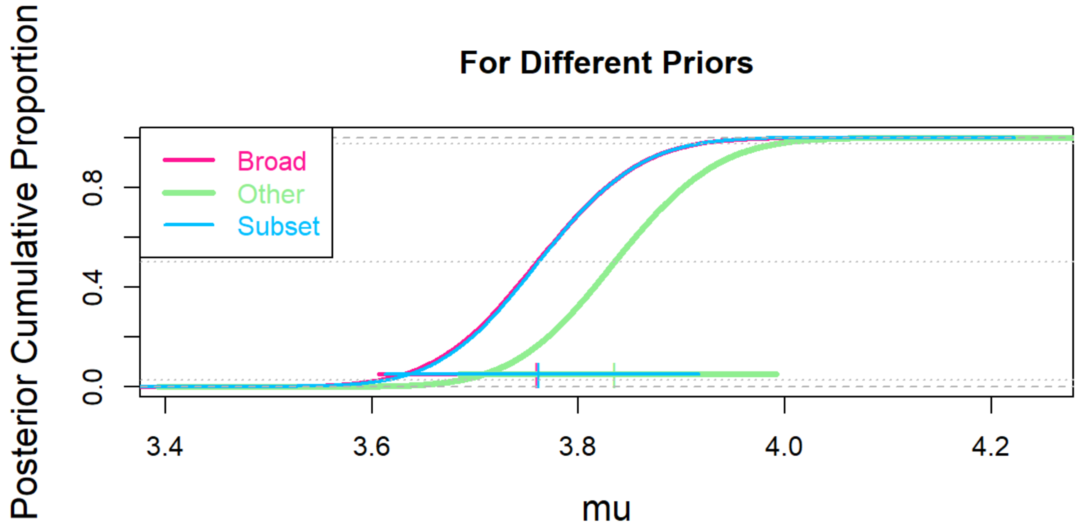
For Different Priors
Posterior Cumulative Proportion
Broad
Other
Subset
0.0
0.4
0.8
3.4
3.6
3.8
4.0
4.2
mu

Code

Code

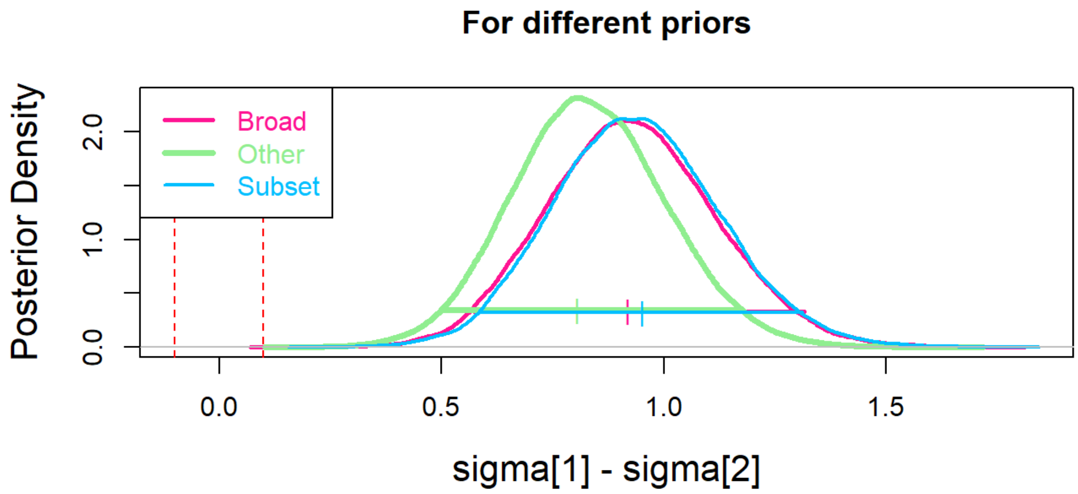

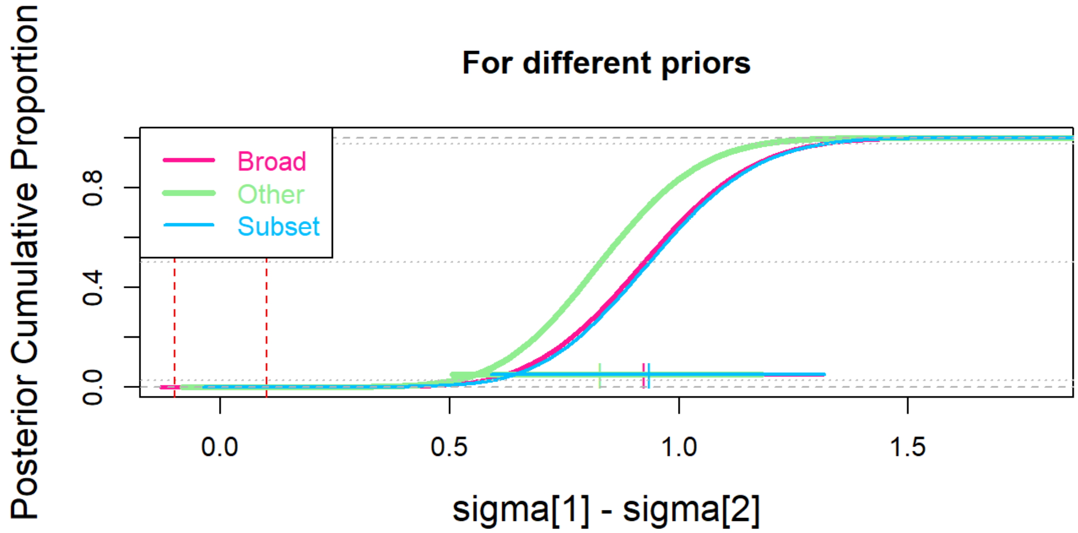

Code

The impact of the different priors is made clear by the plot, but the magnitude of difference relative to the credible intervals and relative to the ROPE is very small. That is, the conclusion remains the same as the full model that there is a notable non-zero difference of standard deviations.

8 Restricted Model: Different means, homogeneous variances

This model allows different means but restricts there to be **a single variance used simultaneously for all cases**. This model is not necessarily directly informative because we are interested in estimating the magnitude of difference between variances, even if that difference is small. But the model can be useful as a foil in model comparison, and an assumption of homogeneous variances is typical in conventional ordered-probit models. Moreover, it is (remotely) conceivable that ratings of the movies have an identical underlying variance, if, for example, the ratings were supplied by robots that did not actually watch the movies and instead gave random ratings from the same generating distributions.

Because there is a single variance, the prior puts a multivariate normal on $\langle \mu_1, \mu_2, \log(\sigma), \theta_1, \theta_2 \rangle$.

8.1 Broad prior (Diffm Homv model)

8.1.1 Determine prior constants (Diffm Homv Model, broad prior)

The same numerical values are used as for the full model:

Code

```
##     mu[1]     mu[2]  logsigma thresh[2] thresh[3]
##     3.000     3.000     0.693     2.500     3.500
```

Code

```
##           mu[1] mu[2] logsigma thresh[2] thresh[3]
## mu[1]         4     0     0.00         0         0
## mu[2]         0     4     0.00         0         0
## logsigma      0     0     0.48         0         0
## thresh[2]     0     0     0.00         4         0
## thresh[3]     0     0     0.00         0         4
```

8.1.2 Prior predictive check (Diffm Homv Model, broad prior)

The broad prior was specified abstractly to be symmetric on the response scale and to provide opportunity for extreme responses on either end of the scale. The broad prior is not intended to mimic any particular realistic response distribution. Nevertheless, a prior predictive check can at least verify the intended symmetry and extremity of the prior.

Code

Show prior predictions:

Code

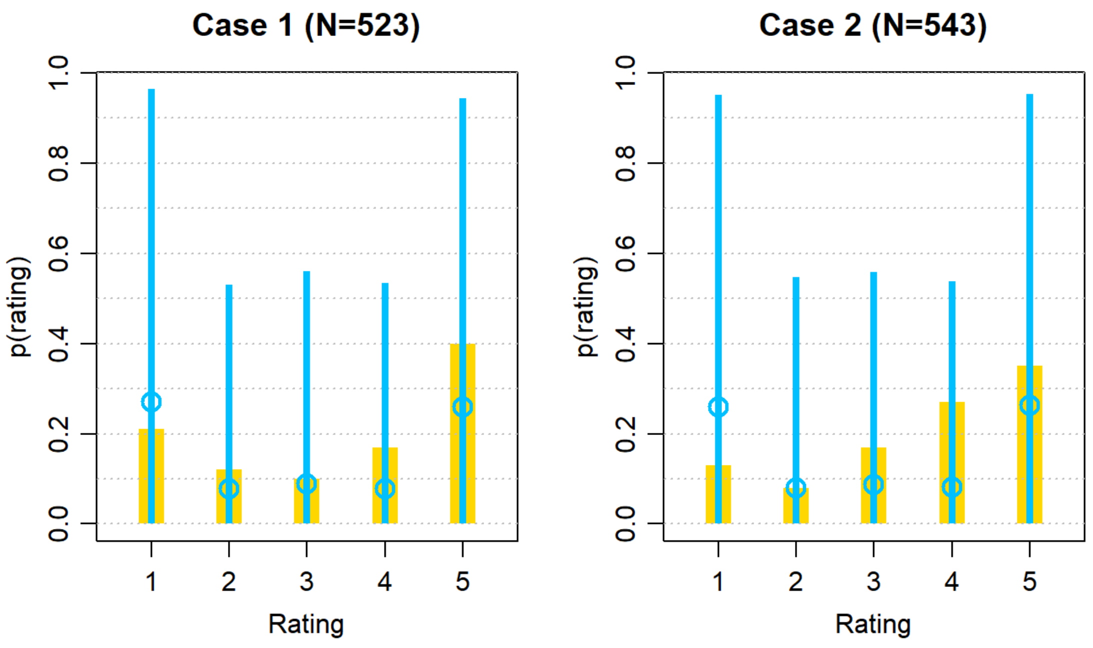

Above, it can be seen that the broad prior (blue dots are medians, bars are 95% ETI's) is indeed symmetric on the rating scale, and provides opportunity for either end of the scale to have a high probability.

8.1.3 Posterior MCMC diagnostics (Diffm Homv Model, broad prior)

Code

Code

Show 10 entries Search:

	psrfPt	psrfUpCI	ESS	50%	2.5%	97.5%	Mode	HDIlow	HDIhigh
mu[1]	1	1	40339.674	3.638	3.413	3.866	3.647	3.411	3.863
mu[2]	1	1.001	39166.576	3.846	3.627	4.068	3.851	3.627	4.068
sigma	1	1	38354.895	2.354	2.188	2.54	2.345	2.181	2.533
thresh[2]	1	1	26926.115	2.301	2.182	2.427	2.295	2.18	2.424
thresh[3]	1	1.001	27655.432	3.182	3.055	3.308	3.186	3.058	3.311

Showing 1 to 5 of 5 entries

Previous 1 Next

Code

From the table above, we observe

- PSRF: The maximum PSRF of any parameter is 1.00024, indicating good MCMC convergence.
- ESS: The minimum ESS of any parameter is 26926.11, indicating stable estimates of limits of credible intervals.

8.1.4 Posterior predictive check (Diffm Homv Model, broad prior)

Code

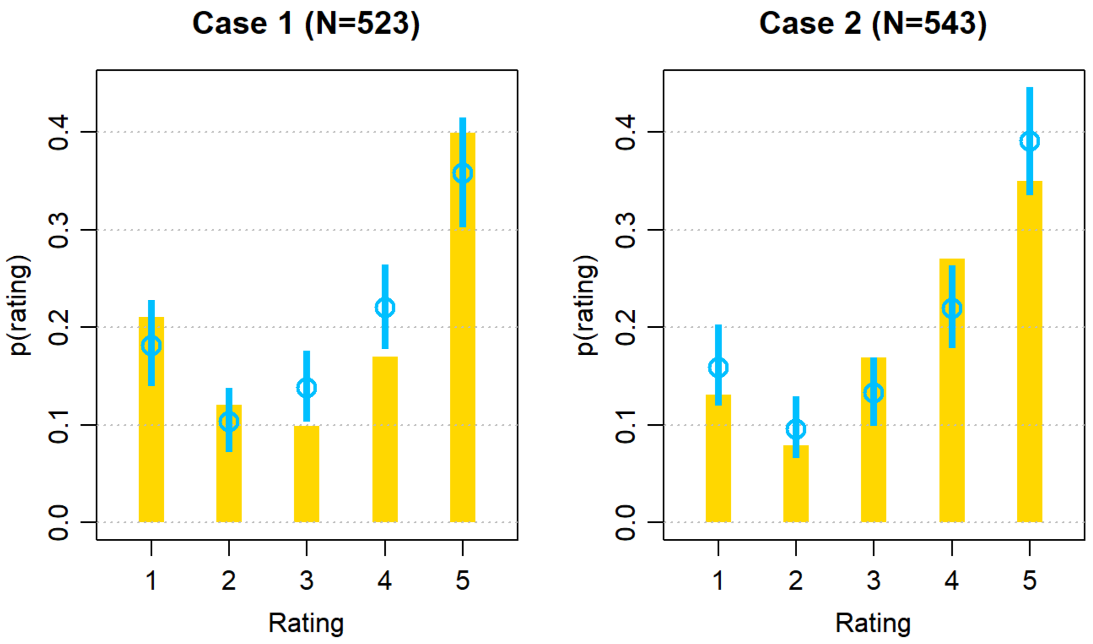

It can be seen above that the Diffm,Homv model does *not* mimic the data very well, or at least not nearly as well as the heterogenous-variance models. This is not surprising, given that the heterogeneous-variance models estimated a large difference between variances, so this homogeneous-variance model should strain to fit the data.

8.1.5 Posterior details (Diffm Homv Model, broad prior)

The poor fit should make us suspicious of interpreting the parameter values. But merely for completeness, here they are.

Difference of means:

Code

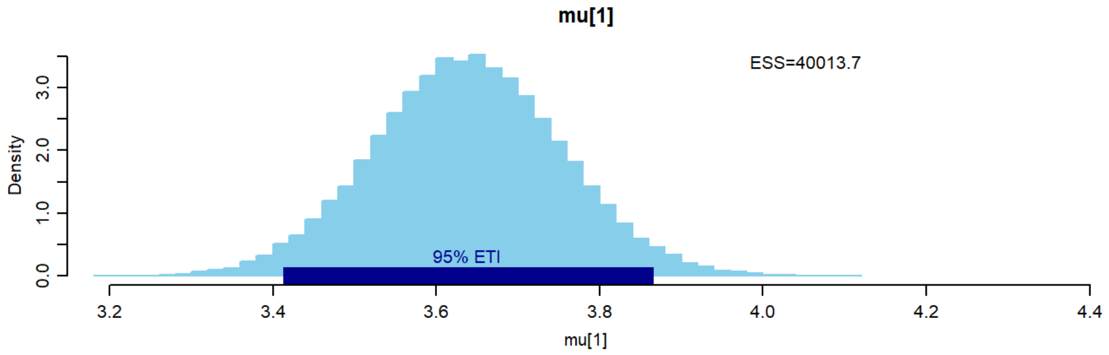

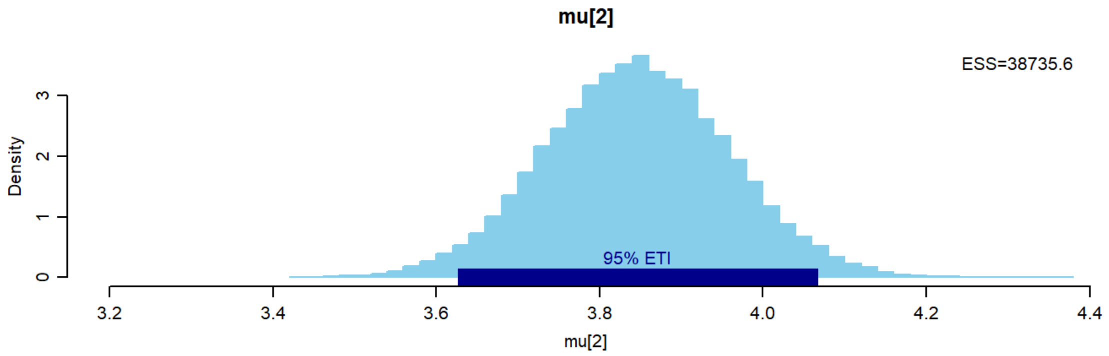

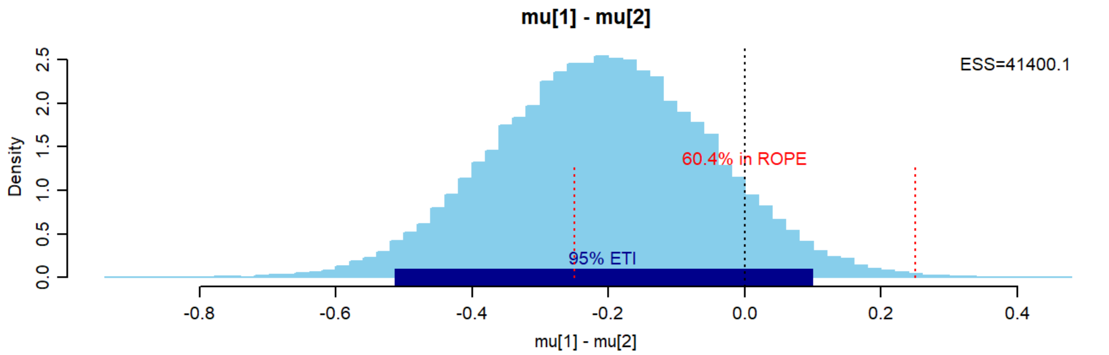

Effect size (Cohen's d), $(\mu_1 - \mu_2)\Big/\sqrt{(\sigma_1^2 + \sigma_2^2)/2}$:

Code

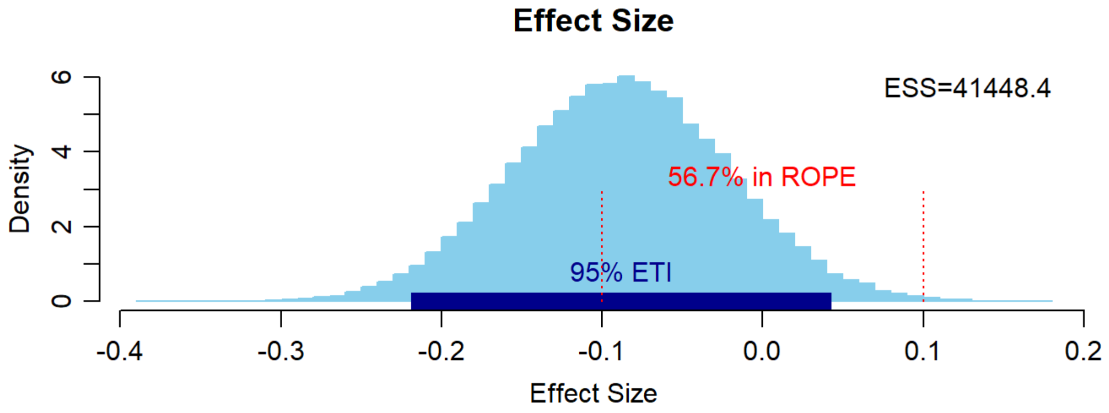

Code

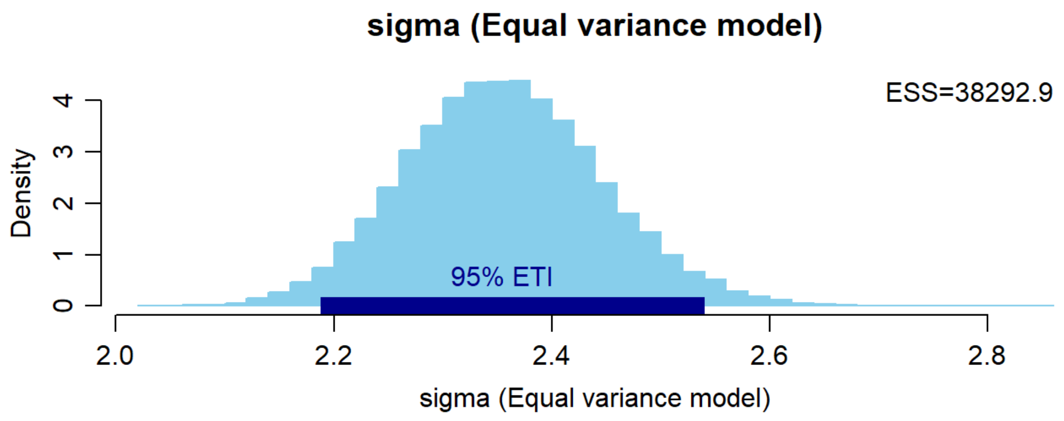

Again, the estimated parameter values above should not be taken too seriously, given the relatively poor fit of the model.

8.2 Prior informed by other cases (Diffm Homv model)

The small representative data set was described earlier, the first time it was introduced.

8.2.1 Determine prior constants (Diffm Homv model, informed by other)

Run the Diffm,Homv model with the small representative data set, starting with the broad prior:

Code

MCMC Diagnostics:

Code

Show 10 **entries** **Search:**

	psrfPt	psrfUpCI	ESS	50%	2.5%	97.5%	Mode	HDIlow	HDIhigh
mu[1]	1	1	19514.76	5.433	4.536	6.643	5.329	4.467	6.554
mu[2]	1	1	20750.164	5.428	4.535	6.642	5.355	4.462	6.551
sigma	1	1	13881.738	2.185	1.503	3.265	2.077	1.434	3.143
thresh[2]	1	1	17561.389	2.22	1.698	2.948	2.157	1.652	2.882
thresh[3]	1	1.001	16804.052	3.104	2.445	3.688	3.144	2.459	3.7

Showing 1 to 5 of 5 entries Previous 1 Next

Code

From the table above, we observe

- PSRF: The maximum PSRF of any parameter is 1.000174, indicating good MCMC convergence.
- ESS: The minimum ESS of any parameter is 13881.74, indicating stable estimates of limits of credible intervals.

Posterior predictive check for prior data:

Code

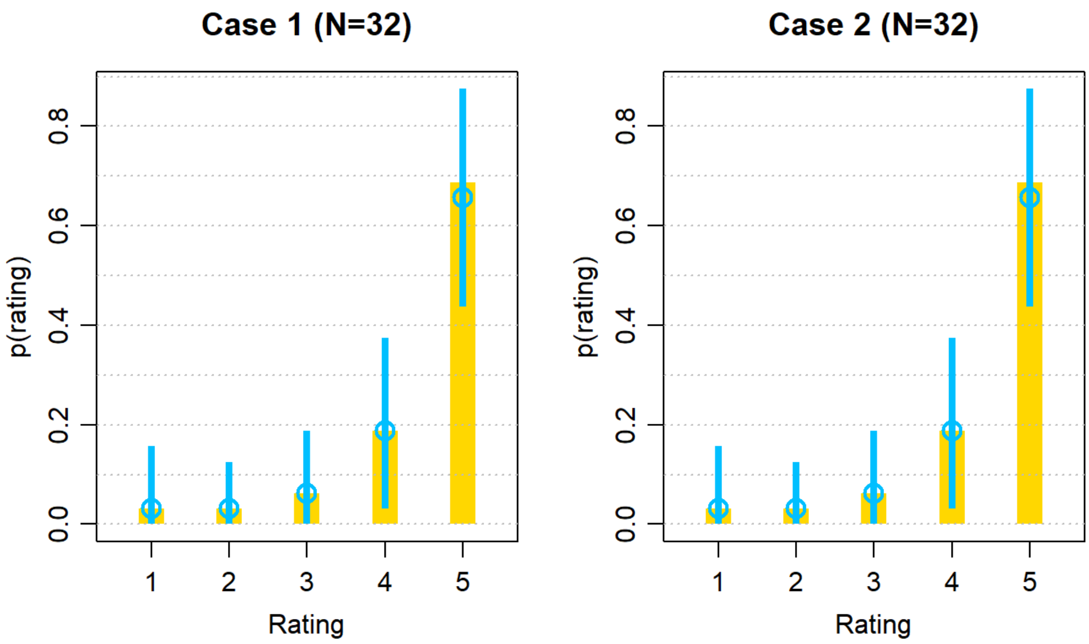

Above plot shows that the prior data are well represented by the model.

Now examine the posterior distribution. First, compute log(sigma):

Code

Pairs plots:

Code

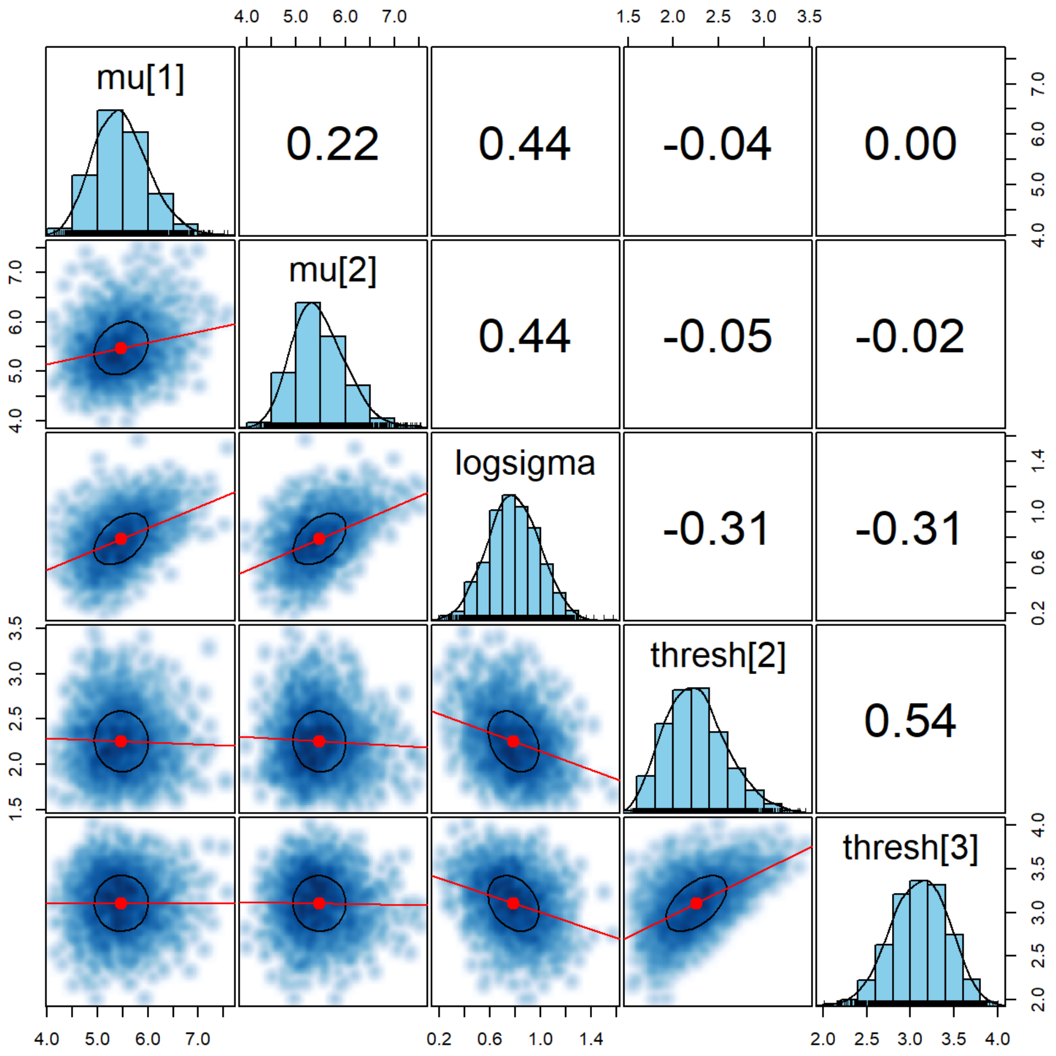

Here are the M and V,C constants used for the informed prior:

Code

```
##     mu[1]     mu[2]  logsigma thresh[2] thresh[3]
##     5.475     5.472     0.785     2.246     3.093
```

Code

```
##             mu[1]  mu[2] logsigma thresh[2] thresh[3]
## mu[1]       0.290  0.062    0.047    -0.010    -0.006
## mu[2]       0.062  0.289    0.046    -0.009    -0.007
## logsigma    0.047  0.046    0.039    -0.020    -0.021
## thresh[2] -0.010 -0.009   -0.020     0.108     0.058
## thresh[3] -0.006 -0.007   -0.021     0.058     0.102
```

8.2.2 Prior predictive check (Diffm Homv model, informed by other)

If the mathematical prior were an exact copy of the posterior from the mildly informed fit, then a prior predictive check would be superfluous. But because the mathematical prior is merely a close approximation of the posterior from the mildly informed fit, a prior predictive check verifies that the prior really does mimic the small data as intended.

Code

Show prior predictions:

Code

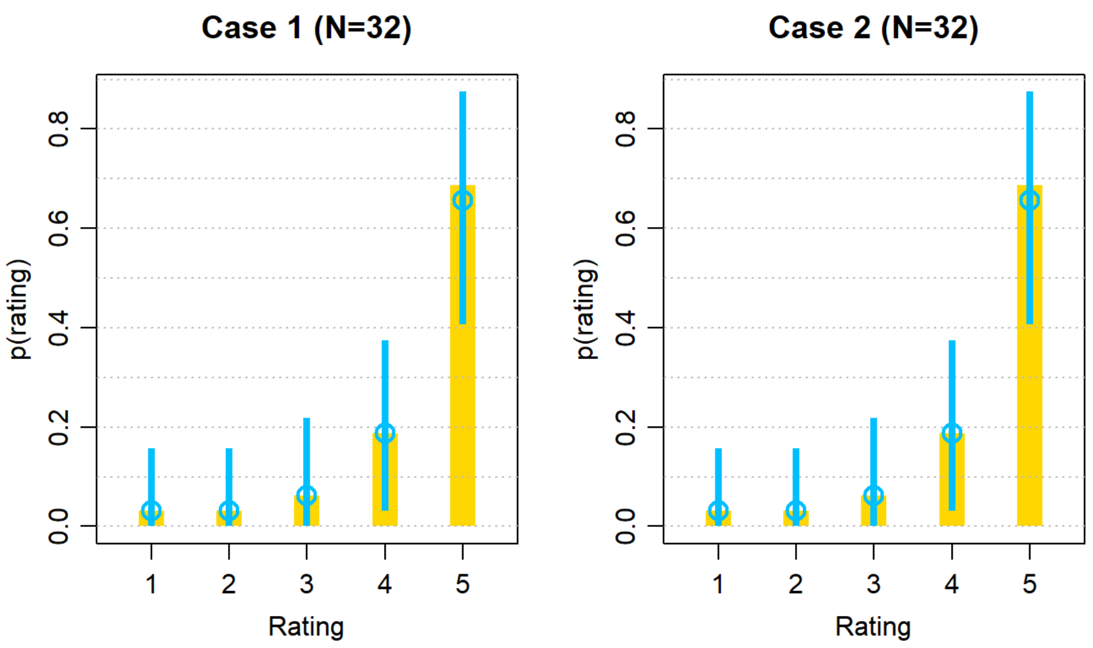

Above, it can be seen that the mildly-informed prior does mimic the small data.

8.2.3 Posterior MCMC diagnostics (Diffm Homv model, informed by other)

Run Diffm, Homv model on actual data with informed prior:

Code

Diagnostics:

Code

Show 10 **entries** **Search:**

	psrfPt	psrfUpCI	ESS	50%	2.5%	97.5%	Mode	HDIlow	HDIhigh
mu[1]	1	1	38176.594	3.73	3.516	3.953	3.724	3.511	3.948
mu[2]	1	1	38169.978	3.922	3.712	4.14	3.92	3.709	4.136
sigma	1	1.001	33950.431	2.31	2.149	2.485	2.311	2.145	2.481
thresh[2]	1	1	28619.642	2.308	2.191	2.431	2.307	2.191	2.43
thresh[3]	1	1.001	27535.421	3.185	3.06	3.308	3.189	3.06	3.307

Showing 1 to 5 of 5 entries

Previous 1 Next

Code

From the table above, we observe

- PSRF: The maximum PSRF of any parameter is 1.000198, indicating good MCMC convergence.

- ESS: The minimum ESS of any parameter is 27535.42, indicating stable estimates of limits of credible intervals.

8.2.4 Posterior predictive check (Diffm Homv model, informed by other)

Code

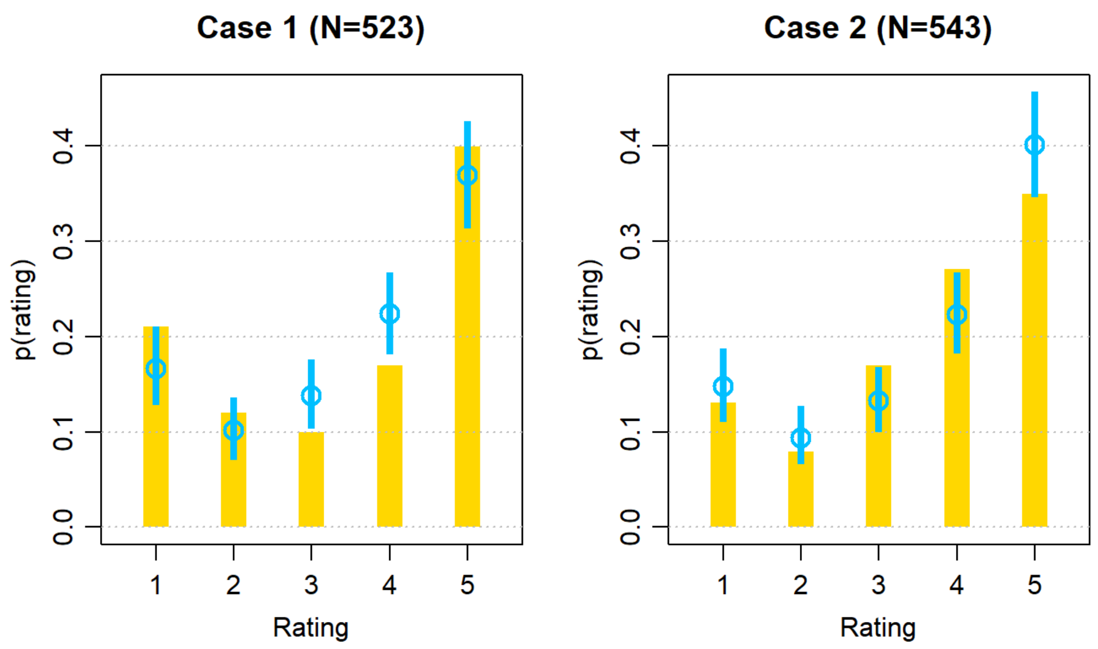

The above plot shows a poor fit by the homogeneous-variance model, at least much less accurate than the heterogeneous-variance models.

8.2.5 Posterior details (Diffm Homv model, informed by other)

Despite the poor fit of the model, the parameter estimates are displayed here for completeness:

Difference of means:

Code

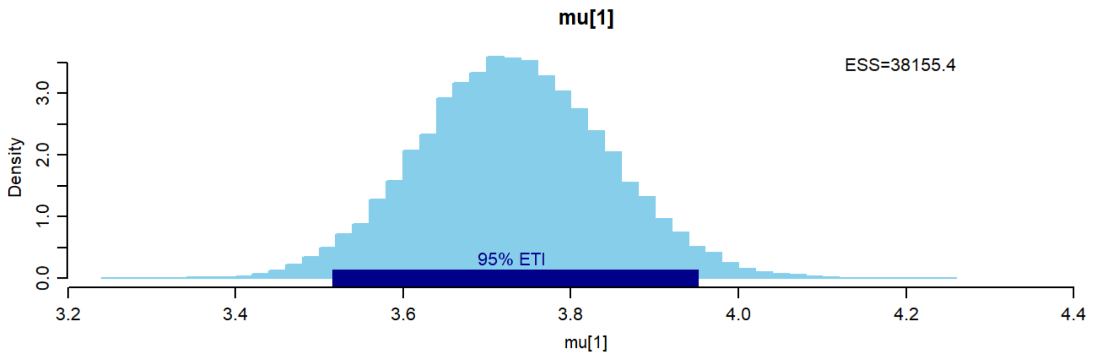

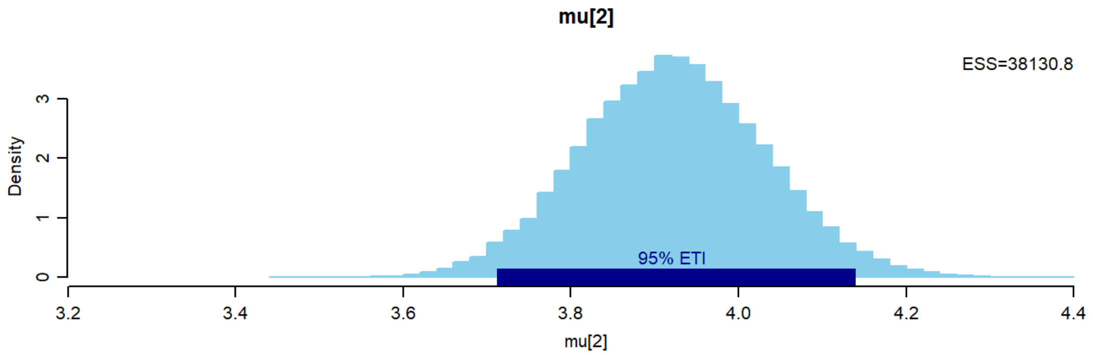

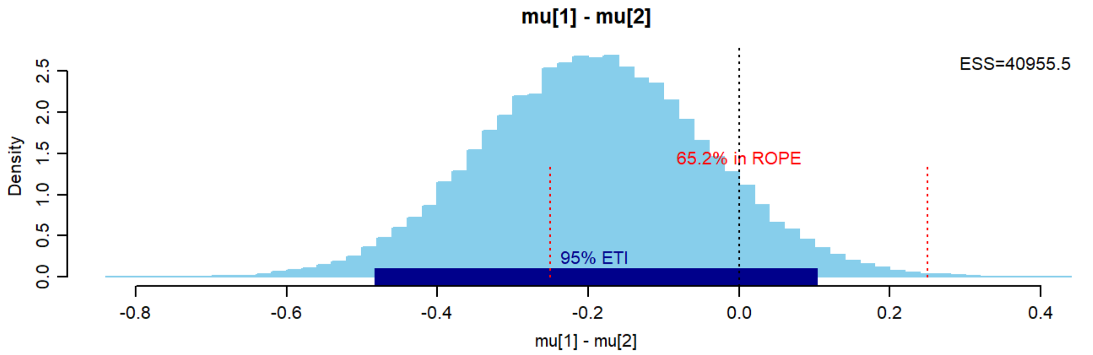

Effect size (Cohen's d), $(\mu_1 - \mu_2) \Big/ \sqrt{(\sigma_1^2 + \sigma_2^2)/2}$:

Code

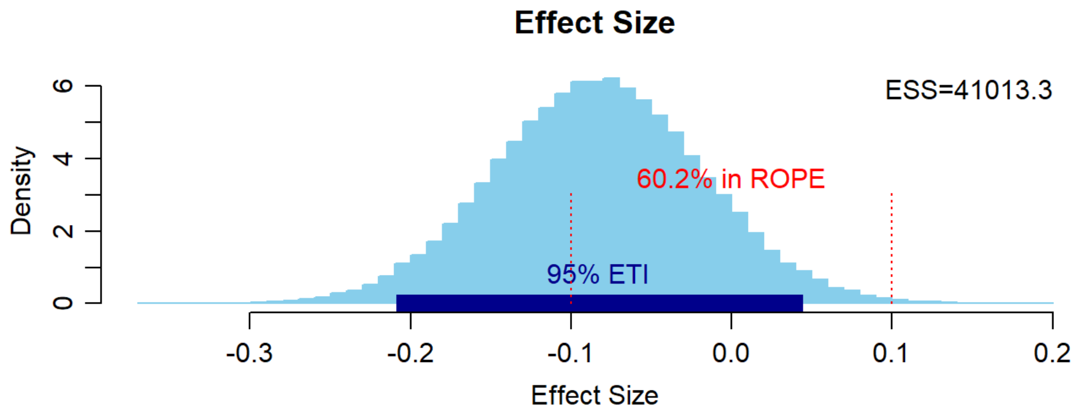

Code

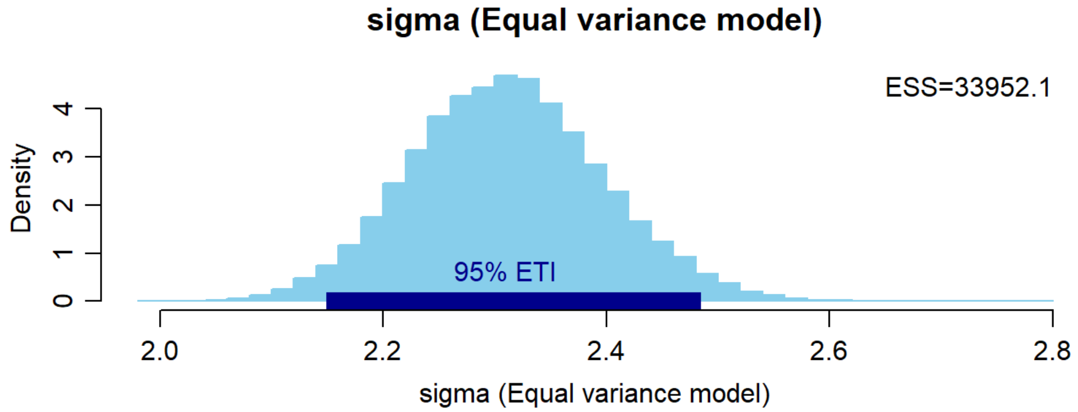

Again, the estimated parameter values above should not be taken too seriously, given the relatively poor fit of the model.

8.3 Prior informed by subset of target data (Diffm Homv model)

The representative subset was described above, the first time it was used.

8.3.1 Determine prior constants (Diffm Homv model, prior informed by subset)

Run the model on the small representative data set using broad prior:

Code

MCMC diagnostics:

Code

Show 10 **entries** **Search:**

	psrfPt	psrfUpCI	ESS	50%	2.5%	97.5%	Mode	HDIlow	HDIhigh
mu[1]	1	1	29906.286	3.619	2.675	4.619	3.568	2.65	4.592
mu[2]	1	1	29648.659	3.834	2.922	4.82	3.793	2.889	4.776
sigma	1	1.001	24664.854	2.478	1.867	3.43	2.376	1.801	3.325
thresh[2]	1	1	19786.423	2.363	1.939	2.869	2.338	1.922	2.846
thresh[3]	1	1	19462.603	3.21	2.713	3.677	3.177	2.735	3.697

Showing 1 to 5 of 5 entries

Previous 1 Next

Code

From the table above, we observe

- PSRF: The maximum PSRF of any parameter is 1.000182, indicating good MCMC convergence.
- ESS: The minimum ESS of any parameter is 19462.6, indicating stable estimates of limits of credible intervals.

Posterior predictive check of small data:

Code

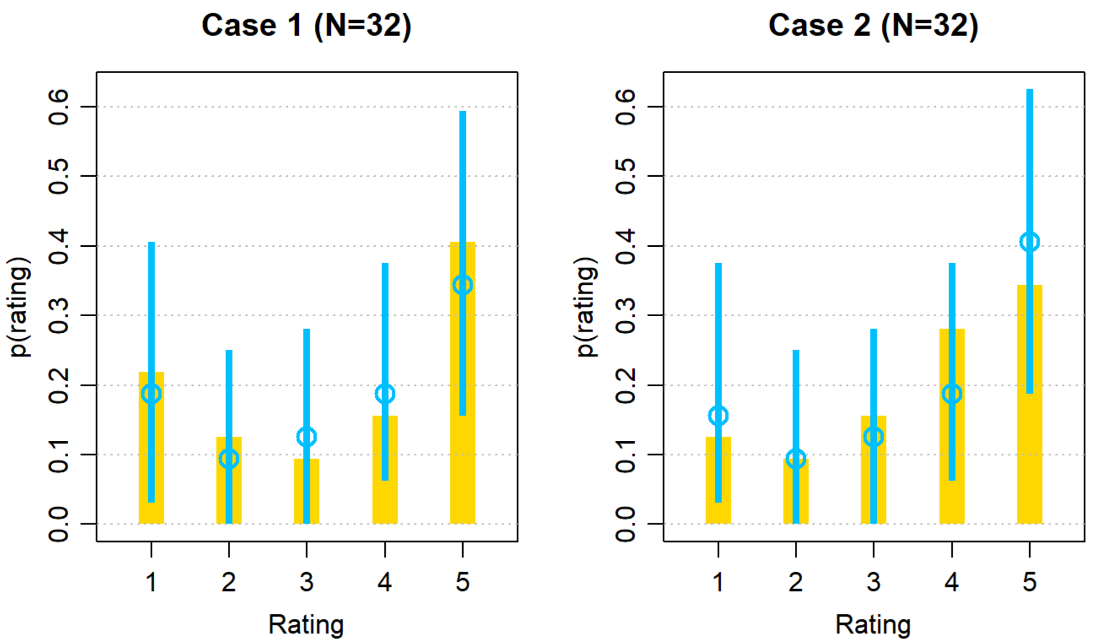

Above plot shows that the prior data are not very well represented by the model, but at least the data are within the broad uncertainty bands of the prior. This lack of fit by the homogeneous model should not be too surprising given that the prior data are designed to represent the full data with their heterogeneous variances. *Nevertheless we proceed with this prior because it is the best the model can do to mimic the representative subset data.*

Now examine the posterior distribution (which will be used as the prior for the actual data). First, compute log(sigma):

Code

Pairs plots:

Code

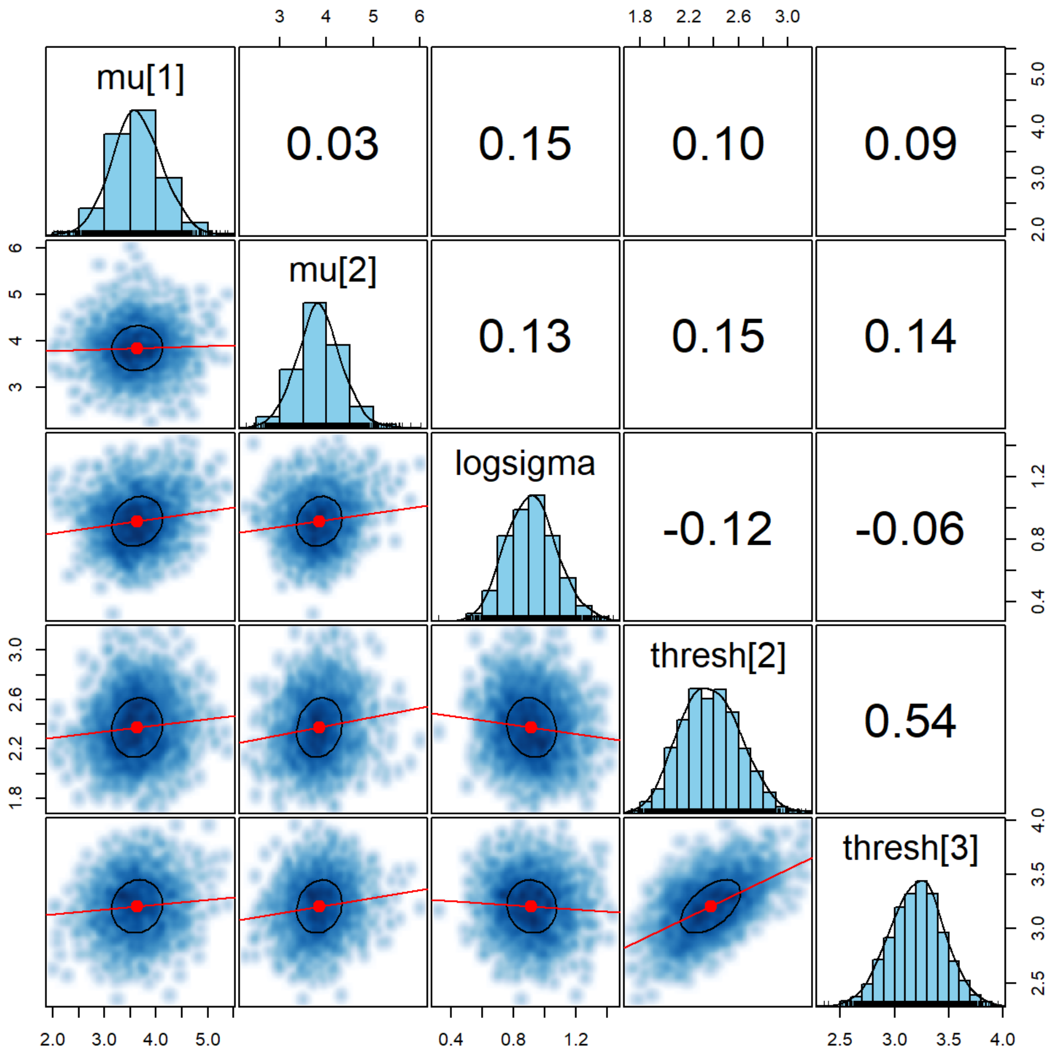

Here are the M, V, and C constants used for the informed prior:

Code

```
##     mu[1]     mu[2]  logsigma thresh[2] thresh[3]
##     3.627     3.845     0.913     2.374     3.206
```

Code

```
##           mu[1] mu[2] logsigma thresh[2] thresh[3]
## mu[1]     0.239 0.007    0.007     0.011     0.011
## mu[2]     0.007 0.231    0.009     0.013     0.016
## logsigma  0.007 0.009    0.024    -0.005    -0.003
## thresh[2] 0.011 0.013   -0.005     0.057     0.032
## thresh[3] 0.011 0.016   -0.003     0.032     0.061
```

8.3.2 Prior predictive check (Diffm Homv model, prior informed by subset)

If the mathematical prior were an exact copy of the posterior from the mildly informed fit, then a prior predictive check would be superfluous. But because the mathematical prior is merely a close approximation of the posterior from the mildly informed fit, a prior predictive check is useful.

Code

Show prior predictions:

Code

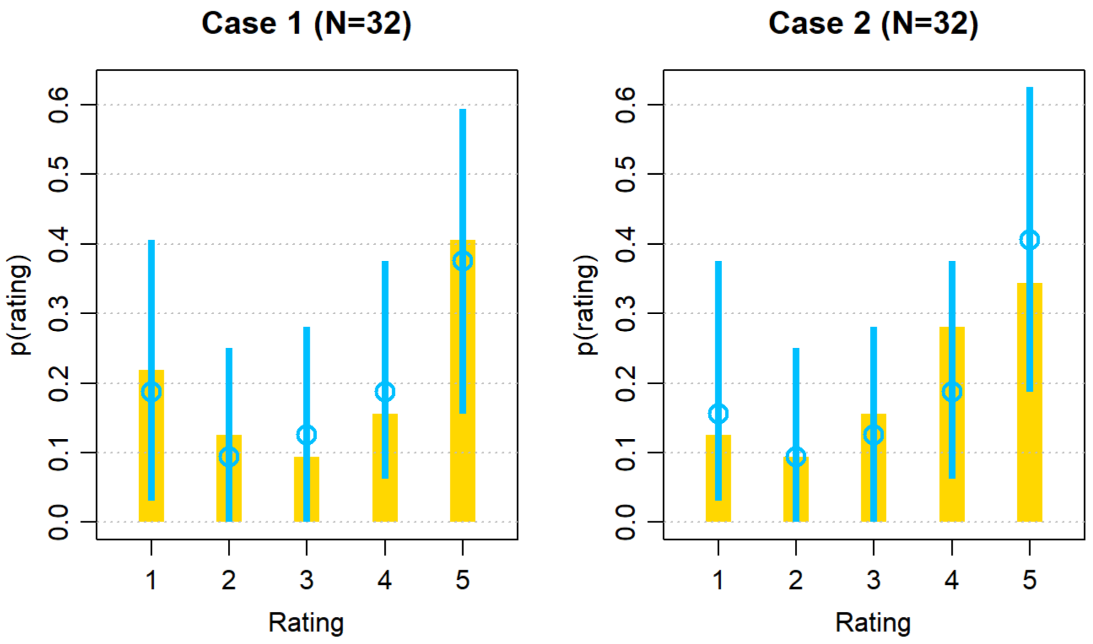

The mathematically specified prior does mimic the MCMC prior, which was the goal of this demonstration. But, as noted before, the prior for the homogeneous-variance model cannot capture the subset-data very well because it seems to have different variances. We proceed because this is, nevertheless, the best the prior distribution can do to capture the prior data.

8.3.3 Posterior MCMC diagnostics (Diffm Homb model, prior informed by subset)

Run Diffm, Homv model on actual data with informed prior:

Code

Diagnostics:

Code

Show 10 **entries** **Search:**

	psrfPt	psrfUpCI	ESS	50%	2.5%	97.5%	Mode	HDIlow	HDIhigh
mu[1]	1	1	38757.518	3.64	3.422	3.864	3.634	3.419	3.86
mu[2]	1	1	39186.637	3.849	3.635	4.067	3.834	3.63	4.061
sigma	1	1	38957.511	2.363	2.2	2.542	2.36	2.197	2.54
thresh[2]	1	1.001	28055.148	2.306	2.189	2.426	2.308	2.189	2.425
thresh[3]	1	1	28197.021	3.182	3.059	3.305	3.182	3.056	3.301

Showing 1 to 5 of 5 entries

Previous 1 Next

Code

From the table above, we observe

- PSRF: The maximum PSRF of any parameter is 1.000131, indicating good MCMC convergence.
- ESS: The minimum ESS of any parameter is 28055.15, indicating stable estimates of limits of credible intervals.

8.3.4 Posterior predictive check (Diffm Homv model, prior informed by subset)

Code

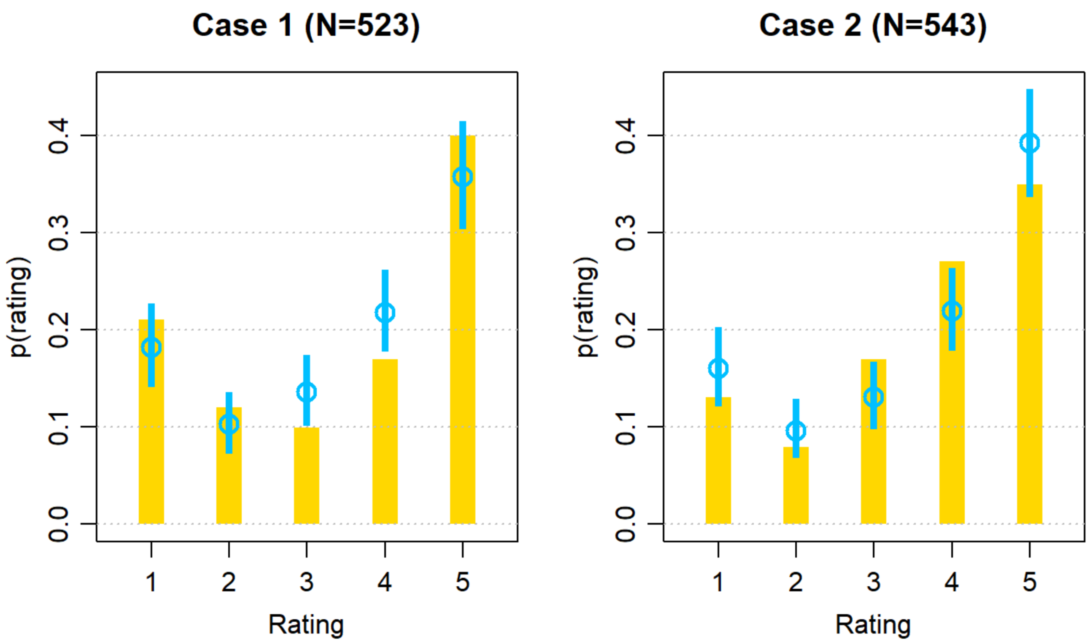

We see above that the fit of homogeneous-variance model is not very good, at least not as good as the heterogeneous-variance models.

8.3.5 Posterior details (Diffm Homv model, prior informed by subset)

Despite the poor fit of the model, the parameter estimates are displayed here for completeness:

Difference of means:

Code

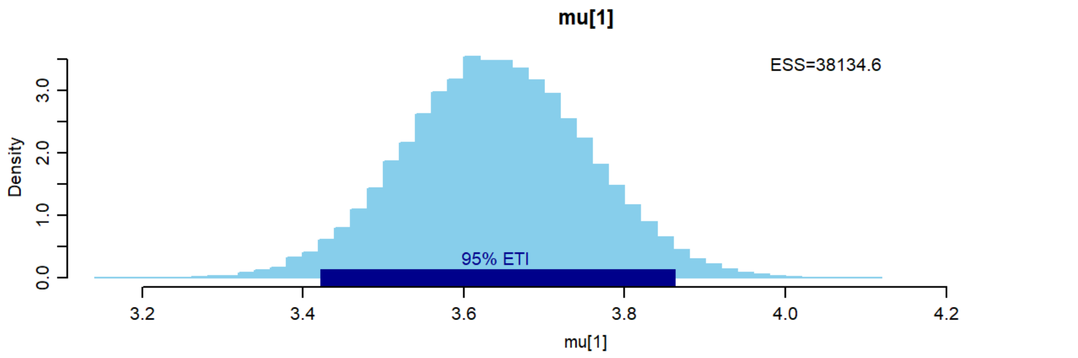

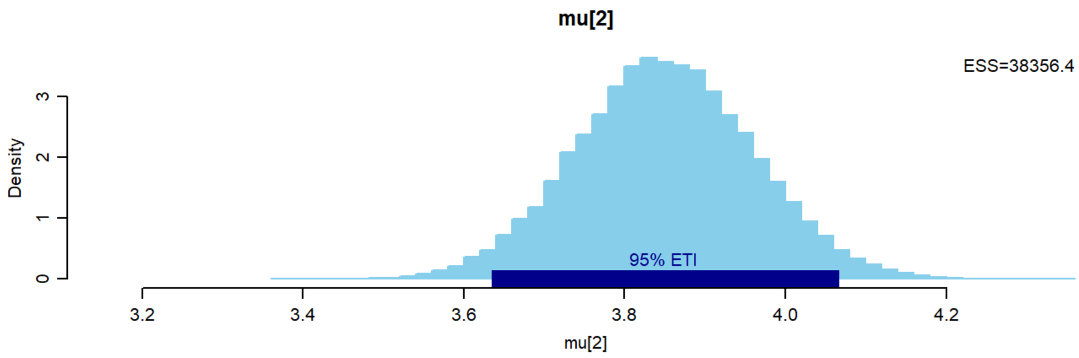

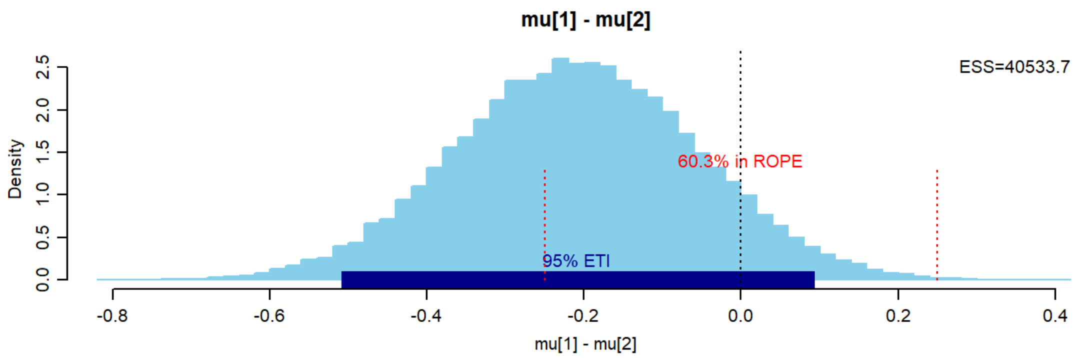

Effect size (Cohen's d), $(\mu_1 - \mu_2)\Big/\sqrt{(\sigma_1^2 + \sigma_2^2)/2}$:

Code

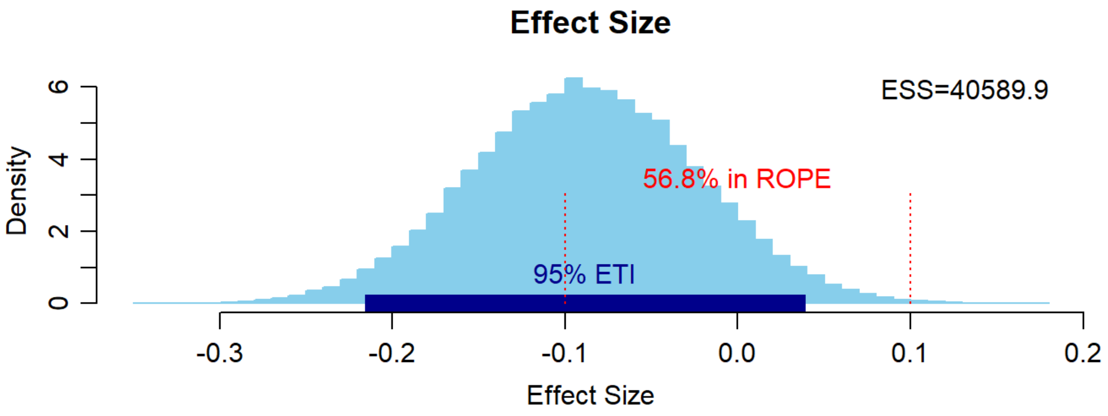

Code

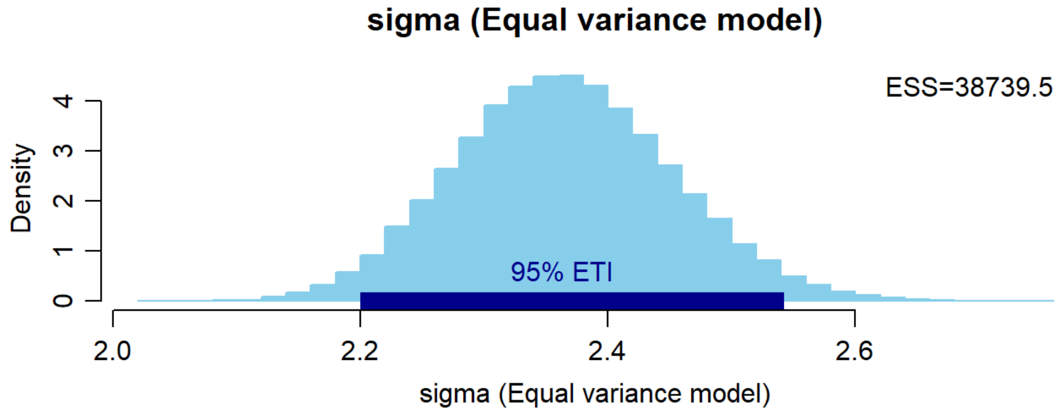

Again, the estimated parameter values above should not be taken too seriously, given the relatively poor fit of the model.

8.4 Sensitivity of Diffm, Homv model to prior

Below are graphical comparisons of the marginal posteriors from the three priors. The plot of posterior density relies on smoothing by a kernel with width determined heuristically. The posterior density is a natural way to view the mode and HDI. The plot of cumulative probability does not rely on smoothing (but is effectively smoothed by the pixel resolution). The cumulative probability is a natural way to view the median and ETI.

Code

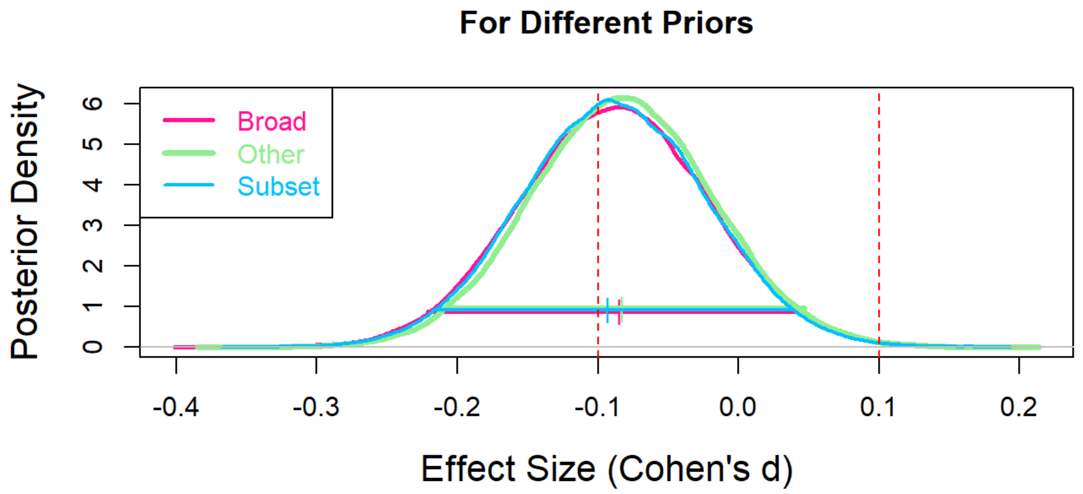

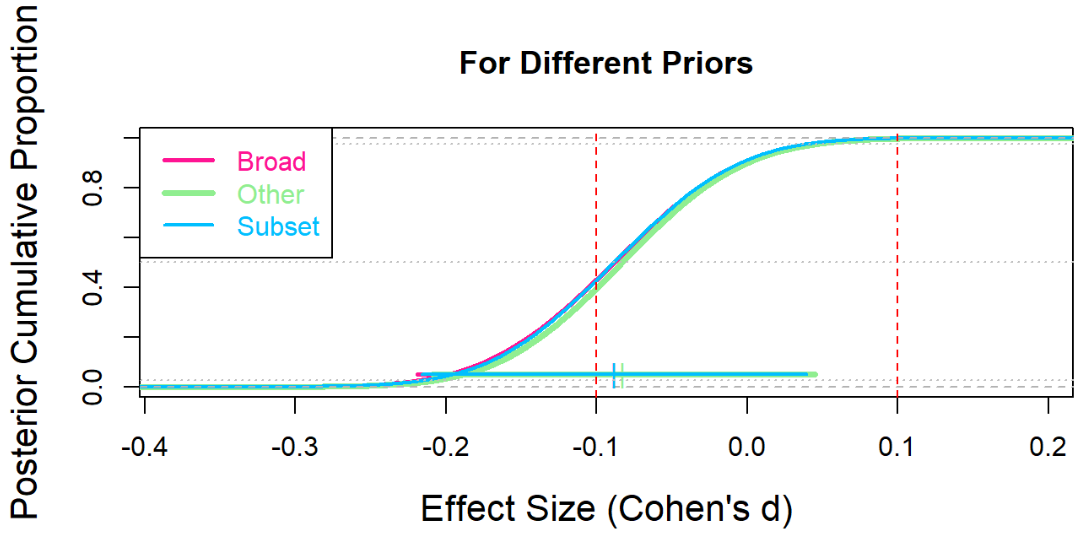

Above: Effect Size estimates from different priors. Broad = broad prior, Other = informed by other movies, Subset = informed by subset.

Code

Code

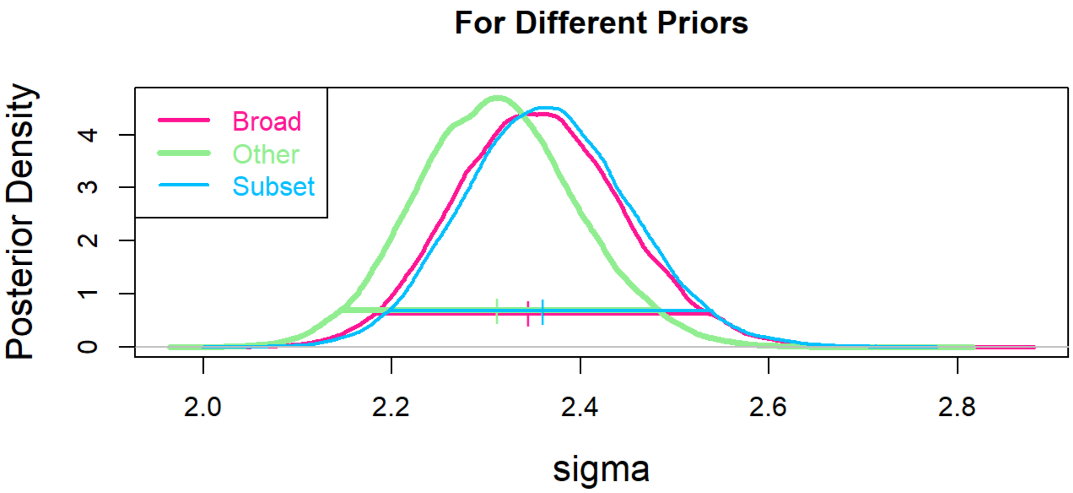

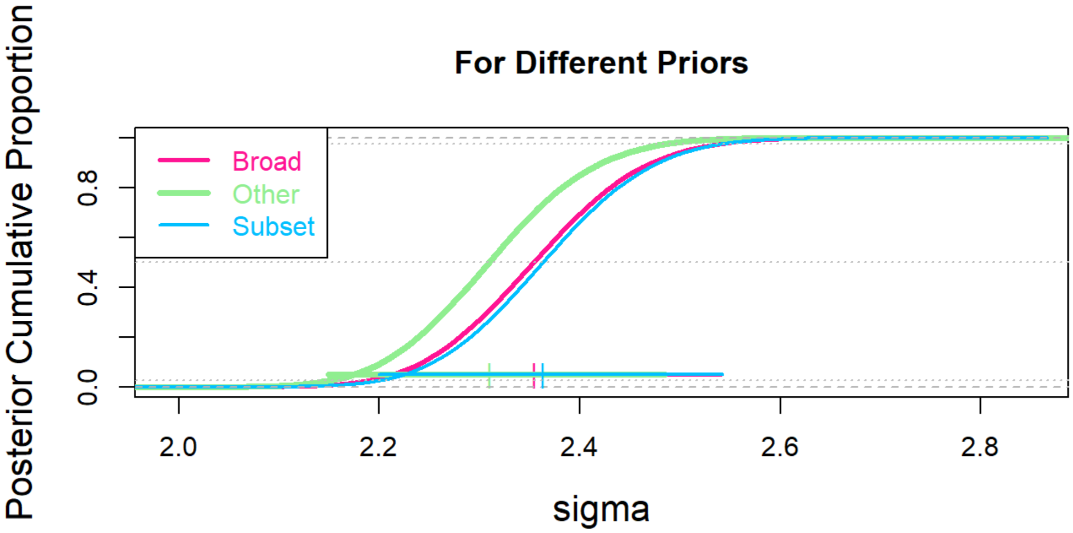

Above: Sigma estimates from different priors. Broad = broad prior, Other = informed by other movies, Subset = informed by subset.

Code

The impact of the different priors is made clear by the plot, but the magnitude of difference relative to the credible intervals and relative to the ROPE is very small.

9 Model Comparison for Hypothesis Testing

(Cf. BARG Step 4.D.)

The Bayes factor (BF) is the coefficient that converts the prior odds of the models to the posterior odds of the models, and the magnitude of the BF indicates the degree to which model probabilities are reallocated from their prior probabilities. Formally, for models $M1$ and $M2$, and for data D, the Bayes factor for $M1$ relative to $M2$ is

$$BF_{M1/M2} = \frac{p(M1|D)}{p(M2|D)} \Big/ \frac{p(M1)}{p(M2)}$$

where $p(M)$ is the prior probability of model M and $p(M|D)$ is its posterior probability. That relationship can be algebraically converted to a simple formula for computing the posterior probability of model $M1$ from its prior probability (assuming $p(M2) = 1 - p(M1)$):

$$p(M1|D) = p(M1)\, BF_{M1/M2} \Big/ \left(1 + p(M1)(BF_{M1/M2} - 1)\right)$$

This relationship between $p(M1|D)$ and $p(M1)$ is displayed in some graphs below, for the particular values of the BF's that result from these data and models. Finally, the relationship can be re-arranged to yield the prior model probability that achieves the critical posterior model probability (again assuming $p(M2) = 1 - p(M1)$):

$$p(M1)_{crit} = \frac{p(M1|D)_{crit}}{1 - p(M1|D)_{crit}} \Big/ \left(BF_{M1/M2} + \frac{p(M1|D)_{crit}}{1 - p(M1|D)_{crit}}\right)$$

This prior model probability is also annotated in the specific graphs below.

Bayes factors are computed here by using bridge sampling.[17,18] Bridge sampling is a general-purpose method for computing marginal likelihoods, hence Bayes factors, for arbitrary models and priors. Bridge sampling is quite flexible but must be used carefully (e.g., it can have issues with extremely broad prior distributions); see the Discussion section of Ref 18. The application here is well behaved.

To use the bridge sampling package in R, I must define functions that compute the unnormalized log posterior probability for each model. The functions for the three models are presented below.

Function to compute the unnormalized log posterior probability of Diffm Hetv model:

Code

Function to compute the unnormalized log posterior probability of Eqm Hetv model:

Code

Function to compute the unnormalized log posterior probability of Diffm Homv model:

Code

The bridge sampling package in R[18] also requires lower and upper bounds to be specified for all of the parameters in the model.

Diffm Hetv:

Code

Eqm Hetv:

Code

Diffm Homv:

Code

9.1 Broad prior

Before the `bridge_sampler()` function is called, the pseudo-random number generator is explicitly seeded for reproducible output (cf. BARG Step 6.H.). This also applies to all subsequent calls.

Diffm Hetv model: Compute marginal likelihood with bridge sampler.

Code

```
##
## Bridge sampling log marginal likelihood estimate
## (method = "normal", repetitions = 1):
##
##  -1606.216
##
## Error Measures:
##
##  Relative Mean-Squared Error: 1.953806e-07
##  Coefficient of Variation: 0.0004420188
##  Percentage Error: 0%
##
## Note:
## All error measures are approximate.
```

As indicated above, the percentage error from the bridge-sampling estimate is very small (only 0%), which suggests that the estimate of the marginal likelihood is very stable.

Eqm Hetv model: Compute marginal likelihood with bridge sampler.

Code

```
##
## Bridge sampling log marginal likelihood estimate
## (method = "normal", repetitions = 1):
##
##  -1603.692
##
## Error Measures:
##
##  Relative Mean-Squared Error: 9.535108e-08
##  Coefficient of Variation: 0.0003087897
##  Percentage Error: 0%
##
## Note:
## All error measures are approximate.
```

As indicated above, the percentage error from the bridge-sampling estimate is very small (only 0%), which suggests that the estimate of the marginal likelihood is very stable.

Diffm Homv model: Compute marginal likelihood with bridge sampler.

Code

```
##
## Bridge sampling log marginal likelihood estimate
## (method = "normal", repetitions = 1):
##
##  -1616.914
##
## Error Measures:
##
##  Relative Mean-Squared Error: 1.12639e-07
##  Coefficient of Variation: 0.0003356174
##  Percentage Error: 0%
##
## Note:
## All error measures are approximate.
```

As indicated above, the percentage error from the bridge-sampling estimate is very small (only 0%), which suggests that the estimate of the marginal likelihood is very stable.

Bayes factors and posterior model probabilities:

Compute and display the Bayes factor for a test of equal means:

Code

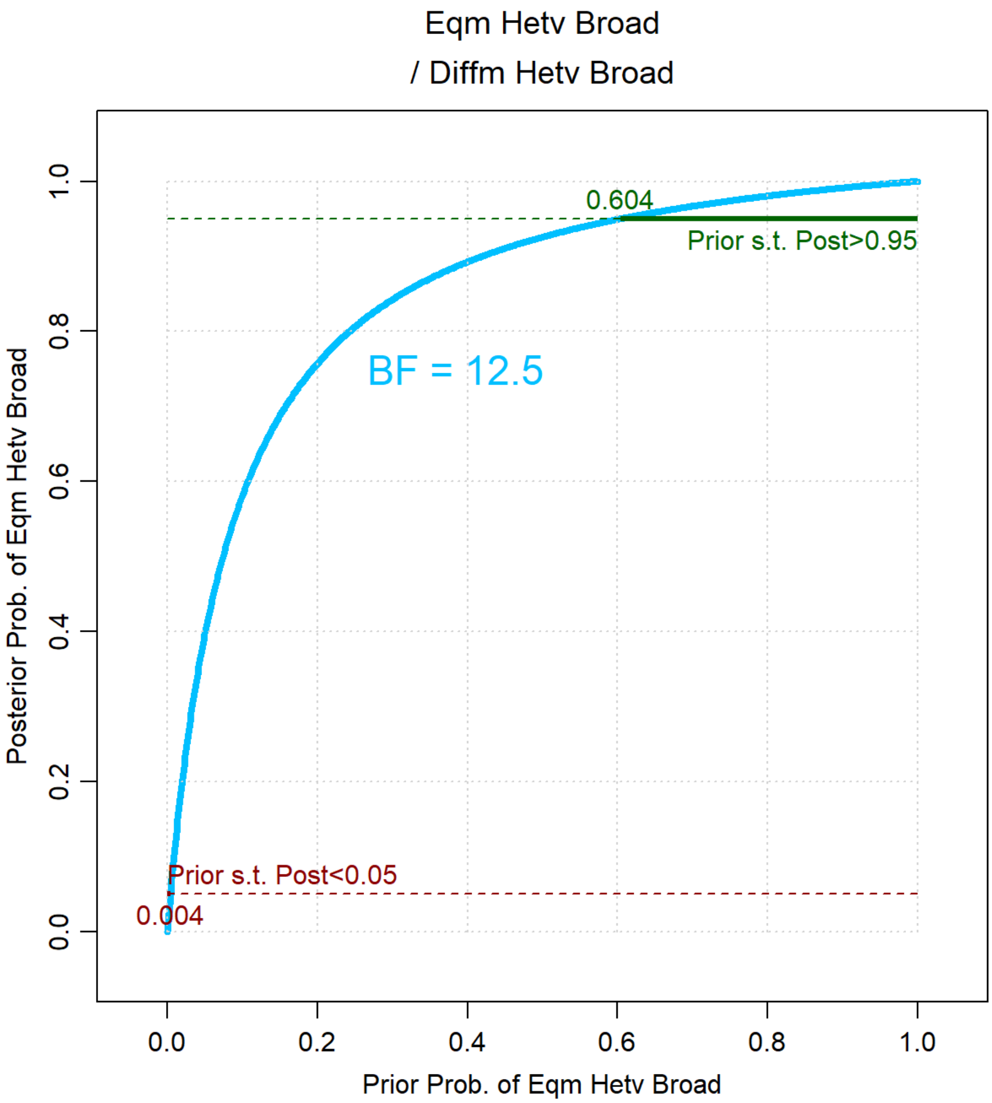

The plot above shows posterior model probabilities as a function of prior model probabilities. (Cf. BARG Step 3.C and Step 4.D.) The Bayes factor is 12.5 for Eqm Hetv Broad relative to Diffm Hetv Broad. To ‘accept’ Eqm Hetv Broad (relative to Diffm Hetv Broad) with a posterior probability of at least 0.95, Eqm Hetv Broad’s prior probability must be at least 0.604. To ‘reject’ Eqm Hetv Broad (relative to Diffm Hetv Broad) with a posterior probability less than 0.05, Eqm Hetv Broad’s prior probability must be less than 0.004.

Compute and display the Bayes factor for a test of equal variances:

Code

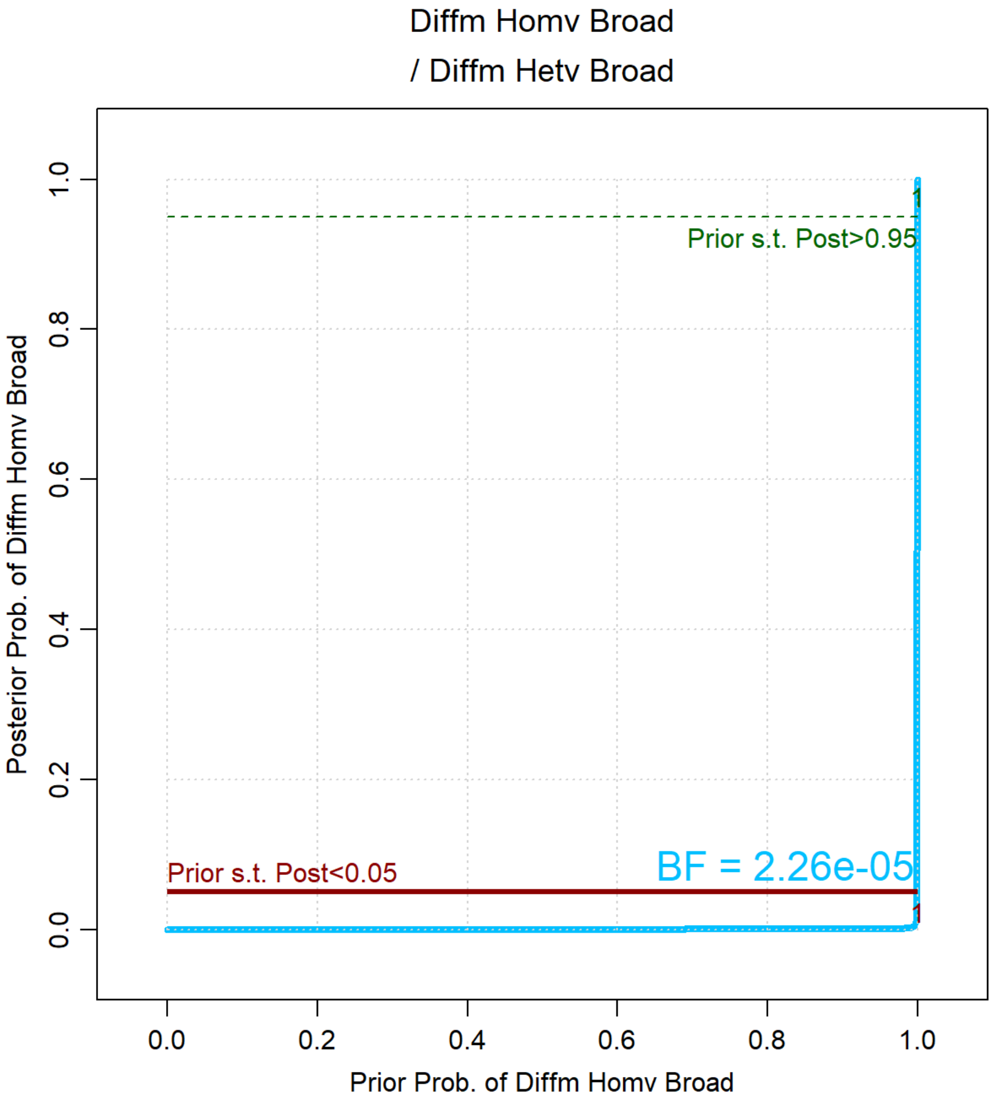

The plot above shows posterior model probabilities as a function of prior model probabilities. (Cf. BARG Step 3.C and Step 4.D.) The Bayes factor is 2.26e-05 for Diffm Homv Broad relative to Diffm Hetv Broad. To ‘accept’ Diffm Homv Broad (relative to Diffm Hetv Broad) with a posterior probability of at least 0.95, Diffm Homv Broad’s prior probability must be at least 1. To ‘reject’ Diffm Homv Broad (relative to Diffm Hetv Broad) with a posterior probability less than 0.05, Diffm Homv Broad’s prior probability must be less than 1.

9.2 Prior informed by other cases

Diffm Hetv model: Compute marginal likelihood with bridge sampler.

Code

```
##
## Bridge sampling log marginal likelihood estimate
## (method = "normal", repetitions = 1):
##
##  -1611.471
##
## Error Measures:
##
##  Relative Mean-Squared Error: 1.829462e-07
##  Coefficient of Variation: 0.0004277221
##  Percentage Error: 0%
##
## Note:
## All error measures are approximate.
```

As indicated above, the percentage error from the bridge-sampling estimate is very small (only 0%), which suggests that the estimate of the marginal likelihood is very stable.

Eqm Hetv model: Compute marginal likelihood with bridge sampler.

Code

```
##
## Bridge sampling log marginal likelihood estimate
## (method = "normal", repetitions = 1):
##
##  -1610.353
##
## Error Measures:
##
##  Relative Mean-Squared Error: 8.893592e-08
##  Coefficient of Variation: 0.0002982213
##  Percentage Error: 0%
##
## Note:
## All error measures are approximate.
```

As indicated above, the percentage error from the bridge-sampling estimate is very small (only 0%), which suggests that the estimate of the marginal likelihood is very stable.

Diffm Homv model: Compute marginal likelihood with bridge sampler.

Code

```
##
## Bridge sampling log marginal likelihood estimate
## (method = "normal", repetitions = 1):
##
##  -1622.718
##
## Error Measures:
##
##  Relative Mean-Squared Error: 9.712535e-08
##  Coefficient of Variation: 0.0003116494
##  Percentage Error: 0%
##
## Note:
## All error measures are approximate.
```

As indicated above, the percentage error from the bridge-sampling estimate is very small (only 0%), which suggests that the estimate of the marginal likelihood is very stable.

Bayes factors and posterior model probabilities:

(Cf. BARG Steps 1 and 4, regarding BF and model probabilities.)

Compute and display the Bayes factor for a test of equal means:

Code

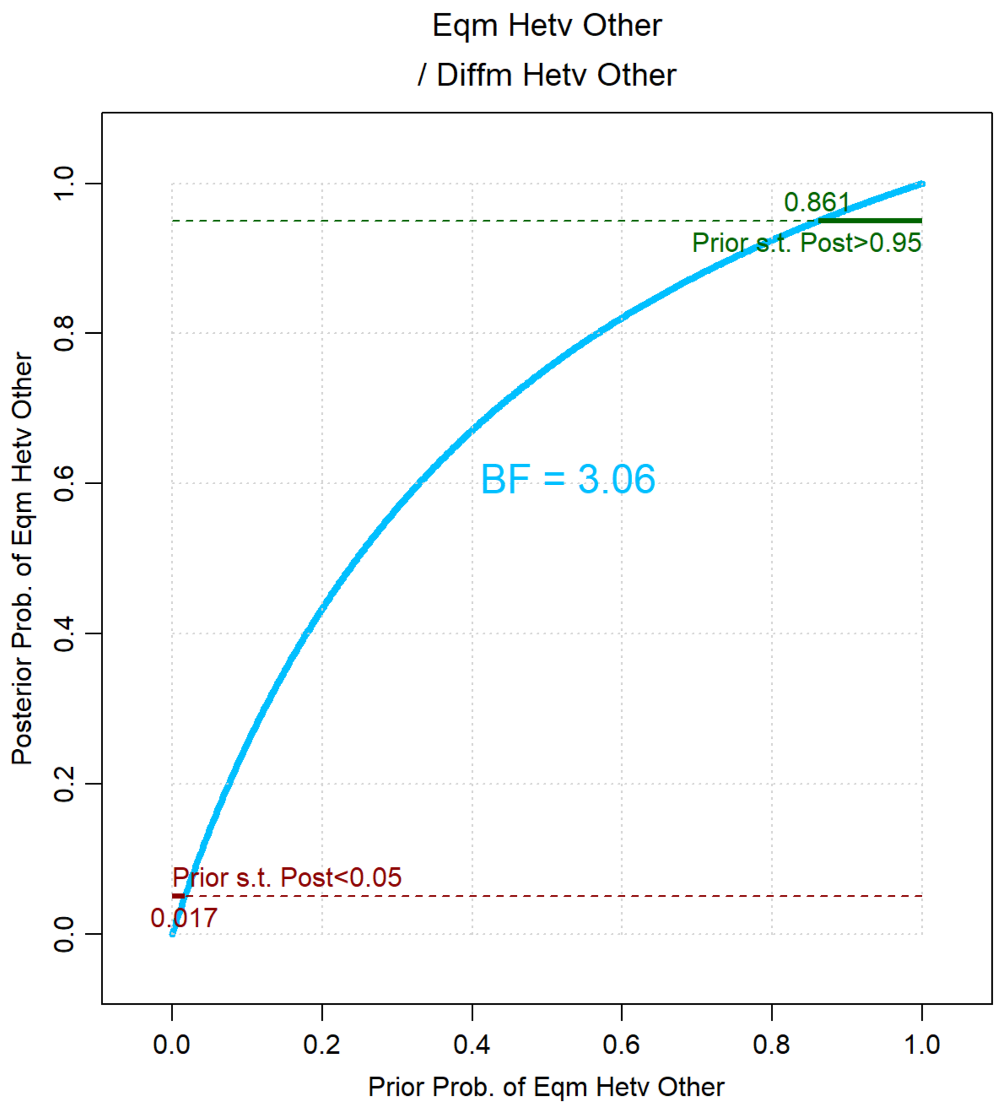

The plot above shows posterior model probabilities as a function of prior model probabilities. (Cf. BARG Step 3.C and Step 4.D.) The Bayes factor is 3.06 for Eqm Hetv Other relative to Diffm Hetv Other. To ‘accept’ Eqm Hetv Other (relative to Diffm Hetv Other) with a posterior probability of at least 0.95, Eqm Hetv Other’s prior probability must be at least 0.861. To ‘reject’ Eqm Hetv Other (relative to Diffm Hetv Other) with a posterior probability less than 0.05, Eqm Hetv Other’s prior probability must be less than 0.017.

Compute and display the Bayes factor for a test of equal variances:

Code

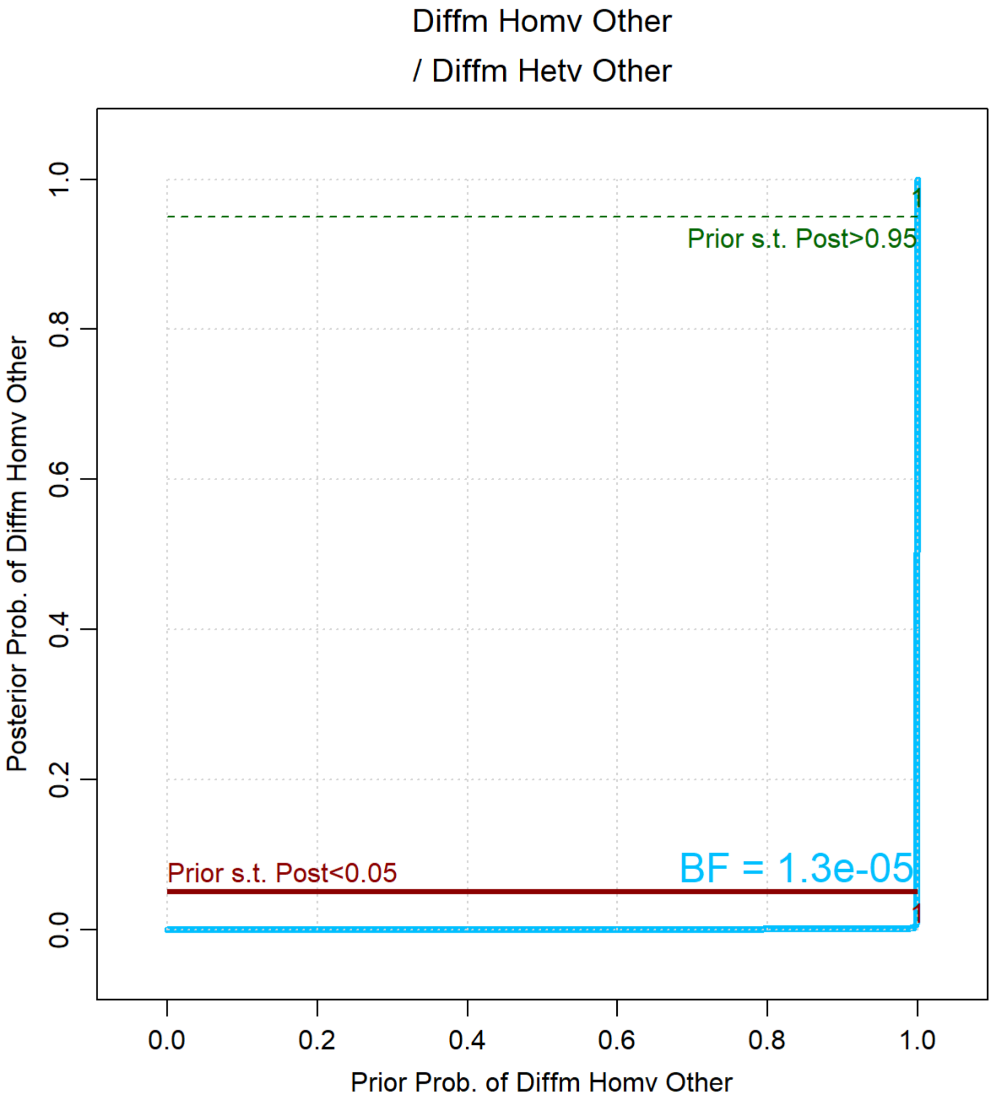

The plot above shows posterior model probabilities as a function of prior model probabilities. (Cf. BARG Step 3.C and Step 4.D.) The Bayes factor is 1.3e-05 for Diffm Homv Other relative to Diffm Hetv Other. To ‘accept’ Diffm Homv Other (relative to Diffm Hetv Other) with a posterior probability of at least 0.95, Diffm Homv Other’s prior probability must be at least 1. To ‘reject’ Diffm Homv Other (relative to Diffm Hetv Other) with a posterior probability less than 0.05, Diffm Homv Other’s prior probability must be less than 1.

9.3 Prior informed by subset

Diffm Hetv model: Compute marginal likelihood with bridge sampler.

Code

```
##
## Bridge sampling log marginal likelihood estimate
## (method = "normal", repetitions = 1):
##
##  -1596.646
##
## Error Measures:
##
##  Relative Mean-Squared Error: 1.854802e-07
##  Coefficient of Variation: 0.0004306742
##  Percentage Error: 0%
##
## Note:
## All error measures are approximate.
```

As indicated above, the percentage error from the bridge-sampling estimate is very small (only 0%), which suggests that the estimate of the marginal likelihood is very stable.

Eqm Hetv model: Compute marginal likelihood with bridge sampler.

Code

```
##
## Bridge sampling log marginal likelihood estimate
## (method = "normal", repetitions = 1):
##
##  -1595.058
##
## Error Measures:
##
##  Relative Mean-Squared Error: 8.067185e-08
##  Coefficient of Variation: 0.0002840279
##  Percentage Error: 0%
##
## Note:
## All error measures are approximate.
```

As indicated above, the percentage error from the bridge-sampling estimate is very small (only 0%), which suggests that the estimate of the marginal likelihood is very stable.

Diffm Homv model: Compute marginal likelihood with bridge sampler.

Code

```
##
## Bridge sampling log marginal likelihood estimate
## (method = "normal", repetitions = 1):
##
##  -1608.237
##
## Error Measures:
##
##  Relative Mean-Squared Error: 9.385726e-08
##  Coefficient of Variation: 0.0003063613
##  Percentage Error: 0%
##
## Note:
## All error measures are approximate.
```

As indicated above, the percentage error from the bridge-sampling estimate is very small (only 0%), which suggests that the estimate of the marginal likelihood is very stable.

Bayes factors and posterior model probabilities:

(Cf. BARG Steps 1 and 4, regarding BF and model probabilities.)

Compute and display the Bayes factor for a test of equal means:

Code

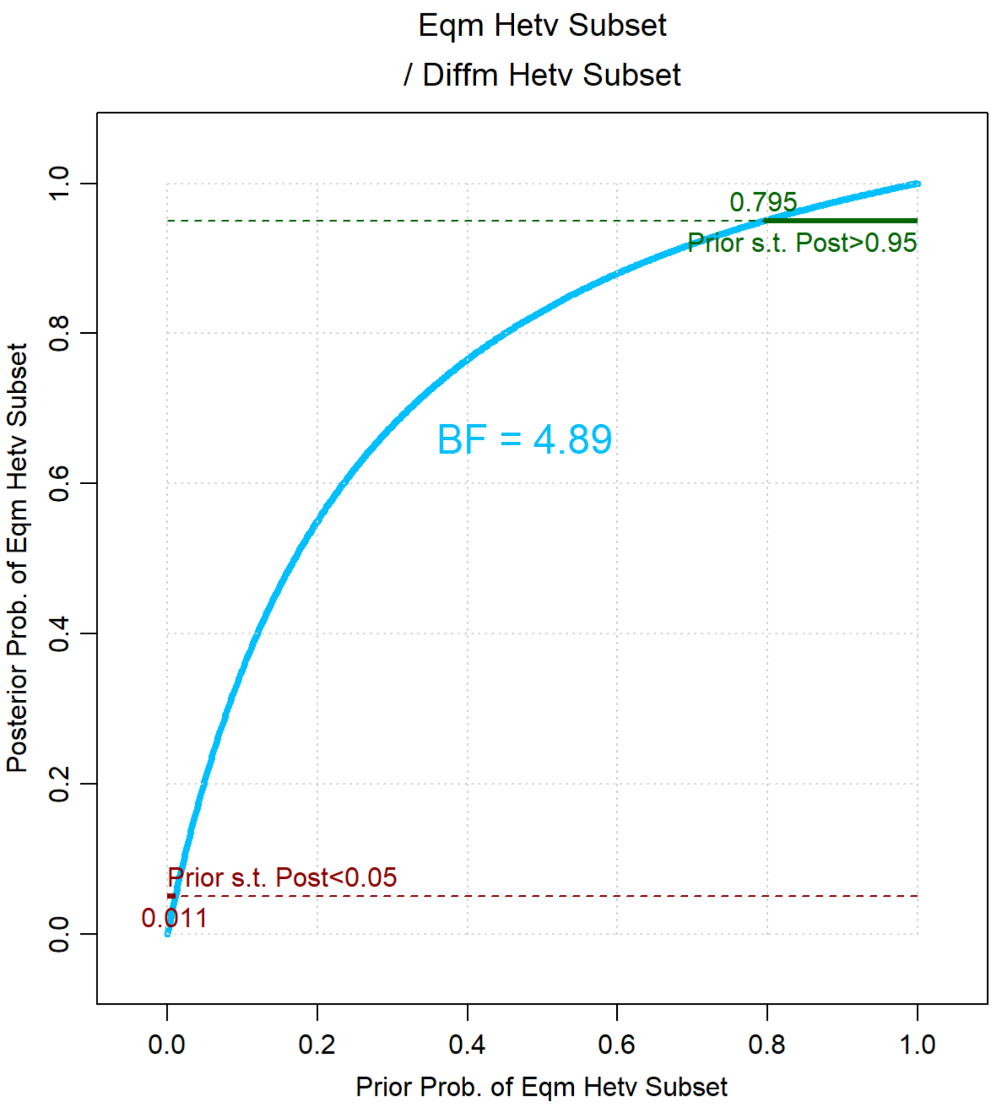

The plot above shows posterior model probabilities as a function of prior model probabilities. (Cf. BARG Step 3.C and Step 4.D.) The Bayes factor is 4.89 for Eqm Hetv Subset relative to Diffm Hetv Subset. To ‘accept’ Eqm Hetv Subset (relative to Diffm Hetv Subset) with a posterior probability of at least 0.95, Eqm Hetv Subset’s prior probability must be at least 0.795. To ‘reject’ Eqm Hetv Subset (relative to Diffm Hetv Subset) with a posterior probability less than 0.05, Eqm Hetv Subset’s prior probability must be less than 0.011.

Compute and display the Bayes factor for a test of equal variances:

Code

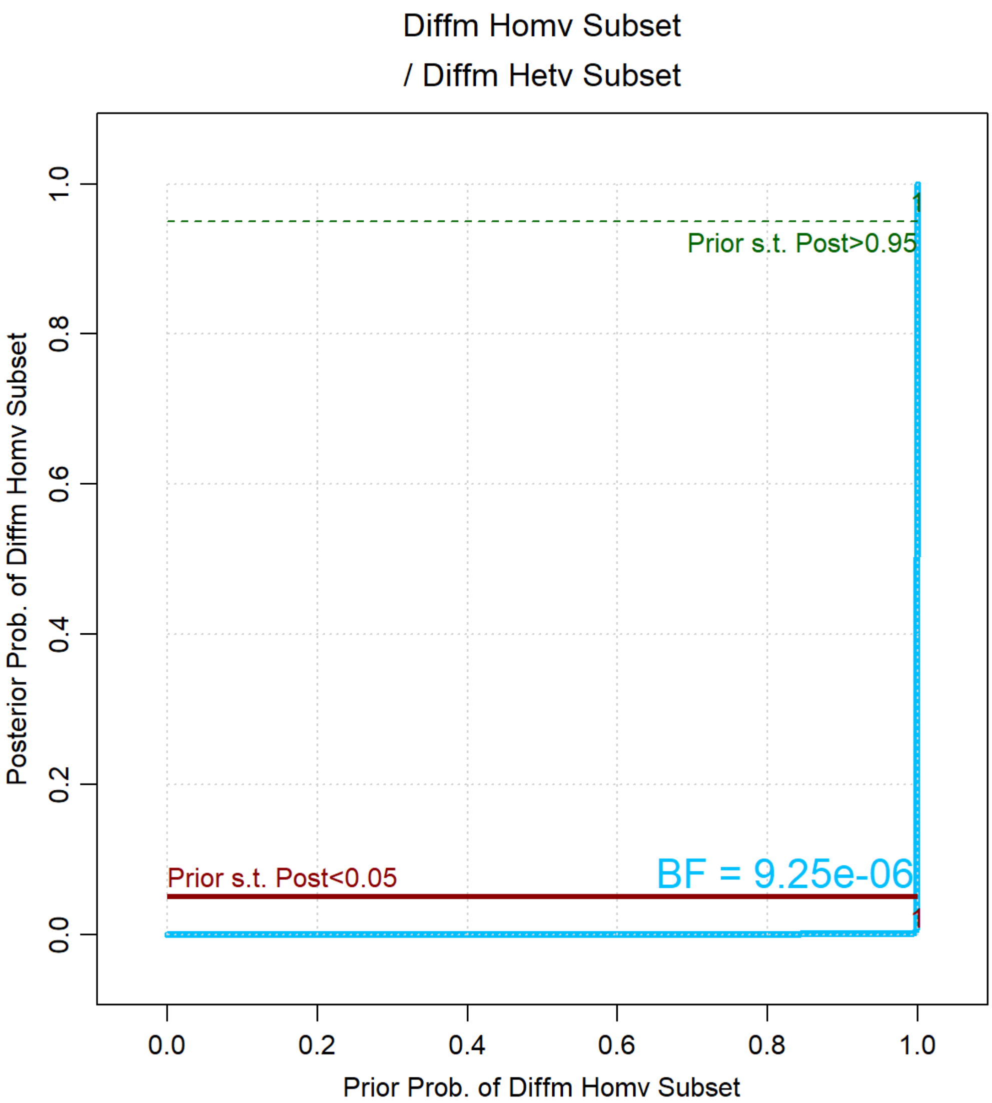

The plot above shows posterior model probabilities as a function of prior model probabilities. (Cf. BARG Step 3.C and Step 4.D.) The Bayes factor is 9.25e-06 for Diffm Homv Subset relative to Diffm Hetv Subset. To ‘accept’ Diffm Homv Subset (relative to Diffm Hetv Subset) with a posterior probability of at least 0.95, Diffm Homv Subset’s prior probability must be at least 1. To ‘reject’ Diffm Homv Subset (relative to Diffm Hetv Subset) with a posterior probability less than 0.05, Diffm Homv Subset’s prior probability must be less than 1.

9.4 Summary of BF’s under different priors

(Cf. BARG Step 5.D. and 5.E.)

9.4.1 Equal Means vs Different Means

Code

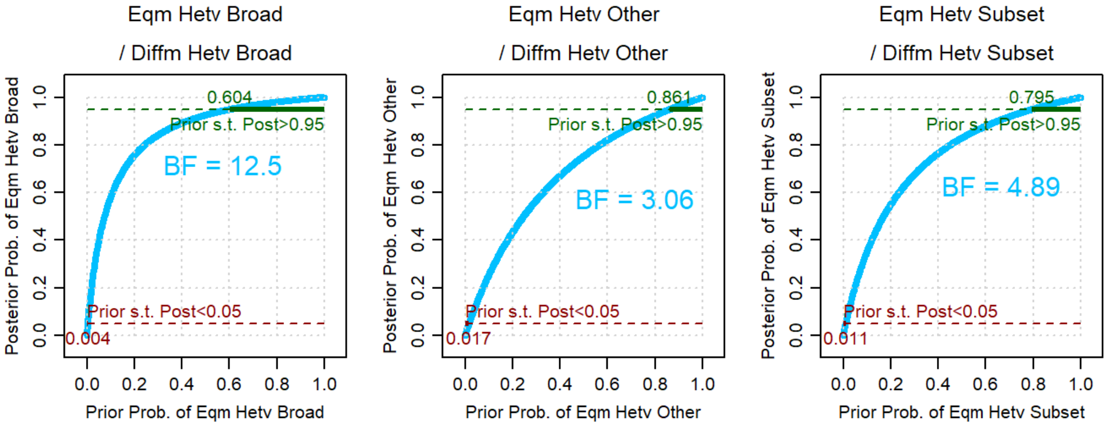

Code

Code

```
##            BF CritPriorProb
## Broad   12.50         0.604
## Other    3.06         0.861
## Subset   4.89         0.795
```

The table above has a different row for each prior, corresponding to the preceding graphs. The column labelled “CritPriorProb” is the *minimum* prior probability of the equal-mean model required for its posterior probability to *exceed* 0.95, that is, to “accept” it. *To “accept” the equal-mean (Eqm) model, its prior probability would have to be at least 0.604 even for the most extreme BF, which is not very plausible because the underlying means of two different movies are very unlikely to be exactly equal, that is, the prior probability of the Eqm model is very small*.

The results indicate that there is some change in the BF across priors, though all lean toward the equal-mean model. Interestingly, although the Broad and Subset priors yielded posterior estimates that were virtually identical to each other in the full model, they yield somewhat different BF’s. Presumably this arises, at least in part, because a broad prior distribution on the target parameter in the non-null model “dilutes” the prior probability of non-null values across a wide range, such that non-null parameter values that happen fit the data have small prior probability, resulting in weakened support for the non-null model.

Decisions made by the posterior model probability (via the BF) agree with decisions made by considering parameter magnitudes and ROPEs. That is, the parameter estimates suggested little difference between means, but the distributions had a non-trivial amount of the posterior fall outside the ROPE, hence a decision was withheld. This agrees with the BF’s, which lean toward the equal-mean model, but are not extreme enough to decide to accept the equal-mean model.

9.4.2 Homogeneous Variances vs Heterogeneous Variances

Code

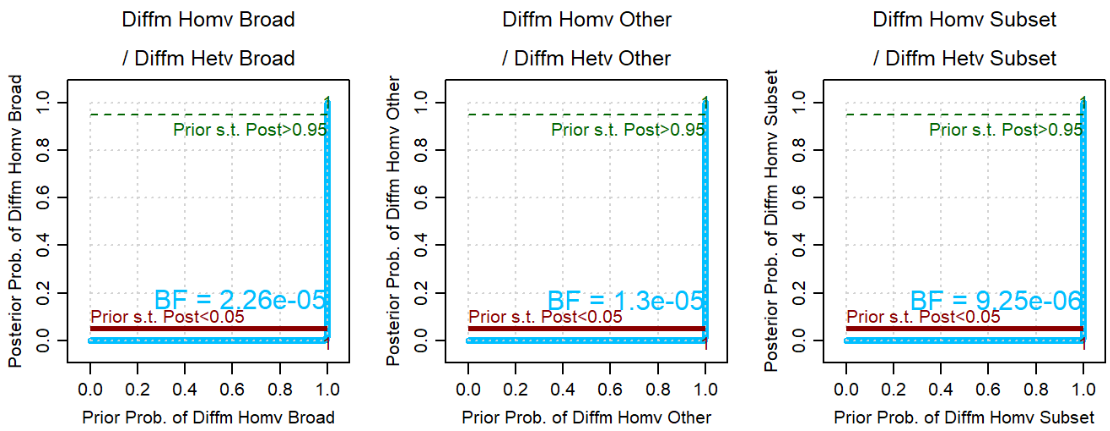

Code

Code

```
##                  BF CritPriorProb
## Broad  2.258193e-05     0.9995711
## Other  1.304307e-05     0.9997522
## Subset 9.251810e-06     0.9998242
```

The table above has a different row for each prior, corresponding to the preceding graphs. The column labelled “CritPriorProb” is the *maximum* prior probability of the homogeneous-variance model required for its posterior probability to be *less* than 0.05, that is, to “reject” it. In all cases, virtually any prior probability on the homogeneous-variance model (other than 1.0) results in the homogeneous-variance model (Homv) being rejected.

The results indicate that there is some change in the BF across priors, though all lean strongly away from homogeneous-variance model. Interestingly, although the Broad and Subset priors yielded posterior estimates that were virtually identical to each other in the full model, they yield somewhat different BF’s. Presumably this arises, at least in part, because a broad prior distribution on the target parameter in the non-null model “dilutes” the prior probability of non-null values across a wide range, such that non-null parameter values that happen fit the data have small prior probability, resulting in weakened support for the non-null model.

Decisions made by the posterior model probability (via the BF) agree with decisions made by considering parameter magnitudes and ROPEs. That is, the parameter estimates indicated large differences between variances with posterior distributions that fell well outside the ROPE, and the BF’s indicated a huge range of prior probabilities that would lead to posterior probabilities that reject the homogeneous-variance model.

10 How to reproduce this document

(Cf. BARG Step 6.)

10.1 Install Software

Install the following software. It is all free. Even if you already have the software, *it is a good idea to install the most recent versions*.

- The computer-programming language **R**.[19] Download from https://www.r-project.org/ (https://www.r-project.org/)
- The editing environment **RStudio**. Download from https://rstudio.com/products/rstudio/download/#download (https://rstudio.com/products/rstudio/download/#download)
- The Bayesian model-specification software and MCMC sampler **JAGS**.[20] Download from http://mcmc-jags.sourceforge.net/ (http://mcmc-jags.sourceforge.net/)

After downloading, be sure to install.

10.2 Retrieve Files

All the files needed to reproduce this document are at https://osf.io/w7cph/ (https://osf.io/w7cph/). They include the following:

- `BARG-Supplement.Rmd` The R Markdown source file for this document.
- `MoviesData.csv` The data file that specifies the ratings of the movies.
- `BARG-references.bib` An auxiliary file with reference information.
- `apa.csl` or `nature.csl` An auxiliary file for formatting the references.
- `LiddellKruschke2018Fig1.png` An auxiliary image file imported into the summary document.
- `BayesModelProb_FuncDef.R` An auxiliary file that defines additional functions.

Save all of those files *together in the same folder* on your computer.

Optionally, you can also download the *folder*, `BARG-Supplement-Output` , which contains numerical output from previously running `BARG-Supplement.Rmd` . The files in this folder are only useful for saving time when typesetting `BARG-Supplement.Rmd` without recomputing the MCMC chains. If you do download the folder, place it (with its contents) as a folder within the folder you used for the files listed above.

10.3 Run the Analysis

- Open `BARG-Supplement.Rmd` in RStudio.
- When opening the file, RStudio may alert you that various packages are needed, and ask if you want to install them. Yes, do install them. These packages include knitr,[21] rjags,[22] coda,[12] runjags,[23] bridgesampling,[18] DT,[24] emdbook,[25] psych,[26] and tictoc,[27] among others.
- If using previously installed packages, *it is a good idea to update all packages* (in RStudio, use menu: Tools $\rightarrow$ Check for Package Updates).
- *Be sure that the folder with the files is R's current working directory*. In RStudio's pull-down menu, use Session $\rightarrow$ Set Working Directory $\rightarrow$ Choose Directory.
(Do not use Session $\rightarrow$ Set Working Directory $\rightarrow$ To Source File Location, because it sometimes does not work.)
- The R Markdown file is set to re-run the MCMC chains when it is processed, which will take several minutes (see next bullet). Whether to compute new MCMC chains or to use previously stored MCMC results is set by a line early in the R Markdown source file. In the RStudio editing pane, search the file `BARG-Supplement.Rmd` for `runNewMCMC = TRUE` . If you want to use previously saved MCMC chains, then edit that line to `runNewMCMC = FALSE` and be sure that the folder `BARG-Supplement-Output` and all of its contents are in your current working directory. (Cf. BARG Step 6.G.)
- Click the Knit button (Knit to HTML) at the top of the editing pane in RStudio. The result will be an HTML file. **Processing this document with its MCMC analyses took a little more than 7.93 minutes on a modest desktop computer.**

10.4 R Session Info

The exact versions of the software that were used to produce this analysis and document are reported below:

Code

```
## [1] "Mon Jul 05 15:20:24 2021"
```

Code

```
## R version 4.0.3 (2020-10-10)
## Platform: x86_64-w64-mingw32/x64 (64-bit)
## Running under: Windows 10 x64 (build 18362)
##
## Matrix products: default
##
## locale:
## [1] LC_COLLATE=English_United States.1252
## [2] LC_CTYPE=English_United States.1252
## [3] LC_MONETARY=English_United States.1252
## [4] LC_NUMERIC=C
## [5] LC_TIME=English_United States.1252
##
## attached base packages:
## [1] stats     graphics  grDevices utils     datasets  methods   base
##
## other attached packages:
## [1] tictoc_1.0           psych_2.0.12         DT_0.17
## [4] emdbook_1.3.12       bridgesampling_1.0-0 runjags_2.0.4-6
## [7] rjags_4-10           coda_0.19-4
##
## loaded via a namespace (and not attached):
##  [1] Rcpp_1.0.6          compiler_4.0.3      plyr_1.8.6
##  [4] highr_0.8           tools_4.0.3         digest_0.6.27
##  [7] lifecycle_0.2.0     jsonlite_1.7.2      evaluate_0.14
## [10] nlme_3.1-149        lattice_0.20-41     png_0.1-7
## [13] rlang_0.4.9         Matrix_1.2-18       crosstalk_1.1.1
## [16] yaml_2.2.1          parallel_4.0.3      mvtnorm_1.1-1
## [19] xfun_0.19           stringr_1.4.0       knitr_1.30
## [22] htmlwidgets_1.5.3   stats4_4.0.3        grid_4.0.3
## [25] R6_2.5.0            bdsmatrix_1.3-4     rmarkdown_2.6
## [28] magrittr_2.0.1      scales_1.1.1        htmltools_0.5.0
## [31] MASS_7.3-53         mnormt_2.0.2        colorspace_2.0-0
## [34] bbmle_1.0.23.1      numDeriv_2016.8-1.1 Brobdingnag_1.2-6
## [37] KernSmooth_2.23-17  stringi_1.5.3       munsell_0.5.0
## [40] tmvnsim_1.0-2
```

This concludes the example of analysis and reporting.

---

11 Review of previous Bayesian reporting guidelines

This section reviews many previous guidelines for reporting Bayesian analyses, in approximately chronological order. (This section is independent of the previous sections regarding the example of a Bayesian analysis and its reportage.)

The BayesWatch list by the authors of Ref 28 (Ch. 8) occurred in the context of a 136 page monograph about Bayesian methods. The BARG covers everything in the BayesWatch list (except for one item idiosyncratic to technology assessment). Unlike most subsequent lists, the BayesWatch list explicitly included reporting the loss function used for decision making. But most studies, including the examples Ref 28 used to illustrate BayesWatch, have no specific loss function. Ref 28 did, however, provide examples

of ROPEs, which were referred to as a “clinically important difference.” Unfortunately their examples did not report some of the essential details in the BARG, such as the ESS of MCMC chains. A clear example of a sensitivity analysis, using three different priors, was shown in Ch. 9 (pp. 59-62).

The BaSiS (Bayesian Standards in Science) list reported in Ref 29 is a brief and terse document in draft form without much detail, but which nevertheless has been a landmark for subsequent lists. In its few details about reporting the posterior distribution, the BaSiS list included some optional details not in the BARG, namely, the “shape of the posterior densities of individual parameters” and “joint posterior probability intervals.” The BARG does not explicitly mention these because typically the shape of the posterior distribution is unimodal and skewness will be apparent by the asymmetry of the CI around the central tendency, and it is not clear from BaSiS exactly when joint intervals are needed. The BaSiS is brief and leaves out many items in the BARG.

The ROBUST (Reporting Of Bayes Used in clinical STudies) list was developed by the authors of Ref 30 by polling two dozen statisticians and reviewing the BayesWatch and BaSiS lists. The authors of Ref 30 selected the items mentioned by a majority of experts. Nevertheless, the resulting list is brief and subsumed by the BARG. ROBUST was intended as a minimal list for brief reports and leaves out many essential items such as details of the MCMC computations, separate considerations for hypothesis testing, etc. By contrast, the BARG recommends extensive reporting, but relegating some details to appendices or supplementary material if demanded by journal format or audience.

Guidelines for reporting Bayesian analyses were provided in Ch. 23 (pp. 619–622) of Ref 31 and revised in Ch. 25 (pp. 721–725) of Ref 3. The BARG is strongly influenced by those guidelines, and incorporates all of their points in an expanded structure.

The SAMPL (Statistical Analyses and Methods in the Published Literature) guidelines by Ref 32 gave checklists for reporting some basic types of analyses. It included a separate short checklist for reporting Bayesian analyses, with no explanation for any of the points. The BARG includes them all.

Another brief checklist of “minimum reporting guidelines” appeared with some explanation in the context of a tutorial article regarding Bayesian linear regression (Ref 33, Section 4.1, p. 74). Again, the BARG includes all the mentioned points.

A checklist called WAMBS (When to worry and how to Avoid the Misuse of Bayesian Statistics) was presented by Ref 34. The WAMBS points out that prior distributions on higher-level parameters might produce unanticipated biases on the posterior distribution, so care should be taken to check that priors intended to be uninfluential really are. When considering MCMC diagnostics, the WAMBS did not mention effective sample size (ESS), although ESS is described in a subsequent tutorial.[35] The WAMBS instead recommended a procedure of repeating the analysis with double the chain length, which might not be practical with time-consuming computations and might still not achieve sufficient ESS. The WAMBS did not discriminate between continuous parameter estimation and model/hypothesis comparison, and did not discuss Bayes factors. The WAMBS was updated in Ref 7 to a 10-point checklist which does highlight ESS and other important technical aspects. The BARG covers all the goals of the WAMBS, and includes various additional points such as treatment of posterior model probabilities in model comparison and hypothesis testing.

The 2018 APA reporting standards in Ref 36 included a small subsection regarding Bayesian analyses (Table 8, p. 20). The list is fairly extensive but has no explanatory text accompanying it nor any references to other sources, which is unfortunate because it includes some cryptic items such as: “Describe the unnormalized or normalized likelihood if the prior distribution is informative.” It does not mention effective sample size (ESS) of the MCMC chains. It has no sequential structure, and therefore would be difficult to use as a guide to conducting an analysis.

There is a reporting checklist in guidelines for using the JASP software,[37] at the end of its Table 1. The overall guidelines include many important aspects of planning, executing, interpreting, and reporting a Bayesian analysis. However, the guidelines do not mention the importance of checking and reporting MCMC details such as ESS. The Table 1 checklist indicates that if there is hypothesis testing then Bayes factors should be reported, but the checklist does not say that posterior probabilities should be reported. (The graphical JASP output presents numerical BF’s but presents posterior probabilities only as pie charts assuming fixed 50/50 prior probabilities.) The guidelines highlight categorical labels of Bayes factors in Fig. 4 (e.g., “Bayes factors greater than 10 are considered strong evidence” p. 821) despite also cautioning against using Bayes factors to make decisions (“These classifications are heuristic and should not be misused as an absolute rule for all-or-nothing conclusions”, p. 821). Except for a few aspects of research design that fall outside the scope of reporting results, the BARG encompasses the JASP reporting guidelines.

Ref 38 presented a “thinking guideline” for Bayesian analyses in Box 2 of their short article. It provides some important points for thinking clearly about goals and purposes of analysis, all of which are included in the BARG.

In conclusion, the BARG acknowledges and incorporates the points of previous guidelines, but with (relative to various predecessors) greater generality, thoroughness, explanation, and sequentiality. As mentioned in the main article, it is hoped that the BARG will be useful to researchers, authors, reviewers, editors, educators, and students.

12 References

1. Liddell, T. M. & Kruschke, J. K. Analyzing ordinal data with metric models: What could possibly go wrong? *Journal of Experimental Social Psychology* **79**, 328–348 (2018).

2. Farrell, S. & Lewandowsky, S. *Computational modeling of cognitiion and behavior*. (Cambridge University Press, 2018).

3. Kruschke, J. K. *Doing Bayesian data analysis, Second Edition: A tutorial with R, JAGS, and Stan*. (Academic Press, 2015).

4. Kruschke, J. K. & Liddell, T. M. Bayesian data analysis for newcomers. *Psychonomic Bulletin & Review* **25**, 155–177 (2018).

5. Kruschke, J. K. & Liddell, T. M. The Bayesian new statistics: Hypothesis testing, estimation, meta-analysis, and power analysis from a Bayesian perspective. *Psychonomic Bulletin & Review* **25**, 178–206 (2018).

6. McElreath, R. *Statistical rethinking: A Bayesian course with examples in R and Stan, 2nd edition*. (CRC Press, 2020).

7. van de Schoot, R. *et al.* Bayesian statistics and modelling. *Nature Reviews Methods Primers* **1**, 1–26 (2021).

8. Hu, N., Zhang, J. & Pavlou, P. A. Overcoming the J-shaped distribution of product reviews. *Communications of the ACM* **52**, 144–147 (2009).

9. Bürkner, P.-C. & Vuorre, M. Ordinal regression models in psychology: A tutorial. *Advances in Methods and Practices in Psychological Science* **2**, 77–101 (2019).

10. Carpenter, B. *et al.* Stan: A probabilistic programming language. *Journal of Statistical Software* **76**, (2017).

11. Brooks, S. P. & Gelman, A. General methods for monitoring convergence of iterative simulations. *Journal of Computational and Graphical Statistics* **7**, 434–455 (1998).

12. Plummer, M., Best, N., Cowles, K. & Vines, K. CODA: Convergence diagnosis and output analysis for MCMC. *R News* **6**, 7–11 (2006).

13. Kruschke, J. K. Rejecting or accepting parameter values in Bayesian estimation. *Advances in Methods and Practices in Psychological Science* **1**, 270–280 (2018).

14. Cohen, J. *Statistical power analysis for the behavioral sciences*. (Erlbaum, 1988).

15. Berger, J. O. & Pericchi, L. R. The intrinsic Bayes factor for model selection and prediction. *Journal of the American Statistical Association* **91**, 109–122 (1996).

16. Kary, A., Taylor, R. & Donkin, C. Using Bayes factors to test the predictions of models: A case study in visual working memory. *Journal of Mathematical Psychology* **72**, 210–219 (2016).

17. Gronau, Q. F. *et al.* A tutorial on bridge sampling. *Journal of Mathematical Psychology* **81**, 80–97 (2017).

18. Gronau, Q. F., Singmann, H. & Wagenmakers, E.-J. bridgesampling: An R package for estimating normalizing constants. *Journal of Statistical Software* **92**, 1–29 (2020).

19. R Core Team. *R: A language and environment for statistical computing*. (R Foundation for Statistical Computing, 2020).

20. Plummer, M. JAGS: A program for analysis of Bayesian graphical models using Gibbs sampling. in *Proceedings of the 3rd international workshop on distributed statistical computing (dsc 2003)* (2003).

21. Xie, Y. *knitr: A general-purpose package for dynamic report generation in R*. (2020).

22. Plummer, M. *rjags: Bayesian graphical models using MCMC*. (2019).

23. Denwood, M. J. runjags: An R package providing interface utilities, model templates, parallel computing methods and additional distributions for MCMC models in JAGS. *Journal of Statistical Software* **71**, 1–25 (2016).

24. Xie, Y., Cheng, J. & Tan, X. *DT: A wrapper of the JavaScript library 'DataTables'*. (2020).

25. Bolker, B. *emdbook: Ecological models and data in R*. (2020).

26. Revelle, W. *Psych: Procedures for psychological, psychometric, and personality research*. (Northwestern University, 2020).

27. Izrailev, S. *tictoc: Functions for timing R scripts*. (2014).

28. Spiegelhalter, D. J., Myles, J. P., Jones, D. R. & Abrams, K. R. Bayesian methods in health technology assessment: A review. *Health Technology Assessment* **4**, 1–130 (2000).

29. Gatsonis, C. & Goodman, S. BaSiS – Bayesian standards in science – standards for reporting of Bayesian analyses in the scientific literature. (2001).

30. Sung, L. *et al.* Seven items were identified for inclusion when reporting a Bayesian analysis of a clinical study. *Journal of Clinical Epidemiology* **58**, 261–268 (2005).

31. Kruschke, J. K. *Doing Bayesian data analysis: A tutorial with R and BUGS*. (Academic Press / Elsevier, 2011).

32. Lang, T. A. & Altman, D. G. Basic statistical reporting for articles published in biomedical journals: The 'statistical analyses and methods in the published literature' or the SAMPL guidelines. *International Journal of Nursing Studies* **52**, 5–9 (2015).

33. Baldwin, S. A. & Larson, M. J. An introduction to using Bayesian linear regression with clinical data. *Behaviour research and therapy* **98**, 58–75 (2017).

34. Depaoli, S. & van de Schoot, R. Improving transparency and replication in Bayesian statistics: The WAMBS-checklist. *Psychological Methods* **22**, 240–261 (2017).

35. van de Schoot, R., Veen, D., Smeets, L., Winter, S. D. & Depaoli, S. A tutorial on using the WAMBS checklist to avoid the misuse of Bayesian statistics. in *Small sample size solutions* (eds. van de Schoot, R. & Miocevic, M.) 30–49 (Routledge, 2020).

36. Appelbaum, M. *et al.* Journal article reporting standards for quantitative research in psychology: The APA Publications and Communications Board task force report. *American Psychologist* **73**, 3–25 (2018).

37. van Doorn, J. *et al.* The JASP guidelines for conducting and reporting a Bayesian analysis. *Psychonomic Bulletin & Review* **28**, 1–14 (2020).

38. Aczel, B. *et al.* Discussion points for Bayesian inference. *Nature Human Behaviour* **4**, 561–563 (2020).
